# Revision of the Malagasy *Camponotus edmondi* species group (Hymenoptera, Formicidae, Formicinae): integrating qualitative morphology and multivariate morphometric analysis

**DOI:** 10.3897/zookeys.572.7177

**Published:** 2016-03-15

**Authors:** Jean Claude Rakotonirina, Sándor Csősz, Brian L. Fisher

**Affiliations:** 1 Madagascar Biodiversity Center, BP 6257, Parc Botanique et Zoologique de Tsimbazaza, Antananarivo, Madagascar; 2 Département d’Entomologie, Faculté des Sciences, BP 906, Université d’Antananarivo, Antananarivo, Madagascar; 3 Entomology, California Academy of Sciences, 55 Music Concourse Drive, San Francisco, CA 94118, U.S.A.

**Keywords:** Allometry, *Camponotus*, exploratory data analysis, Madagascar, Malagasy region, multivariate statistics, morphometrics, taxonomy

## Abstract

The Malagasy *Camponotus edmondi* species group is revised based on both qualitative morphological traits and multivariate analysis of continuous morphometric data. To minimize the effect of the scaling properties of diverse traits due to worker caste polymorphism, and to achieve the desired near-linearity of data, morphometric analyses were done only on minor workers. The majority of traits exhibit broken scaling on head size, dividing *Camponotus* workers into two discrete subcastes, minors and majors. This broken scaling prevents the application of algorithms that uses linear combination of data to the entire dataset, hence only minor workers were analyzed statistically. The elimination of major workers resulted in linearity and the data meet required assumptions. However, morphometric ratios for the subsets of minor and major workers were used in species descriptions and redefinitions. Prior species hypotheses and the goodness of clusters were tested on raw data by confirmatory linear discriminant analysis. Due to the small sample size available for some species, a factor known to reduce statistical reliability, hypotheses generated by exploratory analyses were tested with extreme care and species delimitations were inferred via the combined evidence of both qualitative (morphology and biology) and quantitative data. Altogether, fifteen species are recognized, of which 11 are new to science: *Camponotus alamaina* **sp. n.**, *Camponotus androy* **sp. n.**, *Camponotus bevohitra* **sp. n.**, *Camponotus galoko* **sp. n.**, *Camponotus matsilo* **sp. n.**, *Camponotus mifaka* **sp. n.**, *Camponotus orombe* **sp. n.**, *Camponotus tafo* **sp. n.**, *Camponotus tratra* **sp. n.**, *Camponotus varatra* **sp. n.**, and *Camponotus zavo* **sp. n.** Four species are redescribed: *Camponotus echinoploides* Forel, *Camponotus edmondi* André, *Camponotus ethicus* Forel, and *Camponotus robustus* Roger. *Camponotus edmondi ernesti* Forel, **syn. n.** is synonymized under *Camponotus edmondi*. This revision also includes an identification key to species for both minor and major castes, information on geographic distribution and biology, taxonomic discussions, and descriptions of intraspecific variation. Traditional taxonomy and multivariate morphometric analysis are independent sources of information which, in combination, allow more precise species delimitation. Moreover, quantitative characters included in identification keys improve accuracy of determination in difficult cases.

## Introduction

The ant genus *Camponotus* Mayr, 1861 is one of the most species-rich genera in the world, in the ranks of *Pheidole* (1002 species), *Strumigenys* (838 species), and *Tetramorium* (567 species) (Bolton 2015). It currently includes 1589 valid extant species and subspecies (Bolton 2015) distributed across the tropical and subtropical regions as well as the temperate zones (Bolton 1995). In the southwestern Indian Ocean region, 78 species and subspecies have been described in publications (e.g.: Roger 1863; André 1887; Forel 1891, 1897, 1914; Emery 1896, 1920; Santschi 1921; Wheeler 1922; Donisthorpe 1949); since 1949, no additional species have been described. Over the past two decades, however, the number of *Camponotus* samples has greatly increased thanks to recent intensive research surveys of ants in Madagascar and surrounding islands. A preliminary study (Fisher 1997) showed that *Camponotus* is one of the most diverse genera in the region. Its members occupy a wide variety of microhabitats across different terrestrial ecosystems in Madagascar and neighboring islands. This high diversity suggests that the genus is in great need of comprehensive taxonomic revision to improve the understanding and management of the region’s biodiversity.

As stated by Brown (1973), the subgeneric classifications of *Camponotus* made by earlier ant taxonomists (e.g.: Emery 1896, 1920, 1925; Forel 1914; Santschi 1921; Wheeler 1922) were not useful because numerous unrelated taxa had been combined within many of these subgenera. An initial morphology-based study of the Malagasy *Camponotus* subgenera supported this view. For instance, Santschi (1921) created the subgenus *Myrmepinotus* Santschi for one species from Madagascar. In 1925, Emery transferred into this subgenus three other Malagasy species, all of which had previously been moved from three of the following subgenera: *Orthonotomyrmex* Ashmead (1906), *Myrmentoma* Forel (1912), *Myrmobrachys* Forel (1912), and *Myrmisolepis* Santschi (1921). These four species, when combined with the other new species included in the present study, may constitute unrelated groups of taxa in the subgenus *Myrmepinotus*. A few species might be more closely related to the species within *Myrmisolepis* of the afrotropical and Ethiopian regions than those of the Malagasy *Myrmepinotus*. To avoid following an unsupported subgeneric classification, we instead use a species group classification. The present study undertakes a species-level taxonomic revision of the *Camponotus edmondi* species group of the Malagasy region.

More in-depth comparative taxonomic works, incorporating different sources of data to identify and recognize species, have been initiated in the region (e.g.: Fisher and Smith 2008; Yoshimura and Fisher 2012; Blaimer and Fisher 2013; Hita-Garcia and Fisher 2014; Rakotonirina and Fisher 2014). The present revision combines qualitative differences in morphological characters and multivariate statistical methods of recording morphometric measurement data to delimit and recognize species. Multivariate morphometric analysis combines (1) formation of species hypothesis by exploratory data analysis with (2) hypothesis testing through confirmative linear discriminant analysis (LDA). Not only has it proved efficient as a tool for assessing differences between similar ant taxa, but it also has helped unravel the cryptic diversity in different groups of ants using primary data on size measurements (Csősz et al. 2014; Seifert et al. 2014a, 2014b). The statistical investigation of morphological character variation among species, as is found in highly diverse genera like *Camponotus*, will provide independent information that helps assign individual specimens to a species, facilitate species recognition, and improve precision of species delimitation.

The *Camponotus edmondi* species group can be distinguished by the combination of the following characters: dorsolateral margin of propodeum marginate or extending into a sharp ridge, propodeal declivity usually concave, anterolateral corner of pronotum most often marginate, forecoxa larger than the width of mesopleuron, and usually propodeal dorsum abruptly sloping down to the insertion of the petiole. Our preliminary examinations conducted on the *edmondi* species group indicated that the group comprises a great number of morphologically similar species with highly polymorphic worker castes that are difficult to separate based on general qualitative traits alone. Thus, the *edmondi* species group is ideal for testing the value of combining qualitative morphology and morphometric methods. The current study tested whether multivariate morphometric analysis could clearly resolve species in the *edmondi* species group.

In this paper, 15 species of the *edmondi* species group are recognized on the basis of combined evidence of multivariate analyses of quantitative morphology and qualitative morphological data of worker caste. The use of multivariate morphometric analysis allowed the recognition of masked morphological traits that are useful in species delimitation. The application of conventional, morphology-based taxonomy in combination with multivariate morphometric study will reinforce the placement of taxonomic works as a basis for understanding and sustainably managing biodiversity.

## Materials and methods

### Abbreviation of depositories

CASC
California Academy of Sciences, San Francisco, CA, USA 


MHNG
 Musée d’Histoire Naturelle, Geneva, Switzerland 


MNHN
Musée National d’Histoire Naturelle, Paris, France 


MSNG
Museo Civico di Storia Naturale “Giacomo Doria”, Genoa, Italy 


NHMB
Naturhistorisches Museum, Basel, Switzerland 


PBZT
Parc Botanique et Zoologique de Tsimbazaza, Antananarivo, Madagascar 


PSWC
 P.S. Ward Collection, University of California at Davis, CA, USA 


ZMHB
Museum für Naturkunde der Humboldt Universität, Berlin, Germany 


### Materials

The present contribution includes all specimens of the *Camponotus edmondi* species group collected from the arthropod survey project conducted in Madagascar and surrounding islands in the Malagasy region by B.L. Fisher and the members of the Madagascar Biodiversity Center from 1992 through 2015. All pinned specimens examined in this study are available on the web portal AntWeb (http://www.antweb.org) and can be accessed using the unique identifying specimen code (e.g. CASENT0104547) assigned to specimen for each pin. Images are linked to their specimens via their unique specimen code, which is affixed to each pin (CASENT0002660).

A total of 292 specimens from 168 collecting events has been measured in this study (see Suppl. material 1). Due to the fact that samples from a single collecting event might not represent nest samples (colonies), but specimens mounted on one pin do, collection codes (BLF, MG, or ANTC numbers) were used as grouping factors in NC-clustering.

### Methods

Morphological examinations were conducted to study patterns of morphological discontinuities and phenotypic similarity using a Leica MZ12 binocular microscope.

Digital color images of lateral and dorsal views of the entire body and full-face views of the head of each species were created using a JVC KY-75 or a Leica DFC450 digital camera with a Leica Z16 APO microscope and LAS (v3.8) software. These images are also available online on AntWeb (www.antweb.org) and are accessible using the unique identifying specimen code.

Distribution maps for all species were generated by importing specimen distribution records into the Diva GIS program (Hijmans et al. 2011). Older and type specimens with inadequate geographic coordinates were excluded from these maps.

Article 74 in the ICZN’s code states that the designation of a lectotype from syntype specimens which directly match the original description of a named species is necessary to stabilize the nomenclature. As a consequence, the phrase “present designation” is used to indicate a lectotype. New species epithets used in the present work are arbitrary combinations of letters and thus invariant, as are genitive nouns or nominative singular nouns in apposition.

### Measurements

Morphometric measurements were taken using a Leica MZ 12 stereomicroscope equipped with a cross-scaled ocular micrometer and an orthogonal pair of micrometers. All measurements and indices are presented as arithmetic means and ranges are shown as minimum and maximum values in parentheses. Body size dimensions are expressed in millimeters (mm) and all values were rounded to the second decimal place.

**Figure 1.**
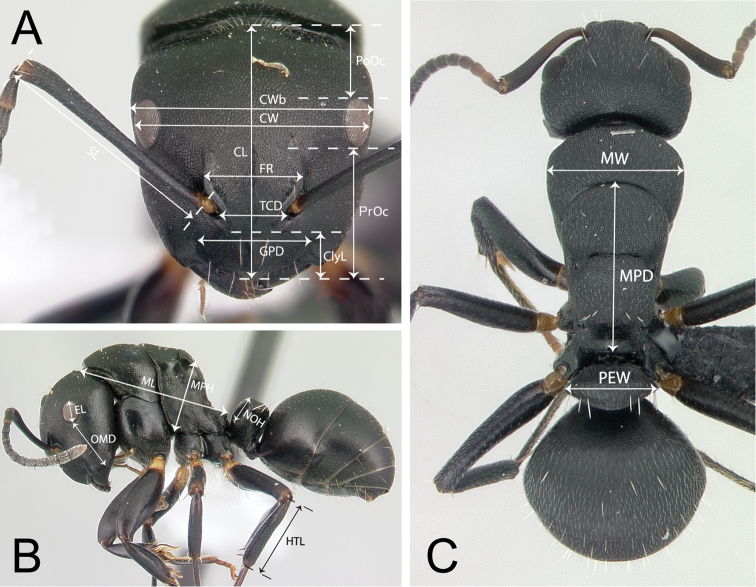
Illustrations of measurements for *edmondi* species group. **A** Head in full-face view **B** Body in lateral view **C** Body in dorsal view. See text for abbreviations.

The following 19 morphometric measurements were taken (Figure 1):

1. Maximum cephalic length (CL): The maximum midline length of the head in full-face view, measured from the midpoint of the posterior margin to the midpoint of the anterior margin of the clypeus.
2. Maximum cephalic width (CW): The maximum distance between the lateral margins of the compound eyes in full-face view.
3. Maximum head capsule width (CWb): The maximum width of the head excluding the compound eyes.
4. Postocular distance (PoOc): The distance between the posteromedian margin of the head and the level of the posterior margin of the compound eyes measured along the midline of the head in full-face view.
5. Preocular distance (PrOc): The distance between the anteromedian margin of the clypeus and the level of the anterior margin of the compound eyes measured along the midline of the head in full-face view.
6. Clypeal length (ClyL): the maximum midline length of the clypeus measured from the posterior margin to the anterior margin in frontal view, in which the anterior and posterior clypeal margins are aligned to same focus. Median concavity on either or both margins reduces the length of the clypeus.
7. Frontal carina distance (FR): The maximum distance between the frontal carinae.
8. Torular carina distance (TCD): The minimum distance between the torular arches that surround the antennal insertion.
9. Maximum tentorial pit distance (GPD): The greatest distance between the centers of the fossae located at or very close to the posterolateral margin of the clypeus.
10. Scape length (SL): Straight line length of the first antennal segment excluding the basal condyle.
11. Eye length (EL): Maximum diameter of the compound eye.
12. Oculo-mandibular distance (OMD): The smallest distance between the anterior margin of the compound eye and the mandibular insertion to the head.
13. Mesosoma width (MW): Maximum width of the pronotum in dorsal view, which in the *Camponotus edmondi* group is also the maximum mesosomal width (hence “mesosoma width”).
14. Mesosoma length (ML): The longest median anatomical line that connects the posteriormost point of the propodeal lobe with the anteriormost point of pronotal collar; preferentially measured in lateral view, but if one of the reference points is not visible dorsal view may help.
15. Mesothoracico-propodeal distance (MPD): With the promesonotal suture and the anterior petiolar foramen margin in the same plane of focus in dorsal view, the maximum midline length between the promesonotal suture and the posteriormost point of the propodeal process dorsal to the petiolar insertion.
16. Mesothoracico-propodeal height (MPH): With the mesosoma in lateral view, the length of the line between the anteroventral corner of the mesopleuron, dorsal to the insertion of the mesocoxa, and the dorsalmost point of the propodeum that is crossed by the measured line. The line is perpendicular to the diagonal line of the mesosoma that connects the anteriormost point of the pronotal shield and the posteriormost point of the propodeal process dorsal to the petiolar insertion, in lateral view.
17. Maximum hind tibia length (HTL): Straight line length of the hind tibia measured from the constriction immediately before its proximal insertion to its distalmost point, excluding the bristles or spines.
18. Petiolar width (PEW): The maximum width of the petiole in dorsal view.
19. Petiolar node height (NOH): The maximum distance between the petiolar spiracle and the dorsalmost point of the petiolar node.
20. Cephalic size (CS): the arithmetic mean of CL and CWb.

### Morphometric data analyses The datasets

The datasets assessed in the present study consist of (1) the primary measurement data of the 19 morphological characters, which represent the general size of each individual specimen measured (See Table of basic measurements of the specimens in the Suppl. material 1), and (2) the ratios of measurements involving the comparison of one measured trait (variable) over another (CS) to show the body proportions or shape of the specimen (Table 1).

**Table 1.** Ratios of morphometric data for minors and majors of the species. Upper line: mean of ratios ± standard deviation, lower line in square brackets: minimum and maximum values. Note: if only two specimens were available then minimum, maximum values are given.

Species	Worker castes	CS	CWb/CL	CW/CL	PoOC/CL	PrOc/CL	ClyL/CL	FR/CS	TCD/CS	GPD/CS	SL/CS
*alamaina*	minors (n = 52)	1.28 ± 0.1	0.83 ± 0	0.93 ± 0	0.23 ± 0	0.55 ± 0	0.3 ± 0	0.39 ± 0	0.29 ± 0	0.46 ± 0	1.1 ± 0
		[0.99, 1.6]	[0.79, 0.91]	[0.89, 0.97]	[0.2, 0.27]	[0.53, 0.58]	[0.27, 0.33]	[0.37, 0.42]	[0.27, 0.31]	[0.39, 0.5]	[1.02, 1.18]
	majors (n = 24)	1.98 ± 0.2	0.95 ± 0	0.91 ± 0	0.26 ± 0	0.52 ± 0	0.33 ± 0	0.38 ± 0	0.26 ± 0	0.37 ± 0	0.75 ± 0.1
		[1.65, 2.22]	[0.87, 1]	[0.85, 0.95]	[0.24, 0.28]	[0.49, 0.55]	[0.31, 0.36]	[0.36, 0.41]	[0.25, 0.28]	[0.35, 0.4]	[0.69, 0.89]
*androy*	minors (n = 11)	0.96 ± 0.1	0.8 ± 0	0.92 ± 0	0.2 ± 0	0.54 ± 0	0.31 ± 0	0.38 ± 0	0.28 ± 0	0.48 ± 0	1.03 ± 0
		[0.87, 1.03]	[0.78, 0.83]	[0.9, 0.96]	[0.19, 0.22]	[0.53, 0.55]	[0.29, 0.32]	[0.36, 0.39]	[0.27, 0.29]	[0.46, 0.51]	[0.97, 1.1]
	majors (n = 6)	1.67 ± 0.1	0.81 ± 0	0.8 ± 0	0.25 ± 0	0.57 ± 0	0.4 ± 0	0.37 ± 0	0.28 ± 0	0.34 ± 0	0.69 ± 0
		[1.58, 1.74]	[0.8, 0.83]	[0.79, 0.81]	[0.24, 0.26]	[0.56, 0.58]	[0.38, 0.41]	[0.36, 0.39]	[0.27, 0.29]	[0.32, 0.35]	[0.67, 0.71]
*bevohitra*	minors (n = 9)	1.14 ± 0.2	0.81 ± 0	0.89 ± 0	0.2 ± 0	0.59 ± 0	0.34 ± 0	0.39 ± 0	0.31 ± 0	0.48 ± 0	1.01 ± 0.1
		[0.99, 1.5]	[0.78, 0.85]	[0.82, 0.94]	[0.18, 0.21]	[0.57, 0.62]	[0.31, 0.38]	[0.37, 0.41]	[0.3, 0.32]	[0.42, 0.51]	[0.84, 1.11]
	majors (n = 6)	1.48 ± 0.1	0.8 ± 0	0.83 ± 0	0.22 ± 0	0.6 ± 0	0.37 ± 0	0.37 ± 0	0.29 ± 0	0.42 ± 0	0.81 ± 0.1
		[1.34, 1.59]	[0.79, 0.81]	[0.81, 0.86]	[0.21, 0.23]	[0.58, 0.63]	[0.35, 0.4]	[0.36, 0.39]	[0.28, 0.31]	[0.4, 0.45]	[0.75, 0.91]
*echinoploides*	minors (n = 20)	1.51 ± 0.1	0.96 ± 0	0.96 ± 0	0.25 ± 0	0.56 ± 0	0.29 ± 0	0.39 ± 0	0.27 ± 0	0.46 ± 0	0.95 ± 0
		[1.24, 1.67]	[0.94, 1]	[0.93, 1]	[0.23, 0.27]	[0.52, 0.6]	[0.26, 0.33]	[0.3, 0.42]	[0.26, 0.29]	[0.43, 0.48]	[0.9, 1.05]
	majors (n = 2)	[2.48, 2.67]	[1.01, 1.02]	[0.9, 0.91]	[0.31, 0.33]	[0.51, 0.51]	[0.31, 0.32]	[0.39, 0.4]	[0.25, 0.27]	[0.35, 0.37]	[0.6, 0.62]
*edmondi*	minors (n = 15)	1.26 ± 0.1	0.9 ± 0	0.88 ± 0	0.23 ± 0	0.55 ± 0	0.31 ± 0	0.39 ± 0	0.28 ± 0	0.44 ± 0	1 ± 0
		[1.12, 1.47]	[0.87, 0.93]	[0.79, 0.9]	[0.21, 0.25]	[0.52, 0.58]	[0.28, 0.34]	[0.37, 0.41]	[0.27, 0.29]	[0.39, 0.46]	[0.89, 1.06]
	majors (n = 8)	1.87 ± 0.1	0.97 ± 0	0.89 ± 0	0.28 ± 0	0.51 ± 0	0.33 ± 0	0.39 ± 0	0.27 ± 0	0.37 ± 0	0.72 ± 0
		[1.79, 1.95]	[0.94, 1]	[0.85, 0.92]	[0.27, 0.29]	[0.5, 0.53]	[0.32, 0.34]	[0.38, 0.4]	[0.26, 0.28]	[0.35, 0.39]	[0.7, 0.75]
*ethicus*	minors (n = 11)	2.21 ± 0.2	0.92 ± 0	0.84 ± 0	0.24 ± 0	0.57 ± 0	0.31 ± 0	0.31 ± 0	0.25 ± 0	0.41 ± 0	1.14 ± 0.1
		[1.92, 2.58]	[0.87, 0.99]	[0.82, 0.86]	[0.21, 0.25]	[0.54, 0.59]	[0.29, 0.33]	[0.29, 0.33]	[0.24, 0.26]	[0.37, 0.44]	[1, 1.26]
	majors (unknown)										
*galoko*	minors (n = 11)	1.15 ± 0.1	0.97 ± 0	0.98 ± 0	0.23 ± 0	0.55 ± 0	0.29 ± 0	0.43 ± 0	0.29 ± 0	0.46 ± 0	0.9 ± 0.1
		[0.93, 1.34]	[0.94, 0.99]	[0.96, 1.01]	[0.2, 0.25]	[0.53, 0.57]	[0.27, 0.31]	[0.41, 0.45]	[0.28, 0.31]	[0.43, 0.49]	[0.81, 0.98]
	majors (n = 5)	1.57 ± 0.2	1 ± 0	0.93 ± 0	0.3 ± 0	0.52 ± 0	0.31 ± 0	0.42 ± 0	0.28 ± 0	0.4 ± 0	0.7 ± 0
		[1.32, 1.72]	[0.96, 1.03]	[0.9, 0.95]	[0.27, 0.31]	[0.5, 0.53]	[0.3, 0.32]	[0.41, 0.42]	[0.27, 0.29]	[0.38, 0.42]	[0.65, 0.76]
*matsilo*	minors (n = 11)	1.11 ± 0.1	0.9 ± 0	0.93 ± 0	0.21 ± 0	0.6 ± 0	0.32 ± 0	0.45 ± 0	0.31 ± 0	0.47 ± 0	0.91 ± 0
		[1, 1.33]	[0.88, 0.94]	[0.91, 0.95]	[0.18, 0.23]	[0.57, 0.62]	[0.29, 0.33]	[0.43, 0.47]	[0.3, 0.32]	[0.44, 0.49]	[0.87, 0.94]
	majors (n = 2)	[2.21, 2.29]	[0.96, 0.96]	[0.87, 0.88]	[0.28, 0.29]	[0.55, 0.55]	[0.34, 0.36]	[0.43, 0.43]	[0.3, 0.3]	[0.35, 0.36]	[0.59, 0.6]
*mifaka*	minors (n = 13)	1.15 ± 0.1	0.93 ± 0	0.92 ± 0	0.23 ± 0	0.56 ± 0	0.32 ± 0	0.41 ± 0	0.29 ± 0	0.47 ± 0	0.97 ± 0
		[1.05, 1.23]	[0.9, 0.95]	[0.9, 0.95]	[0.21, 0.25]	[0.54, 0.58]	[0.29, 0.34]	[0.4, 0.43]	[0.28, 0.31]	[0.45, 0.48]	[0.92, 1.02]
	majors (unknown)										
*orombe*	minors (n = 2)	0.97, 1.06	0.88, 0.88	0.93, 0.94	0.23, 0.24	0.54, 0.56	0.3, 0.3	0.4, 0.41	0.28, 0.29	0.48, 0.49	1, 1.03
	majors (n = 1)	1.82	0.98	0.91	0.3	0.5	0.32	0.4	0.28	0.38	0.67
*robustus*	minors (n = 9)	2.21 ± 0.2	0.94 ± 0	0.86 ± 0	0.24 ± 0	0.57 ± 0	0.31 ± 0	0.35 ± 0	0.25 ± 0	0.44 ± 0	0.96 ± 0
		[1.88, 2.64]	[0.91, 0.98]	[0.84, 0.88]	[0.21, 0.27]	[0.54, 0.59]	[0.29, 0.33]	[0.34, 0.37]	[0.25, 0.26]	[0.43, 0.45]	[0.91, 1.02]
	majors (n = 9)	3.49 ± 0.3	1.01 ± 0	0.83 ± 0	0.28 ± 0	0.54 ± 0	0.33 ± 0	0.33 ± 0	0.23 ± 0	0.35 ± 0	0.69 ± 0
		[2.84, 3.73]	[0.98, 1.02]	[0.81, 0.87]	[0.26, 0.3]	[0.52, 0.55]	[0.32, 0.34]	[0.31, 0.34]	[0.23, 0.24]	[0.32, 0.37]	[0.64, 0.77]
*tafo*	minors (n = 8)	1.11 ± 0.1	0.93 ± 0	0.96 ± 0	0.21 ± 0	0.58 ± 0	0.3 ± 0	0.41 ± 0	0.29 ± 0	0.48 ± 0	1.05 ± 0.1
		[0.97, 1.26]	[0.91, 0.97]	[0.93, 0.99]	[0.19, 0.22]	[0.54, 0.59]	[0.28, 0.34]	[0.39, 0.42]	[0.28, 0.3]	[0.46, 0.5]	[0.96, 1.13]
	majors (unknown)										
*tratra*	minors (n = 8)	1.11 ± 0.1	0.93 ± 0	0.96 ± 0	0.21 ± 0	0.58 ± 0	0.3 ± 0	0.41 ± 0	0.29 ± 0	0.48 ± 0	1.05 ± 0.1
		[0.97, 1.26]	[0.91, 0.97]	[0.93, 0.99]	[0.19, 0.22]	[0.54, 0.59]	[0.28, 0.34]	[0.39, 0.42]	[0.28, 0.3]	[0.46, 0.5]	[0.96, 1.13]
	majors (n = 1)	1.58	0.99	0.91	0.3	0.52	0.32	0.37	0.26	0.4	0.75
*varatra*	minors (n = 16)	0.95 ± 0.1	0.92 ± 0	0.97 ± 0	0.22 ± 0	0.57 ± 0	0.3 ± 0	0.42 ± 0	0.29 ± 0	0.49 ± 0	1.01 ± 0
		[0.85, 1.11]	[0.83, 0.96]	[0.9, 1.03]	[0.2, 0.24]	[0.55, 0.59]	[0.28, 0.32]	[0.4, 0.45]	[0.26, 0.3]	[0.46, 0.52]	[0.93, 1.07]
	majors (n = 16)	1.46 ± 0.1	0.98 ± 0	0.93 ± 0	0.27 ± 0	0.51 ± 0	0.33 ± 0	0.41 ± 0	0.28 ± 0	0.4 ± 0	0.72 ± 0
		[1.35, 1.62]	[0.95, 1]	[0.91, 0.98]	[0.24, 0.29]	[0.48, 0.55]	[0.3, 0.35]	[0.4, 0.43]	[0.25, 0.29]	[0.37, 0.42]	[0.66, 0.78]
*zavo*	minors (n = 14)	1.12 ± 0.1	0.96 ± 0	0.97 ± 0	0.2 ± 0	0.57 ± 0	0.3 ± 0	0.41 ± 0	0.28 ± 0	0.48 ± 0	1.11 ± 0.1
		[1.02, 1.3]	[0.93, 1]	[0.93, 1]	[0.18, 0.23]	[0.54, 0.62]	[0.27, 0.33]	[0.39, 0.43]	[0.25, 0.3]	[0.44, 0.54]	[1, 1.22]
	majors (n = 3)	1.74 ± 0.1	1.02 ± 0	0.92 ± 0	0.26 ± 0	0.54 ± 0	0.3 ± 0	0.39 ± 0	0.26 ± 0	0.39 ± 0	0.79 ± 0
		[1.65, 1.81]	[1.01, 1.04]	[0.89, 0.94]	[0.24, 0.27]	[0.52, 0.55]	[0.29, 0.32]	[0.36, 0.42]	[0.25, 0.27]	[0.38, 0.39]	[0.76, 0.82]

Species	Worker castes	EL/CS	OMD/CS	MW/CS	PEW/CS	MPD/CS	HTL/CS	ML/CS	MPH/CS	NOH/CS
*alamaina*	minors (n = 52)	0.3 ± 0	0.46 ± 0	0.83 ± 0	0.59 ± 0	1.17 ± 0.1	1.09 ± 0.1	1.6 ± 0.1	0.81 ± 0	0.39 ± 0
		[0.26, 0.32]	[0.42, 0.49]	[0.69, 0.89]	[0.47, 0.66]	[0.91, 1.26]	[0.81, 1.23]	[1.32, 1.67]	[0.63, 0.87]	[0.32, 0.46]
	majors (n = 24)	0.25 ± 0	0.45 ± 0	0.68 ± 0.1	0.45 ± 0	0.96 ± 0	0.82 ± 0	1.31 ± 0.1	0.64 ± 0	0.31 ± 0
		[0.23, 0.27]	[0.42, 0.47]	[0.62, 0.91]	[0.41, 0.5]	[0.85, 1.08]	[0.77, 0.93]	[1.24, 1.45]	[0.59, 0.72]	[0.28, 0.35]
*androy*	minors (n = 11)	0.35 ± 0	0.44 ± 0	0.83 ± 0	0.59 ± 0	1.13 ± 0	1.04 ± 0	1.56 ± 0	0.75 ± 0	0.4 ± 0
		[0.34, 0.38]	[0.43, 0.46]	[0.8, 0.85]	[0.55, 0.63]	[1.1, 1.16]	[1, 1.09]	[1.52, 1.6]	[0.72, 0.79]	[0.36, 0.49]
	majors (n = 6)	0.28 ± 0	0.45 ± 0	0.65 ± 0	0.44 ± 0	0.93 ± 0	0.76 ± 0	1.26 ± 0	0.59 ± 0	0.28 ± 0
		[0.26, 0.29]	[0.43, 0.46]	[0.64, 0.66]	[0.39, 0.48]	[0.91, 0.95]	[0.74, 0.77]	[1.25, 1.27]	[0.56, 0.63]	[0.25, 0.32]
*bevohitra*	minors (n = 9)	0.3 ± 0	0.47 ± 0	0.7 ± 0	0.4 ± 0	1.06 ± 0	1.08 ± 0	1.54 ± 0.1	0.73 ± 0	0.4 ± 0
		[0.27, 0.32]	[0.45, 0.49]	[0.61, 0.74]	[0.38, 0.44]	[0.97, 1.11]	[0.96, 1.12]	[1.43, 1.59]	[0.65, 0.76]	[0.34, 0.44]
	majors (n = 6)	0.27 ± 0	0.49 ± 0	0.62 ± 0	0.35 ± 0	0.98 ± 0	0.95 ± 0	1.39 ± 0	0.62 ± 0	0.35 ± 0
		[0.26, 0.29]	[0.47, 0.51]	[0.6, 0.64]	[0.34, 0.37]	[0.95, 1.02]	[0.9, 1.04]	[1.35, 1.47]	[0.58, 0.64]	[0.31, 0.36]
*echinoploides*	minors (n = 20)	0.21 ± 0	0.48 ± 0	0.95 ± 0	0.6 ± 0.1	1.06 ± 0	0.97 ± 0.1	1.45 ± 0	0.69 ± 0	0.28 ± 0
		[0.19, 0.24]	[0.44, 0.52]	[0.89, 1.01]	[0.54, 0.74]	[1, 1.11]	[0.91, 1.18]	[1.38, 1.51]	[0.6, 0.76]	[0.24, 0.32]
	majors (n = 2)	[0.17, 0.17]	[0.49, 0.49]	[0.68, 0.73]	[0.37, 0.43]	[0.79, 0.81]	[0.71, 0.72]	[1.1, 1.18]	[0.48, 0.58]	[0.18, 0.18]
*edmondi*	minors (n = 15)	0.26 ± 0	0.49 ± 0	0.84 ± 0.1	0.54 ± 0.1	1.07 ± 0.1	0.96 ± 0.1	1.44 ± 0.1	0.73 ± 0	0.3 ± 0
		[0.24, 0.28]	[0.46, 0.51]	[0.74, 1.03]	[0.47, 0.68]	[0.93, 1.42]	[0.88, 1.29]	[1.3, 1.51]	[0.66, 0.81]	[0.28, 0.34]
	majors (n = 8)	0.23 ± 0	0.47 ± 0	0.66 ± 0	0.42 ± 0	0.86 ± 0.1	0.77 ± 0	1.21 ± 0	0.59 ± 0	0.24 ± 0
		[0.22, 0.24]	[0.45, 0.5]	[0.65, 0.68]	[0.38, 0.47]	[0.6, 0.94]	[0.74, 0.81]	[1.16, 1.25]	[0.57, 0.61]	[0.22, 0.27]
*ethicus*	minors (n = 11)	0.23 ± 0	0.51 ± 0	0.86 ± 0.1	0.32 ± 0	1.17 ± 0.1	1.44 ± 0.1	1.72 ± 0.1	0.67 ± 0	0.29 ± 0
		[0.21, 0.25]	[0.49, 0.53]	[0.79, 0.95]	[0.27, 0.36]	[1.1, 1.27]	[1.29, 1.56]	[1.62, 1.86]	[0.64, 0.72]	[0.25, 0.34]
	majors (unknown)									
*galoko*	minors (n = 11)	0.24 ± 0	0.47 ± 0	0.85 ± 0	0.49 ± 0	0.95 ± 0	0.83 ± 0	1.33 ± 0.1	0.64 ± 0	0.27 ± 0
		[0.23, 0.25]	[0.43, 0.48]	[0.79, 0.92]	[0.47, 0.51]	[0.89, 0.98]	[0.78, 0.87]	[1.24, 1.44]	[0.59, 0.67]	[0.24, 0.3]
	majors (n = 5)	0.21 ± 0	0.46 ± 0	0.72 ± 0	0.41 ± 0	0.87 ± 0	0.72 ± 0	1.17 ± 0	0.56 ± 0	0.24 ± 0
		[0.19, 0.22]	[0.46, 0.47]	[0.7, 0.75]	[0.37, 0.44]	[0.84, 0.88]	[0.71, 0.74]	[1.13, 1.21]	[0.52, 0.58]	[0.21, 0.27]
*matsilo*	minors (n = 11)	0.25 ± 0	0.51 ± 0	0.88 ± 0	0.46 ± 0	1.12 ± 0	0.98 ± 0	1.53 ± 0	0.65 ± 0	0.29 ± 0
		[0.24, 0.26]	[0.47, 0.52]	[0.84, 0.91]	[0.42, 0.49]	[1.07, 1.17]	[0.91, 1.02]	[1.44, 1.58]	[0.57, 0.72]	[0.25, 0.34]
	majors (n = 2)	[0.2, 0.21]	[0.48, 0.49]	[0.7, 0.7]	[0.39, 0.4]	[0.94, 0.97]	[0.72, 0.75]	[1.21, 1.26]	[0.55, 0.6]	[0.25, 0.27]
*mifaka*	minors (n = 13)	0.26 ± 0	0.46 ± 0	0.78 ± 0	0.49 ± 0	0.99 ± 0	0.93 ± 0	1.33 ± 0	0.67 ± 0	0.34 ± 0
		[0.25, 0.27]	[0.45, 0.48]	[0.75, 0.82]	[0.47, 0.52]	[0.95, 1.04]	[0.91, 0.99]	[1.29, 1.4]	[0.61, 0.71]	[0.3, 0.38]
	majors (unknown)									
*orombe*	minors (n = 2)	0.26, 0.26	0.44, 0.46	0.8, 0.81	0.45, 0.47	1.07, 1.09	0.87, 0.89	1.43, 1.49	0.67, 0.7	0.28, 0.3
	majors (n = 1)	0.22	0.45	0.64	0.35	0.88	0.69	1.2	0.57	0.24
*robustus*	minors (n = 9)	0.23 ± 0	0.49 ± 0	1 ± 0	0.44 ± 0	1.16 ± 0	1.33 ± 0	1.61 ± 0	0.78 ± 0	0.25 ± 0
		[0.2, 0.25]	[0.47, 0.52]	[0.94, 1.03]	[0.39, 0.48]	[1.1, 1.21]	[1.25, 1.4]	[1.52, 1.65]	[0.73, 0.8]	[0.23, 0.29]
	majors (n = 9)	0.18 ± 0	0.48 ± 0	0.63 ± 0.2	0.34 ± 0	0.89 ± 0	0.96 ± 0	1.24 ± 0	0.55 ± 0	0.18 ± 0
		[0.17, 0.19]	[0.45, 0.5]	[0.22, 0.81]	[0.31, 0.37]	[0.85, 0.95]	[0.91, 1.04]	[1.18, 1.32]	[0.52, 0.61]	[0.13, 0.25]
*tafo*	minors (n = 8)	0.26 ± 0	0.49 ± 0	0.83 ± 0	0.49 ± 0	1.01 ± 0	0.95 ± 0	1.42 ± 0	0.71 ± 0	0.31 ± 0
		[0.24, 0.27]	[0.47, 0.5]	[0.8, 0.87]	[0.45, 0.52]	[0.96, 1.05]	[0.92, 1.01]	[1.37, 1.45]	[0.65, 0.75]	[0.28, 0.33]
	majors (unknown)									
*tratra*	minors (n = 8)	0.26 ± 0	0.49 ± 0	0.83 ± 0	0.49 ± 0	1.01 ± 0	0.95 ± 0	1.42 ± 0	0.71 ± 0	0.31 ± 0
		[0.24, 0.27]	[0.47, 0.5]	[0.8, 0.87]	[0.45, 0.52]	[0.96, 1.05]	[0.92, 1.01]	[1.37, 1.45]	[0.65, 0.75]	[0.28, 0.33]
	majors (n = 1)	0.2	0.47	0.63	0.31	0.8	0.75	1.16	0.5	0.2
*varatra*	minors (n = 16)	0.26 ± 0	0.48 ± 0	0.81 ± 0	0.45 ± 0	0.99 ± 0	0.94 ± 0.1	1.39 ± 0	0.66 ± 0	0.28 ± 0
		[0.23, 0.3]	[0.45, 0.52]	[0.76, 0.84]	[0.42, 0.49]	[0.93, 1.09]	[0.86, 1.2]	[1.31, 1.48]	[0.59, 0.72]	[0.23, 0.35]
	majors (n = 16)	0.23 ± 0	0.46 ± 0	0.67 ± 0	0.37 ± 0	0.86 ± 0	0.73 ± 0	1.19 ± 0	0.57 ± 0	0.24 ± 0
		[0.22, 0.24]	[0.44, 0.48]	[0.65, 0.7]	[0.26, 0.42]	[0.81, 0.92]	[0.68, 0.82]	[1.12, 1.26]	[0.49, 0.6]	[0.2, 0.27]
*zavo*	minors (n = 14)	0.26 ± 0	0.5 ± 0	0.83 ± 0	0.5 ± 0	1.01 ± 0	1.01 ± 0	1.43 ± 0	0.69 ± 0	0.32 ± 0
		[0.24, 0.28]	[0.48, 0.51]	[0.77, 0.88]	[0.45, 0.58]	[0.95, 1.08]	[0.95, 1.05]	[1.38, 1.48]	[0.62, 0.77]	[0.28, 0.37]
	majors (n = 3)	0.21 ± 0	0.49 ± 0	0.66 ± 0	0.39 ± 0	0.84 ± 0	0.77 ± 0.1	1.18 ± 0	0.54 ± 0	0.23 ± 0
		[0.2, 0.23]	[0.48, 0.5]	[0.65, 0.68]	[0.38, 0.4]	[0.82, 0.86]	[0.69, 0.83]	[1.17, 1.2]	[0.52, 0.56]	[0.21, 0.25]

In the present taxonomic revision, multivariate statistical analysis of morphometric data was run to obtain information considered helpful in detecting species and to facilitate species delimitation decisions.

### Data preparation


 Nest-centroid clustering (NC-clustering) and linear discriminant analysis (LDA) do not require special data preparation, however, within-class linearity of correlation (linear scaling) for each trait is assumed.

**Figure 2.**
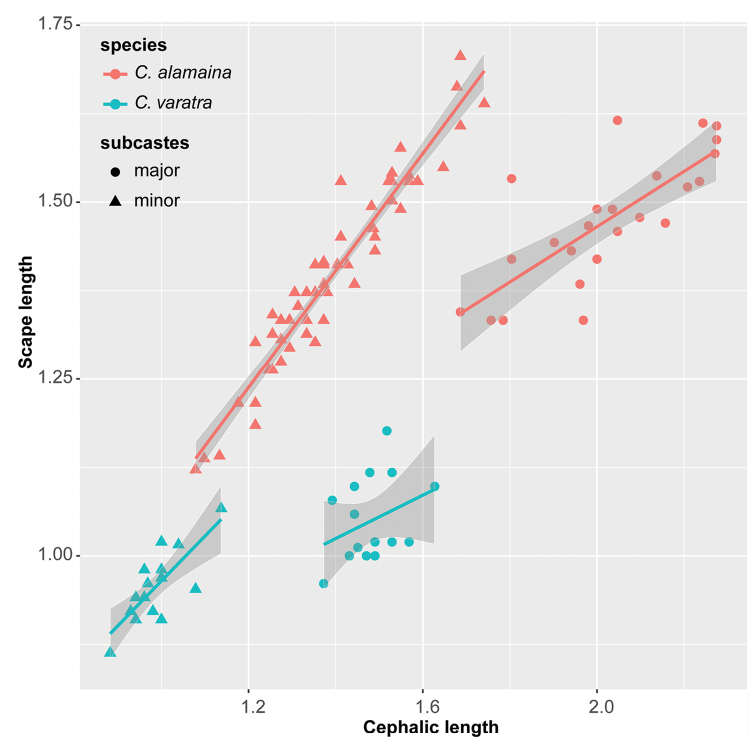
Broken scaling of a morphometric trait, scape length (SL) on cephalic length (CL) is illustrated in two species, *Camponotus alamaina* (red) and *Camponotus varatra* (blue). The scaling break splits the populations into two discrete phenotypes: minors (triangles) and majors (circles). The dark grey areas show confidence interval, the regression line is computed by linear model.

Static trait allometry of variables for workers in the *edmondi* species group has been tested via pair-wise visual inspection of matrix scatterplots. Our results indicate the presence of broken, or sigmoid scaling, i.e. shifts in scaling resulting with different intercepts, steepness, or both, illustrated for CL for 12 of 19 characters, splitting workers into two remarkable subsets, minors and majors (see Figure 2). The broken scaling of traits and the different allometric properties of the subcastes prevented us from analyzing the two groups together. We selected minor workers for morphometric analyses, because these are more abundant in the material examined; 209 out of the 292 workers were minors based on scaling schemes and only 83 proved to be majors.

The Shapiro-Wilk W test was used to test for within-class normality after major workers were eliminated. No characters showed significant deviation from the normal distribution according to the W statistic, so each character can be considered normally distributed. In our statistical analyses we follow Csősz and Fisher (2015).

### Generation of species hypotheses by exploratory data analyses

The classification hypothesis of samples, which is represented by the number of estimated clusters, was built using the exploratory analysis of continuous morphometric data technique. This technique uses an NC-clustering algorithm (Seifert et al. 2014a) that searches for discontinuities in the data and assembles all samples into clusters so that samples within clusters are similar and contrast with those in other clusters. This grouping technique transforms morphological differences between nest samples into a distance matrix in linear discriminant space. The resulting linear discriminant scores are presented as a dendrogram within Euclidian space using the Unweighted Pair Group Method with Arithmetic Mean (UPGMA) distance method. Computations were run in R (R Core Team 2015). NC-clustering was run using packages *cluster* (Maechler et al. 2014) and *MASS* (Venables and Ripley 2002). To assess how reliable the same clusters are with a sub-sampled dataset, a bootstrap analysis was applied by running 100 iterations (method = “average,” method.dist = “Euclidean,” nboot = 100) using package *pvclust* (Suzuki and Shimodaira 2014).

### Hypothesis testing by confirmatory LDA

The confirmative LDA was run repeatedly until the final classification, exhibiting the highest posterior probability values, was produced (Csősz et al. 2014). In addition, the technique was run as an iterative process until the minimum number of characters required to contribute to a desired level (>99%) of classification success was obtained (Seifert 2014a; Seifert et al. 2014b).

Each species was then described using qualitative and quantitative morphological characters of the worker castes (minor and major). An identification key to species is presented based on diagnostic characters of the workers. Morphological terminology follows Bolton (1994) and integument sculpture terminology follows Harris (1979).

## Results and discussion

### Synoptic list of species of the Malagasy *Camponotus edmondi* species group

***alamaina*** Rakotonirina, Csősz & Fisher, sp. n.

***androy*** Rakotonirina, Csősz & Fisher, sp. n.

***bevohitra*** Rakotonirina, Csősz & Fisher, sp. n.

***echinoploides*** Forel, 1891

***edmondi*** André, 1887

= *edmondi* var. *ernesti* Forel, 1891 syn. n.

***ethicus*** Forel, 1897

***galoko*** Rakotonirina, Csősz & Fisher, sp. n.

***matsilo*** Rakotonirina, Csősz & Fisher, sp. n.

***mifaka*** Rakotonirina, Csősz & Fisher, sp. n.

***orombe*** Rakotonirina, Csősz & Fisher, sp. n.

***robustus*** Roger, 1863

***tafo*** Rakotonirina, Csősz & Fisher, sp. n.

***tratra*** Rakotonirina, Csősz & Fisher, sp. n.

***varatra*** Rakotonirina, Csősz & Fisher, sp. n.

***zavo*** Rakotonirina, Csősz & Fisher, sp. n.

### Morphological diagnosis of the worker castes of *Camponotus edmondi* species group

**Figure 3.**
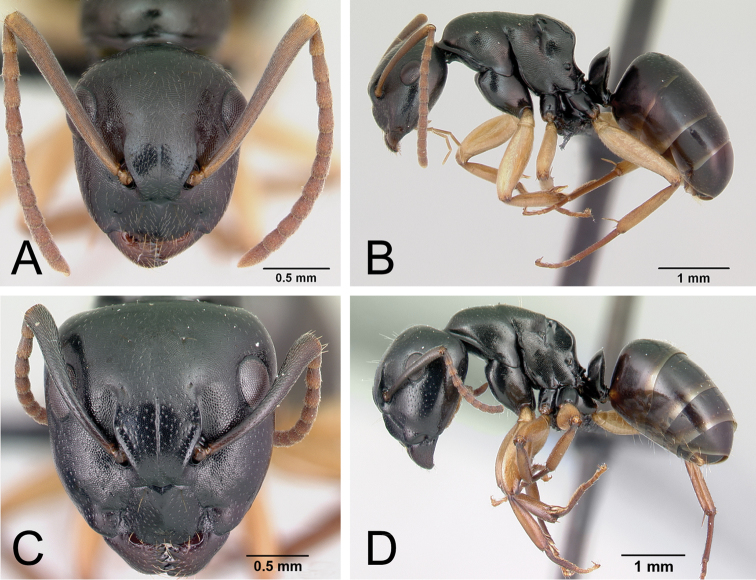
Head in full-face view and body in lateral view of the worker castes of *Camponotus alamaina*. **A, B** minor worker (CASENT0499291) **C, D** major worker (CASENT0179431).

Although the majority of species in the *edmondi* species group are arboreal (13/15), a few species are terrestrial (i.e., build their nests in the ground, in rotten logs, and in dead tree stumps). Sometimes individual workers are found foraging on the forest floor or through leaf litter. Within a colony, two very different worker castes, minor and major workers (see Fig. 3), are observed in the group; between these extremes, various worker forms showing continuous morphological variation occur. The combination of the following features can be used to reliably diagnose the two extreme worker castes relative to other Malagasy species groups.

### Minor worker

1. Mandibles triangular, masticatory margins armed with 6 teeth; basal margins smooth.
2. Palp formula 6,4; palps long with respect to head size.
3. Clypeus with straight (*Camponotus ethicus*, *Camponotus edmondi*), broadly convex, or medially triangular (*Camponotus tafo*) anterior margin in full-face view; median notch present in the posterior margin.
4. Antenna with 12 segments; pedicel longer than the flagellum, length gradually reduced towards the penultimate antennomere; apical portion of the flagellum either lighter or darker in color than the basal portion; antennal scape variable in length.
5. Base of antenna inserted a good distance from posterior margin of clypeus, the distance at least as large as the maximum width of antennal scape.
6. Frontal lobe narrow and partially covering the antennal insertion; frontal carina extended posteriorly at about the level of anterior margin of the eyes in full-face view.
7. Compound eye large, located anterior to the midline of the head in profile view.
8. Head longer than broad; broader posteriorly; posterior margin convex.
9. Pronotum broad with very short anterior face, anterodorsally marginate to carinate; dorsolateral portion slightly to strongly marginate anteriorly; anterior and lateral faces rounding to the dorsum in *Camponotus alamaina*.
10. Promesonotal suture present.
11. Metanotal groove vestigial or slightly impressed (*Camponotus androy* and *Camponotus bevohitra*) to strongly impressed.
12. Metapleuron anteroposteriorly compressed between mesopleuron and propodeum.
13. Propodeum generally marginate dorsolaterally; in lateral view, most of the dorsum abruptly sloping down to the insertion of the petiole; propodeum quadrate (*Camponotus robustus*), with a pair of triangular extensions posteriorly (*Camponotus ethicus* and *Camponotus alamaina*).
14. Propodeal declivity slightly to strongly concave.
15. Propodeal lobe absent.
16. Metapleural gland lacking.
17. Procoxa large, maximum width larger than width of mesopleuron (or at least as large as 2/3 the width of the mesopleuro-propodeal surface together).
18. Tibial spur single and pectinate on mesotibia and metatibia.
19. Petiole generally flattened anteroposteriorly except in *Camponotus echinoploides*; in profile, anterior margin convex and posterior margin either convex or straight; both faces either rounding or tapering dorsally.
20. Sculpture ranging from smooth and shiny superimposed with microreticulation to densely and finely reticulate-punctate or reticulate rugose.
21. Body color varying from light brown to black with lighter gastral segment and even lighter appendages (brown to depigmented yellow).

### Major worker

Most of the features mentioned above for the minor caste are also characteristics of the major caste, except that the latter has the following characteristics: a bigger head, roughly as long as broad in full-face view; lateral cephalic margins gradually narrowed or abruptly converging (*Camponotus echinoploides*) to the base of mandibles; posterior margin more or less straight; both palps and antennal scape short with respect to head size; antennal scape not surpassing posterior cephalic margin; anterior clypeal margin more or less straight; pronotum broad in dorsal view; in dorsal view, metanotum a narrow transverse ridge between metanotal groove and propodeum.

In the Malagasy region, the *Camponotus edmondi* species group can be differentiated from other species of the genus by the combination of the following characters: dorsolateral margin of propodeum marginate or extending into a sharp ridge, propodeal declivity usually concave, anterolateral corner of pronotum most often marginate, forecoxa larger than the width of mesopleuron, and usually the propodeal dorsum abruptly sloping down to the insertion of the petiole.

### Multivariate analysis of morphometrics

**Figure 4.**
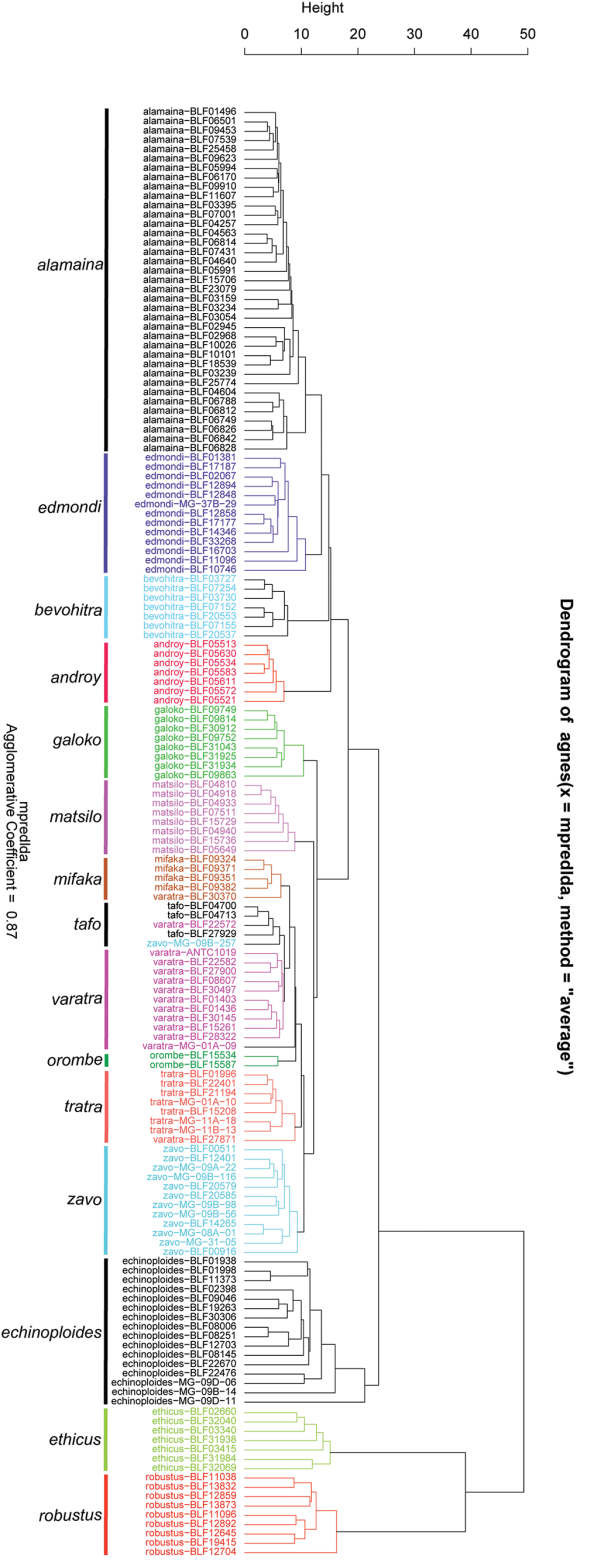
Dendrogram of NC-clustering of the *edmondi* species group. Label on the tip of the branch indicates the species name followed by the specimen code.

The NC-clustering dendrogram using row data revealed 15 clusters (Fig. 4), which are interpreted as 15 species in this revisionary work on the *edmondi* species group. In the dendrogram, three samples of *Camponotus varatra* were placed in each of the following species: *Camponotus mifaka*, *Camponotus tafo*, and *Camponotus tratra*, and one worker of *Camponotus zavo* was embedded in *Camponotus tafo*. The phenomenon may be ascribed to the large difference in sample size between those species: *Camponotus varatra* (n=18), *Camponotus zavo* (n=14) *Camponotus mifakaCamponotus tafo* (n=8), and *Camponotus tratra* (n=8), which hampers the correct placement of these lineages by the phenetic NC-clustering method. In addition, these species are very closely related and overlap in their morphometric and qualitative descriptions.

The 15-species hypothesis has been corroborated by confirmatory LDA at 99.06% (Table 2). Almost all of the species recognized in the present study were correctly classified: *Camponotus alamaina* (100%), *Camponotus androy* (100%), *Camponotus bevohitra* (100%), *Camponotus echinoploides* (100%), *Camponotus edmondi* (100%), *Camponotus ethicus* (100%), *Camponotus matsilo* (100%), *Camponotus mifaka* (100%), *Camponotus orombe* (100%), *Camponotus robustus* (100%), *Camponotus tafo* (100%), *Camponotus tratra* (100%), and *Camponotus zavo* (100%). Only *Camponotus varatra* and *Camponotus galoko* show lower classification success scores of 94.44% and 90.91% respectively.

**Table 2.** Classification matrix of species showing the classification success (percentage), the observed classification (rows) and the predicted classification (columns). Numbers in the matrix are specimen counts.

Species	Classification success (%)	*alamaina*	*androy*	*bevohitra*	*echinoploides*	*edmondi*	*ethicus*	*galoko*	*matsilo*	*mifaka*	*orombe*	*robustus*	*tafo*	*tratra*	*varatra*	*zavo*
*alamaina*	100.00	52														
*androy*	100.00		11													
*bevohitra*	100.00			9												
*echinoploides*	100.00				20											
*edmondi*	100.00					15										
*ethicus*	100.00						11									
*galoko*	90.91							10							1	
*matsilo*	100.00								11							
*mifaka*	100.00									13						
*orombe*	100.00										3					
*robustus*	100.00											9				
*tafo*	100.00												8			
*tratra*	100.00													8		
*varatra*	94.44													1	17	
*zavo*	100.00															14
Total	99.06	52	11	9	20	15	11	10	11	13	3	9	8	9	18	14

Two samples were misclassified by the cumulative LDA: a single individual observed in *Camponotus varatra* sp. n. was classified as *Camponotus tratra* sp. n. with posterior probability p = 0.74, and one of the 11 minor workers of *Camponotus galoko* sp. n. was classified as *Camponotus varatra* sp. n. with posterior probability p = 0.76. However, the low posterior probability values may indicate that these species are very closely related and overlap in their morphometric and qualitative descriptions.

### Combining morphometry with other information for species definition

Taxonomy integrates multiple lines of evidence to better infer species boundaries. In the present study, information obtained from multivariate morphometric analysis consitutes one piece of evidence used to help indicate the presence of reproductive isolation. A few species in the *Camponotus edmondi* group look very similar to each other and some species present significant morphological variation across their geographic distribution, requiring more independent information to achieve species resolution. As an example, the classification success of *Camponotus galoko* is quite low because one sample was misclassified as *Camponotus varatra*, but its status as a species is supported by qualitative morphological traits which were not included in the morphometric approach. Members of the former species are characterized by a densely and finely reticulate integument whereas those of the latter have imbricating sculpture. In addition, *Camponotus galoko* is geographically distributed in the transitional humid forest in the northwest of Madagascar while *Camponotus varatra* mostly occupies the eastern rainforest of the island.

In another case, two specimens of *Camponotus zavo* and *Camponotus varatra* are placed in the cluster of *Camponotus tafo* based on NC-clustering analysis, suggesting that they should be grouped into one species. However, the separation of *Camponotus tafo* is confirmed by biological data (see Distribution and biology in the Species account section) collected on its members, which have been found only in the rainforest canopy of Parc National Masoala. By contrast, the colony nests of *Camponotus zavo* and *Camponotus varatra* are built in dead twigs or branches slightly above the forest floor but never in the canopy. A similar species (*Camponotus mifaka*), which contains one of the minor worker of *Camponotus varatra*, generally nests in the ground under root mat layers.

In contrast to *Camponotus varatra* and *Camponotus zavo*, *Camponotus alamaina* shows qualitative morphological character differences in the shape of the propodeum, the form of the petiolar node, and the color of the legs across its geographical distribution, but is grouped together in one cluster in the dendrogram (see discussion of the three variants in the species account). The grouping in the dendrogram shows no clear separation of the three variants and is supported by the gradual variation of the characters as many specimens are considered intermediate after assessment of numerous samples.

Two minor workers were misclassified by the cumulative LDA. Factors responsible for this are uncertain, but we might not have been able to measure the full range of worker forms representative of the species of concern in the *edmondi* species group. Because individual colonies of the species in the group show strong allometric variation (polymorphism), the range of worker forms from the same nests should be considered in the study to obtain a more robust classification. Moreover, additional morphological characters should be included in the morphometric investigation.

### Identification key to worker caste of the Malagasy *Camponotus edmondi* species group

The following key applies to both minor and major workers.

**Table:** 

1	In profile, anterior margin of petiolar node convex and posterior margin more or less straight; propodeal spiracle located on lateral portion of propodeum, anterior to posterolateral margin of propodeum (Fig. 5A)	**2**
–	In profile, anterior margin of petiolar node convex and posterior margin either convex or roughly triangular; propodeal spiracle located on declivitous surface or at posterolateral margin of the propodeum (Fig. 5B)	**6**
2	Larger species (CS: 1.882–3.725; CL: 1.961–3.686; ML: 3.098–4.667); body color uniformly black to dark brown (Fig. 6A)	3
–	Smaller species (CS: 0.875–2.222; CL: 0.98–2.275; ML: 1.373–2.902); body bicolored, head and mesosoma black to dark brown, gaster and appendages lighter in color (dark brown to depigmented yellow) (Fig. 6B	**4**
3	Level of the propodeal dorsum abruptly lower than level of the promesonotal dorsum; pronotal dorsum with few erect hairs; humeral angle extended anteriorly into a narrow ridge (Fig. 7A)	***ethicus***
–	Level of propodeal dorsum not abruptly lower than level of promesonotal dorsum; pronotum covered with numerous erect hairs and pubescence; humeral angle slightly tuberculate, not extended into a narrow ridge (Fig. 7B)	***robustus***
4	In profile, anterior margin of pronotum broadly rounding to the dorsum; dorsolateral and posterolateral margins of propodeum strongly carinate (Fig. 8A); somewhat larger species (CS: 0.991–2.222; CL: 1.078–2.275; ML: 1.62–2.902)	***alamaina***
–	In profile, anterior margin of pronotum very short and indistinct, the cervical shield apparently joins the pronotal dorsum directly; at least posterolateral margin of propodeum not strongly carinate, but simply marginate or rounded (Fig. 8B); generally smaller species (CS: 0.875–1.739; CL: 0.98–1.922; ML: 1.373–2.235)	**5**
5	In dorsal view, dorsolateral portion of propodeum with sharp carina, posterolateral margin marginate (Fig. 9A); in profile, width of mesopleuron, seen at the level of spiracle, about as large as that of lateral portion of propodeum; at least one pair of erect hairs present on propodeal dorsum	***androy***
–	In dorsal view, dorsal face of propodeum rounded to lateral face, junction without sharp carina, and posterolateral margin rounded (Fig. 9B); in profile mesopleuron, taken at spiracle level, much wider than lateral portion of propodeum; erect hairs lacking on propodeal dorsum	***bevohitra***
6	In profile, propodeum strongly compressed anteroposteriorly, without clear distinction between dorsal margin and declivity (Fig. 10A); in dorsal view, mesonotum broad, at least twice as broad as long (Fig. 10B)	**7**
–	In profile, propodeum not strongly compressed anteroposteriorly, propodeal dorsum and declivitous surface separated by blunt angle (Fig. 10C); in dorsal view, mesonotum narrow, less than twice as broad as long (Fig. 10D)	**8**
7	Posterodorsal corner of mesonotum raised into a bluntly rounded shield (Fig. 11A); somewhat larger species (CS: 1.235–2.667, 1.6; CL: 1.255–2.647, 1.625; ML: 1.843–2.922, 2.257)	***echinoploides***
–	Posterodorsal corner of mesonotum rounded, not forming an extended shield (Fig. 11B); somewhat smaller species (CS: 1–1.722, 1.265; CL: 0.961–1.725, 1.29; ML: 1.341–2.078, 1.623)	***galoko***
8	In profile, straight line connecting one end of dorsolateral carina of propodeum at the metanotal groove to the other end next to propodeal spiracle conspicuously longer than posterolateral margin of propodeum (Fig. 12A)	***matsilo***
–	In profile, straight line connecting one end of dorsolateral carina of propodeum at the metanotal groove to the other end next to the propodeal spiracle approximately as long as posterolateral margin of propodeum (Fig. 12B)	**9**
9	Dorsum of head and mesosoma densely and finely reticulate punctate (Fig. 13A)	**10**
–	Dorsum of head and mesosoma smooth and shiny, superimposed by fine imbrication (Fig. 13B)	**13**
10	Dorsum of mesosoma with numerous erect hairs, pubescence conspicuous (Fig. 14A)	***mifaka***
–	Hairs lacking on dorsum of pronotum; a pair of hairs present on mesonotum; dorsum of propodeum covered with few erect hairs; hairs on propodeum mostly arise along the region separating dorsal surface and declivity; pubescence inconspicuous (Fig. 14B)	**11**
11	Distance between meso-metapleural suture and dorsolateral margin of propodeum remains the same along the dorsolateral carina of propodeum (Fig. 15A); no distinct angle between dorsal margin of propodeum and declivity, both portions apparently forming a straight line	***orombe***
–	Distance between meso-metapleural suture and dorsolateral margin of propodeum variable, largest near the junction of dorsolateral carina and declivitous surface (Fig. 15B); blunt angle or convexity between dorsal margin of propodeum and declivity distinct	**12**
12	With mesosoma in dorsal view, lateral margins of mesonotum roughly straight and gradually converging posteriorly; width of propodeum at the metanotal groove less than half the maximum width of mesonotum (Fig. 16A); with head in full-face view, anteromedian margin of clypeus truncate	***edmondi***
–	With mesosoma in dorsal view, lateral margins of mesonotum convex and strongly converging posteriorly; width of propodeum at metanotal groove greater than half the maximum width of mesonotum (Fig. 16B); with head in full-face view, anteromedian margin of clypeus triangular	***tafo***
13	In profile, mesonotal dorsum strongly sloping down to the level of propodeum, maximum length of mesonotum about as long as distance between metanotal groove and propodeal spiracle (Fig. 17A); in dorsal view, lateral margin of mesonotum not well defined and converging gradually towards metanotal groove (Fig. 17B); head and mesosoma brown	***tratra***
–	In profile, mesonotum slightly sloping down to the level of propodeum, maximum length distinctly shorter than distance between metanotal groove and propodeal spiracle (Fig. 17C); in dorsal view, lateral margin of mesonotum well defined and evenly convex, converging abruptly towards metanotal groove (Fig. 17D); head and mesosoma dark brown to black	**14**
14	In profile, anterodorsal corner of pronotum extending anteriorly into narrow edge but dorsolateral portion not marginate, junction of dorsum to lateral surface always rounded; blunt angle between dorsal margin of propodeum and declivity distinct, or the junction between both portion rounded (Fig. 18A); antennal scape and gastral tergites I-III covered with abundant appressed pubescence (Fig. 18B)	***zavo***
–	In profile, anterodorsal corner of pronotum extending anteriorly into narrow edge and dorsolateral portion marginate; junction of dorsum to lateral surface of pronotum sharply angulate; no distinct angle between dorsal margin of propodeum and declivity, both portions apparently forming a straight line (Fig. 18C); antennal scape and gastral tergites I-III with scattered appressed pubescence (Fig. 18D)	***varatra***

**Figure 5.**
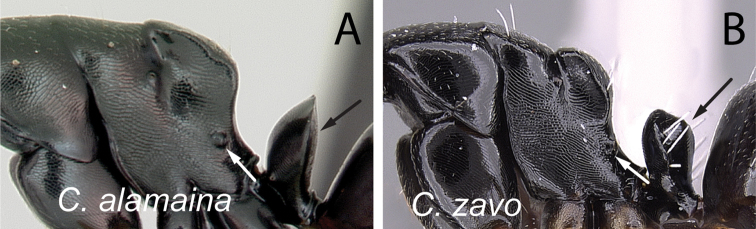
Mesosoma and petiolar node in profile. **A** *Camponotus alamaina* (CASENT0499291) **B** *Camponotus zavo* (CASENT0060041).

**Figure 6.**
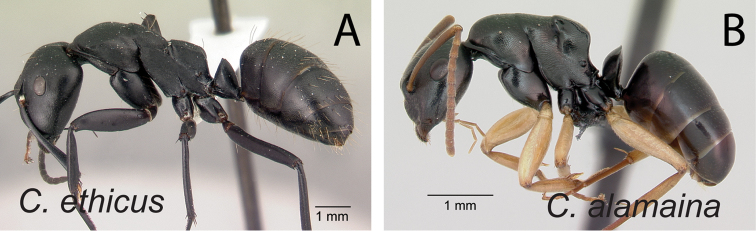
Individual minor worker in profile. **A** *Camponotus ethicus* (CASENT0409948) **B** *Camponotus alamaina* (CASENT0499291).

**Figure 7.**
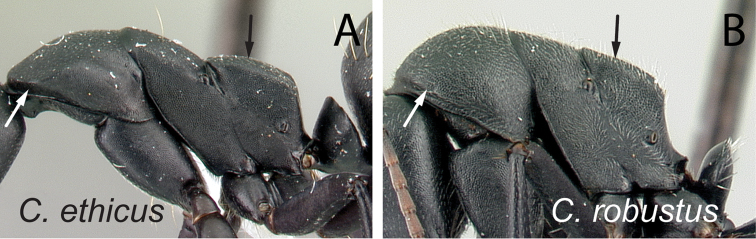
Lateral view of mesosoma. **A** *Camponotus ethicus* (CASENT0409949) **B** *Camponotus robustus* (CASENT0066723).

**Figure 8.**
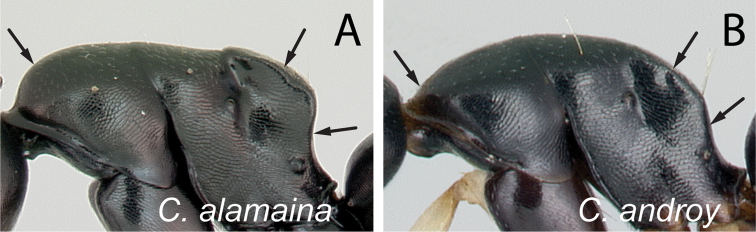
Mesosoma in lateral view. **A** *Camponotus alamaina* (CASENT0499291) **B** *Camponotus androy* (CASENT0453723).

**Figure 9.**
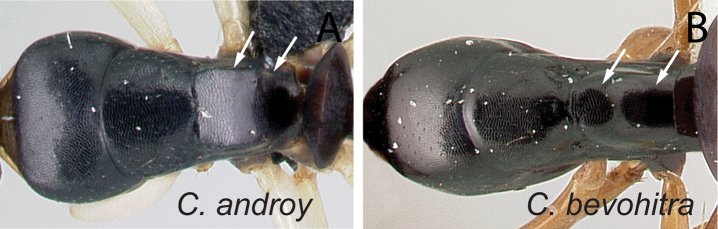
Mesosoma in dorsal view. **A** *Camponotus androy* (CASENT0453723) **B** *Camponotus bevohitra* (CASENT0437238).

**Figure 10.**
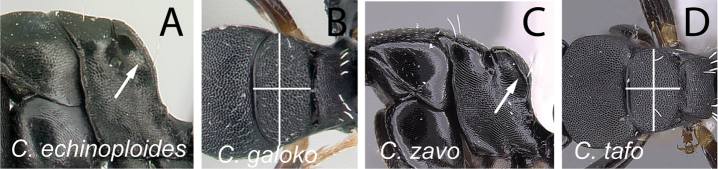
Mesosoma in lateral and dorsal views. **A** *Camponotus echinoploides* (CASENT0409171) and **B** *Camponotus galoko* (CASENT0178918) **C** *Camponotus zavo* (CASENT0060041) and **D** *Camponotus tafo* (CASENT0763608).

**Figure 11.**
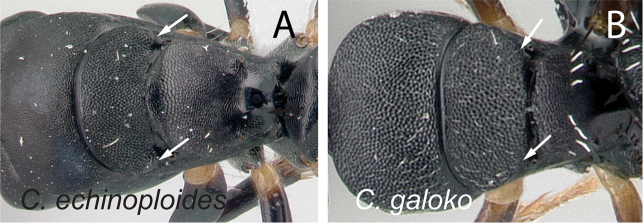
Mesosoma in dorsal view. **A** *Camponotus echinoploides* (CASENT0409171) **B** *Camponotus galoko* (CASENT0178918).

**Figure 12.**
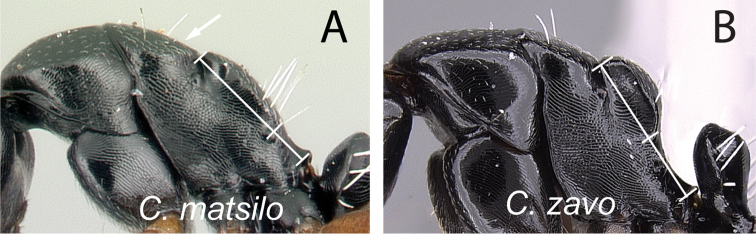
Mesosoma in lateral view. **A** *Camponotus matsilo* (CASENT0121843) **B** *Camponotus zavo* (CASENT0060041).

**Figure 13.**
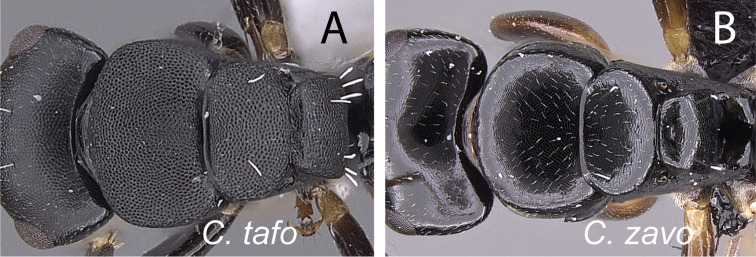
Head and mesosoma in dorsal view. **A** *Camponotus tafo* (CASENT0763608) **B** *Camponotus zavo* (CASENT0060041).

**Figure 14.**
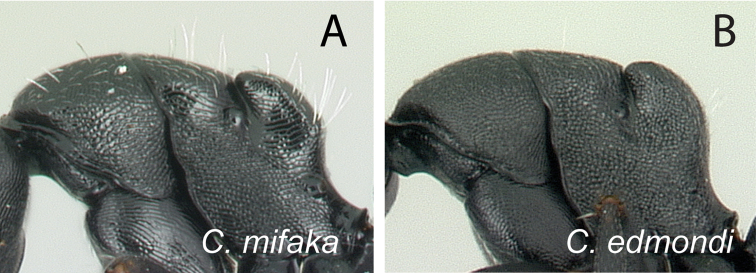
Mesosoma in lateral view. **A** *Camponotus mifaka* (CASENT0217301) **B** *Camponotus edmondi* (CASENT0136511).

**Figure 15.**
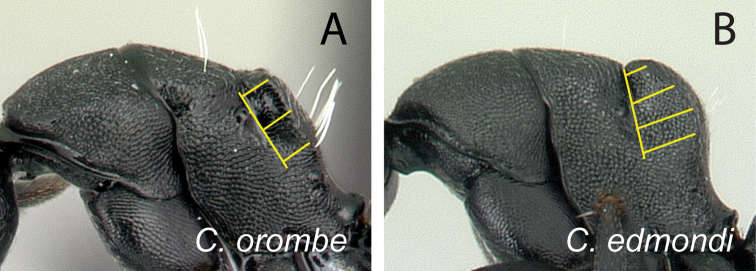
Mesosoma in lateral view. **A** *Camponotus orombe* (CASENT0178923) **B** *Camponotus edmondi* (CASENT0136511).

**Figure 16.**
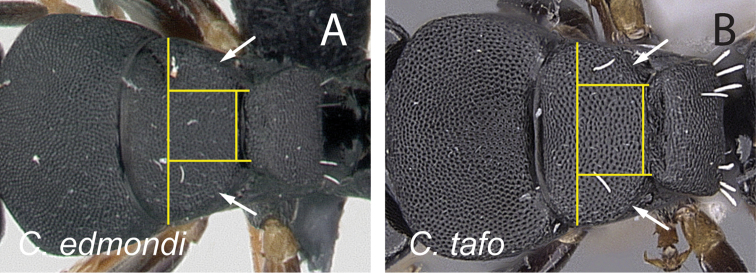
Mesosoma in dorsal view. **A** *Camponotus edmondi* (CASENT0134980) **B** *Camponotus tafo* (CASENT0763608).

**Figure 17.**
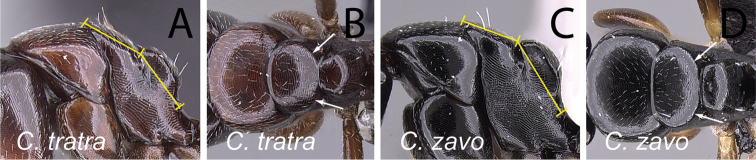
Mesosoma in profile and in dorsal view. **A, B** *Camponotus tratra* (CASENT0763608) **C, D** *Camponotus zavo* (CASENT0060041).

**Figure 18.**
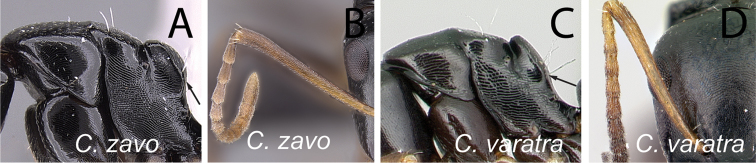
Mesosoma in lateral view and antennal scape in full-face view: **A, B** *Camponotus zavo* (CASENT0060041) **C** *Camponotus varatra* (CASENT0492888) and **D** *Camponotus varatra* (CASENT0409723).

### Species account of the Malagasy *Camponotus edmondi* species group

#### Camponotus alamaina

Taxon classificationAnimaliaHymenopteraFormicidae

Rakotonirina, Csősz & Fisher sp. n.

http://zoobank.org/CAEB7BC2-0095-4240-88EA-0019EAB8780B

**Figure 19.**
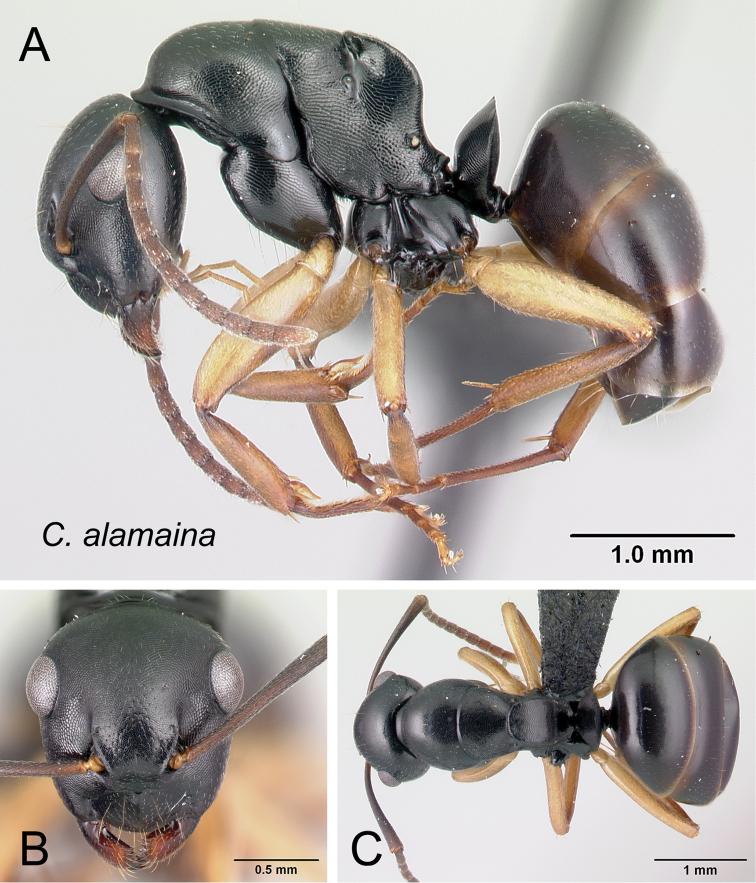
*Camponotus alamaina* minor worker CASENT0481799. **A** lateral view **B** head in full-face view **C** dorsal view.

Figures 5A , 6B , 8A , 19 , 34

##### Holotype worker.

Madagascar, Province Mahajanga, Mahavavy River, 6.2 km 145° SE Mitsinjo, –16.05167, 45.90833, 20 m, gallery forest, ex dead branch above ground, 1–5 Dec 2002 (Fisher, Griswold et al.) collection code: BLF06982, specimen code: CASENT0481799 (CASC).

##### Paratype.

8 workers with same data as holotype but with the following specimen codes: CASENT0481797, CASENT0481798, CASENT0746987, CASENT0746988, CASENT0746989, CASENT0763743, CASENT0763744, CASENT0763745 (BMNH, MHNG, MNHN, MSNG,CASC).

##### Additional material examined.

**Form 1. MADAGASCAR**: Province **Antananarivo**: Forêt de galerie, Telomirahavavy, 23.4 km NNE Ankazobe, –18.12167, 47.20627, 1520 m, disturbed gallery montane forest, (B.L. Fisher et al.) (CASC); Réserve Naturelle Sohisika, Sohisika 24.6 km NNE Ankazobe, –18.10322, 47.18692, 1464 m, gallery montane forest, (B.L. Fisher et al.) (CASC); Province **Antsiranana**: Forêt Ambato, 26.6 km 33° Ambanja, –13.4645, 48.55167, 150 m, rainforest, (B.L. Fisher) (CASC); Réserve Spéciale Manongarivo 17.3 km 218° SW Antanambao, –14.02167, 48.41833, 1580 m, montane rainforest, (B.L. Fisher) (CASC); Réserve Spéciale Manongarivo, 10.8 km 229° SW Antanambao, –13.96167, 48.43333, 400 m, rainforest, (B.L. Fisher) (CASC); Réserve Spéciale Ambre, 3.5 km 235° SW Sakaramy, –12.46889, 49.24217, 325 m, tropical dry forest, (Fisher, Griswold et al.) (CASC); Réserve Spéciale Ankarana, 13.6 km 192° SSW Anivorano Nord, –12.86361, 49.22583, 210 m, tropical dry forest, (Fisher, Griswold et al.) (CASC); Réserve Spéciale Ankarana, 22.9 km 224° SW Anivorano Nord, –12.90889, 49.10983, 80 m, tropical dry forest, (Alpert et al.), (Fisher, Griswold et al.) (CASC); Province **Fianarantsoa**: Southern Isoky-Vohimena Forest, –22.68333, 44.83333, 730 m, (Sylvain) (CASC); Forêt d’Analalava, 29.6 km 280° W Ranohira, –22.59167, 45.12833, 700 m, tropical dry forest, (Fisher, Griswold et al.) (CASC); Province **Mahajanga**: Forêt Ambohimanga, 26.1 km 314° Mampikony, –15.96267, 47.43817, 250 m, tropical dry forest, (B.L. Fisher) (CASC); Forêt d’Anabohazo, 21.6 km 247° WSW Maromandia, –14.30889, 47.91433, 120 m, tropical dry forest, (Fisher, Griswold et al.) (CASC); Forêt de Tsimembo, 11.0 km 346° NNW Soatana, –18.99528, 44.4435, 50 m, tropical dry forest, (Fisher-Griswold Arthropod Team) (CASC); Parc National Baie de Baly, 12.4 km 337° NNW Soalala, –16.01, 45.265, 10 m, tropical dry forest, (Fisher, Griswold et al.) (CASC); Mahavavy River, 6.2 km 145° SE Mitsinjo, –16.05167, 45.90833, 20 m, gallery forest, (Fisher, Griswold et al.) (CASC); Parc National Ankarafantsika, Forêt de Tsimaloto, 18.3 km 46° NE de Tsaramandroso, –16.22806, 47.14361, 135 m, tropical dry forest, (Fisher, Griswold et al.) (CASC); Parc National Namoroka, 16.9 km 317° NW Vilanandro, –16.40667, 45.31, 100 m, tropical dry forest, (Fisher, Griswold et al.) (CASC); Parc National Namoroka, 17.8 km 329° WNW Vilanandro, –16.37667, 45.32667, 100 m, tropical dry forest, (Fisher, Griswold et al.) (CASC); Parc National Namoroka, 9.8 km 300° WNW Vilanandro, –16.46667, 45.35, 140 m, tropical dry forest, (Fisher, Griswold et al.) (CASC); Réserve d’Ankoririka, 10.6 km 13° NE de Tsaramandroso, –16.26722, 47.04861, 210 m, tropical dry forest, (Fisher, Griswold et al.) (CASC); Réserve forestière Beanka, 50.2 km E Maintirano, –18.02649, 44.05051, 250 m, tropical dry forest on tsingy, (B.L. Fisher et al.) (CASC); Réserve forestière Beanka, 50.7 km E Maintirano, –17.88021, 44.46877, 140 m, tropical dry forest on tsingy, (B.L. Fisher et al.) (CASC); Réserve forestière Beanka, 52.7 km E Maintirano, –18.0622, 44.52587, 300 m, tropical dry forest on tsingy, (B.L. Fisher et al.) (CASC); Réserve forestière Beanka, 53.6 km E Maintirano, –18.04014, 44.53394, 272 m, tropical dry forest on tsingy, (B.L. Fisher et al.) (CASC); Parc National Tsingy de Bemaraha, 10.6 km ESE 123° Antsalova, –18.70944, 44.71817, 150 m, tropical dry forest on Tsingy, (Fisher-Griswold Arthropod Team) (CASC); Parc National Tsingy de Bemaraha, 2.5 km 62° ENE Bekopaka, Ankidrodroa River, –19.13222, 44.81467, 100 m, tropical dry forest on Tsingy, (Fisher-Griswold Arthropod Team) (CASC); Parc National Tsingy de Bemaraha, 3.4 km 93° E Bekopaka, Tombeau Vazimba, –19.14194, 44.828, 50 m, tropical dry forest, (Fisher-Griswold Arthropod Team) (CASC); Province **Toliara**: 50 km N Morondava, –20.06667, 44.58333, in primary dry forest, (A. Pauly) (CASC); 6.1 km 182° S Marovato, –25.58167, 45.295, 20 m, spiny forest/thicket, (Fisher-Griswold Arthropod Team) (CASC); Beza-Mahafaly, 27 km E Betioky, –23.65, 44.63333, 135 m, tropical dry forest, (B.L. Fisher) (CASC); Fiherenana, –23.17694, 43.96083, 100 m, gallery forest, (Frontier Project) (CASC); Fiherenana, –23.22252, 43.88088, 65 m, degraded gallery forest, (Frontier Project) (CASC); Forêt de Beroboka, 5.9 km 131° SE Ankidranoka, –22.23306, 43.36633, 80 m, tropical dry forest, (Fisher-Griswold Arthropod Team) (CASC); Forêt de Kirindy, 15.5 km 64° ENE Marofandilia, –20.06915, 44.66042, 30 m, tropical dry forest, (B.L. Fisher et al.) (CASC); Parc National Tsimanampetsotsa, Mitoho Cave, 6.4 km 77° ENE Efoetse, 17.4 km 170° S Beheloka, –24.04722, 43.75317, 40 m, spiny forest/thicket, (Fisher-Griswold Arthropod Team) (CASC); Forêt de Tsinjoriaky, 6.2 km 84° E Tsifota, –22.80222, 43.42067, 70 m, spiny forest/thicket, (Fisher-Griswold Arthropod Team) (CASC); Forêt Vohidava 88.9 km N Amboasary, –24.24067, 46.28783, 500 m, spiny forest/dry forest transition, (B.L. Fisher et al.) (CASC); Mikea Forest, deciduous dry forest, –22.90367, 43.4755, 30 m, deciduous dry forest, (R. Harin’Hala) (CASC); Mikea Forest, spiny forest, –22.91333, 43.48222, 37 m, spiny forest, (R. Harin’Hala) (CASC); Parc National Andohahela, Col du Sedro, 3.8 km 113° ESE Mahamavo, 37.6 km 341° NNW Tolagnaro, –24.76389, 46.75167, 900 m, montane rainforest, (Fisher-Griswold Arthropod Team) (CASC); Parc National Zombitse, 17.7 km 98° E Sakaraha, –22.88833, 44.70167, 760 m, tropical dry forest, (Fisher, Griswold et al.) (CASC); Parc National Zombitse, 19.8 km 84° E Sakaraha, –22.84333, 44.71, 770 m, tropical dry forest, (Fisher, Griswold et al.) (CASC); Réserve Privée Berenty, Forêt de Bealoka, Mandraré River, 14.6 km 329° NNW Amboasary, –24.95694, 46.2715, 35 m, gallery forest, (Fisher-Griswold Arthropod Team) (CASC); Réserve Privée Berenty, Forêt de Malaza, Mandraré River, 8.6 km 314° NW Amboasary, –25.00778, 46.306, 40 m, gallery forest, (Fisher-Griswold Arthropod Team) (CASC); Vohibasia Forest, 59 km NE Sakaraha, –22.46667, 44.85, 780 m, (Sylvain) (CASC).

**Form 2. MADAGASCAR**: Province **Mahajanga**: Parc National Baie de Baly, 12.4 km 337° NNW Soalala, –16.01, 45.265, 10 m, tropical dry forest, (Fisher, Griswold et al.) (CASC); Réserve Spéciale Bemarivo, 23.8 km 223° SW Besalampy, –16.925, 44.36833, 30 m, tropical dry forest, (Fisher, Griswold et al.) (CASC); Province **Toliara**: Forêt de Kirindy, 15.5 km 64° ENE Marofandilia, –20.045, 44.66222, 100 m, tropical dry forest, (Fisher-Griswold Arthropod Team) (CASC).

**Form 3. MADAGASCAR**: Province **Antsiranana**: Forêt d’Ampondrabe, 26.3 km 10° NNE Daraina, –12.97, 49.7, 175 m, tropical dry forest, (B.L. Fisher) (CASC); Forêt d’Analabe, 30.0 km 72° ENE Daraina, –13.08333, 49.90833, 30 m, littoral rainforest, (B.L. Fisher) (CASC); Forêt de Bekaraoka, 6.8 km 60° ENE Daraina, –13.16667, 49.71, 150 m, tropical dry forest, (B.L. Fisher) (CASC); Forêt de Binara, 7.5 km 230° SW Daraina, –13.255, 49.61667, 375 m, tropical dry forest, (B.L. Fisher) (CASC); Forêt d’Orangea, 3.6 km 128° SE Remena, –12.25889, 49.37467, 90 m, littoral rainforest, (Fisher, Griswold et al.) (CASC); Montagne des Français, 7.2 km 142° SE Antsiranana (=Diego Suarez), –12.32278, 49.33817, 180 m, tropical dry forest, (Fisher, Griswold et al.) (Alpert et al.) (CASC); Réserve Analamerana, 16.7 km 123° Anivorano-Nord, –12.80467, 49.37383, 225 m, tropical dry forest, (B.L. Fisher) (CASC); Réserve Analamerana, 28.4 km 99° Anivorano-Nord, –12.74667, 49.49483, 60 m, tropical dry forest, (B.L. Fisher) (CASC); Réserve Spéciale de l’Ankarana, 13.6 km 192° SSW Anivorano Nord, –12.86361, 49.22583, 210 m, tropical dry forest, (Fisher, Griswold et al.) (CASC); Réserve Spéciale Ankarana, 22.9 km 224° SW Anivorano Nord, –12.90889, 49.10983, 80 m, tropical dry forest, (Fisher, Griswold et al.) (CASC).

##### Diagnosis.

Anterior margin of petiolar node convex and posterior margin more or less straight; propodeal spiracle anterior to posterolateral margin of propodeum; head and mesosoma black to dark brown, gaster and appendages dark brown to yellow or light yellow; anterior margin of pronotum broadly rounding to the dorsum; dorsolateral and posterolateral margins of propodeum strongly carinate.

##### Description.

**Minor worker** (Figs 5A, 6B, 8A, 19). In full-face view head longer than broad (CWb/CL: 0.79–0.91); lateral margin more or less straight, feebly converging toward base of mandibles and broadly rounding to the convex posterior margin. Anterior clypeal margin generally convex, posteromedian margin notched. Level of posterior ocular borders generally located from posterior third to posterior fifth of head (PoOc/CL: 0.2–0.271); antennal scape somewhat long (SL/CS: 1.02–1.18), roughly its distal portion extending beyond rear border of head. Mandible subtriangular, apical margin armed with six teeth. In profile, anterior margin of pronotum broadly rounding to the dorsum; anterodorsal angle weakly marginate; junction of dorsum and sides of premesonotum rounded, without margination; dorsolateral margins of propodeum extending into sharp carina. In dorsal view, junction of mesonotum and propodeum laterally compressed; metanotal groove impressed. In profile, propodeal dorsum raised into a very short edge, descending feebly posteriorly and joining declivity by distinct angle; propodeal spiracle located anterior to posterolateral margin of propodeum. Maximum width of procoxa larger than width of meso-metapleuron. In profile, petiolar node anteroposteriorly flattened and tapered dorsally; anterior margin slightly convex and posterior margin more or less straight; dorsal margin straight or weakly excised medially. Constriction between abdominal segments III and IV lacking.

Dorsum of head, mesosoma, and petiole with imbricate sculpture; gaster with finer imbrication; mandible coriarious-puncticulate. Pairs of erect hairs arranged as follows: three on clypeus, one near margin of frontal carina, at level of eyes, posterior portion of head, dorsum of mesonotum and propodeum. Two rows of sparse, erect hairs arranged on anterior and posterior portions of each of first three gastral tergites. Pubescence short and scattered on dorsum of body. Head, mesosoma and petiole black to dark brown; gaster, mandible, antenna, coxa and tarsus brown; rest of legs yellow.

**Major worker.** With characteristics of minor worker except the following divergent features: head subquadrate (CWb/CL: 0.87–1) in full-face view, posterior margin approximately straight; level of posterior ocular borders at about posterior fourth of head (PoOc/CL: 0.24–0.28); anterior clypeal margin straight and medially excised; antennal scape barely extending beyond rear cephalic border (SL/CS: 0.69–0.89); metanotum distinct between mesonotum and propodeum; dorsum of propodeum strongly inclined posteriorly and rounding into declivity; dorsal margin of petiolar node medially excised; few erect hairs present on dorsum of pronotum and more than one pair on mesonotum and propodeum.

##### Distribution and biology.

*Camponotus alamaina* is a widespread species occurring mainly in the dry forest habitats in western Madagascar (Fig. 34). Members of the species are known also from the spiny forest and thickets of the south and southwest, the montane rainforest of the central plateau and the southeast, and the littoral forest of the north of the island. Although this species is both arboreal and terrestrial, its members commonly are found foraging on low vegetation and nesting in dead branches, twigs, or rot pockets above the ground. Nests sites also may be built in rotten logs or sticks, and rotting tree stumps.

Variant 1 (typical form) and variant 2 co-occur in the dry forests of the Réserve de Bemarivo and Parc National Baie de Baly.

##### Discussion.

*Camponotus alamaina* is one of the common species in the *edmondi* group and displays remarkable morphological variation in the shape of the propodeum, form of the petiolar node, and color of the legs. Three different variants are recognized based on this morphological diversity, but gradually merge into one another across the geographic distribution of the species.

**Variant 1.** Workers are typical *Camponotus alamaina* and can be recognized by having dorsolateral and posterolateral margins of the propodeum that extend into sharp carinae, but the junction of the dorsum to the posterolateral portion is rounded or bluntly angulate and does not form a pair of teeth or tubercles laterally; in oblique profile view, the dorsal border of petiolar node is straight or slightly excised medially; and the legs are yellow.

**Variant 2.** This variant is known from Parc National Baie de Baly, Réserve de Bemarivo and Kirindy Forest near Marofandilia, and is characterized by the lateral projection into tubercles of the posterodorsal corner of the propodeum, the presence of numerous erect hairs on the dorsum of the propodeum, a much thicker petiolar node with a dorsal margin extending medially into a blunt tooth in frontal view, and a much darker-colored foreleg.

**Variant 3.** This variant expresses intermediate characters of the previous two variants, in that the posterodorsal corners of the propodeum are bidentate, the dorsal margin of petiolar node is slightly excised medially in frontal view, and the legs are yellow. Specimens of this variant have been collected from sites in the north of Madagascar, including Ankarana, Orangea, Montagne des Français, Binara, and Analabe.

The NC-clustering approach was used to detect these three variants, but the technique did not clearly reveal their existence. The members of each of the variants are scattered along the cluster of *Camponotus alamaina*. More information from the robust molecular phylogenetics are needed in order both to decide whether the different variants constitute separate species and to study the ecological and evolutionary forces underlying these morphological variations.

#### Camponotus androy

Taxon classificationAnimaliaHymenopteraFormicidae

Rakotonirina, Csősz & Fisher sp. n.

http://zoobank.org/0922743A-9E3D-4E33-A948-EB27C8FC9235

**Figure 20.**
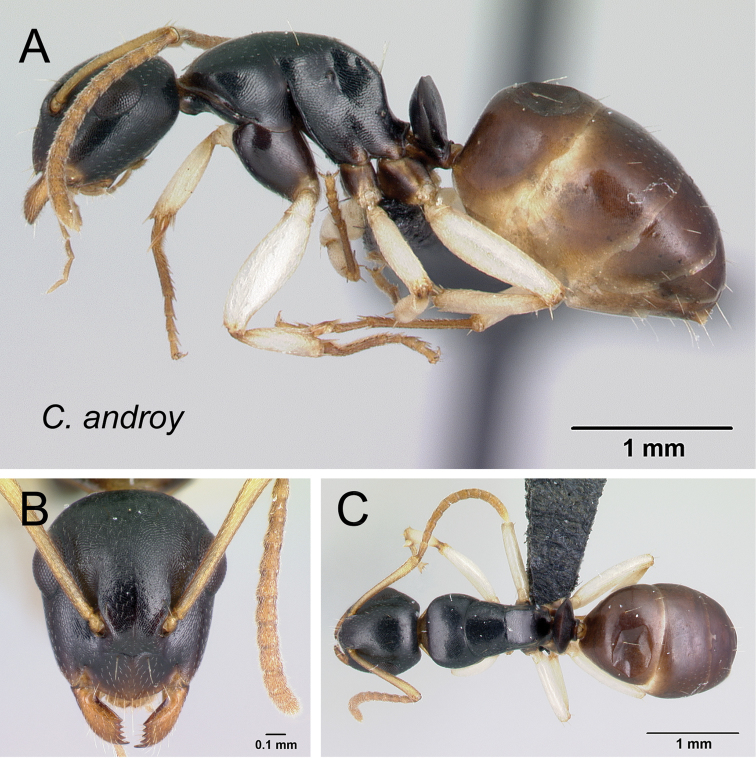
*Camponotus androy* minor worker CASENT0453723. **A** lateral view **B** head in full-face view **C** dorsal view.

Figures 8B , 9A , 20 , 35

##### Holotype worker.

Madagascar, Province Toliara, Réserve Spéciale de Cap Sainte Marie, 12.3 km 262° W Marovato, –25.58167, 45.16833, 200 m, spiny forest/thicket, ex dead twig above ground, 11–15 Fev 2002 (Fisher-Griswold Arthropod Team) collection code: BLF05583 specimen code: CASENT0453723 (CASC).

##### Paratype.

16 workers with same data as holotype but with the following specimen codes: CASENT0453722, CASENT0453725, CASENT0453726, CASENT0453727, CASENT0453728, CASENT0453729, CASENT0453730, CASENT0453731, CASENT0453732, CASENT0453734, CASENT0746981, CASENT0746982, CASENT0746983, CASENT0746984, CASENT0746985, CASENT0746986 (BMNH, MHNG, MNHN, MSNG, CASC).

##### Additional material examined.

**MADAGASCAR**: Province **Toliara**: 3.4 km 190° S Marovato, –25.55972, 45.2825, 160 m, spiny forest/thicket, (Fisher-Griswold Arthropod Team) CASC); Réserve Spéciale Cap Sainte Marie, 12.3 km 262° W Marovato, –25.58167, 45.16833, 200 m, spiny forest/thicket, (Fisher-Griswold Arthropod Team) (CASC); Réserve Spéciale Cap Sainte Marie, 12.3 km 262° W Marovato, –25.58167, 45.16833, 200 m, spiny forest/thicket, (Fisher-Griswold Arthropod Team) (CASC).

##### Diagnosis.

Anterior margin of petiolar node convex and posterior margin more or less straight; propodeal spiracle placed anterior to posterolateral margin of propodeum; head and mesosoma black to dark brown, gaster and appendages dark brown to yellow or depigmented yellow; cervical shield joining pronotal dorsum directly; dorsolateral portion of propodeum with sharp carina, posterolateral margin marginate; in profile, width of mesopleuron about as large as that of lateral portion of propodeum; at least a pair of erect hairs present on propodeal dorsum.

##### Description.

**Minor worker** (Figs 8B, 9A, 20). In full-face view head elongate (CWb/CL: 0.78–0.83), sides almost straight and rounding to the broadly convex posterior margin. Eyes protruding and large (EL/CS: 0.34–0.38), occupying more than one third of the side of the head; level of posterior ocular margin at posterior fifth portion of head (PoOc/CL: 0.19–0.22). Anteromedian margin of clypeus with blunt angle; posterior margin slightly notched medially. Mandible subtriangular, equipped with six teeth. Antennal scape relatively long, apical third portion surpassing rear cephalic margin (SL/CS: 0.97–1.1). Anterior face of pronotum very short, cervical shield directly joining pronotal dorsum. Anterodorsal angle and anterior portion of dorsolateral junction of pronotum marginate. In dorsal view, mesonotum almost as long as broad, width narrowing posteriorly; metanotal groove slightly impressed near sides and vestigial medially. In lateral view, width of mesopleuron, as seen at spiracle level, about as large as width of lateral portion of propodeum. Dorsolateral margin of propodeum extended into sharp carina; sides of propodeum and declivitous surface separated by sharp margination; propodeal dorsum sloping posteriorly and joining declivitous margin by blunt or rounded angle; propodeal spiracle on lateral portion of propodeum, located anterior to posterolateral margin of propodeum. Procoxa as wide as meso-metapleuron. In profile, petiolar node anteroposteriorly compressed; anterior margin convex and posterior margin more or less straight; dorsal margin medially excised. Constriction between abdominal segments III and IV absent.

Head, mesosoma, and petiolar node shining with imbricating sculpture; gastral tergite finely imbricate. Mandible with sparse piligerous punctures between smooth and shining surface. Number of pairs of erect hairs arranged as follows: three on clypeus, one near margins of frontal carinae, one at level of eyes, and one close to posterior margin of head; none on pronotum; one on mesonotum and propodeum at junction of dorsum and declivity; none on petiole. Two erect hairs on dorsum of anterior portion of first gastral tergite and four erect hairs on anterior and posterior portion of dorsum of two following gastral tergites. Pubescence sparse and short. Head and mesosoma black to dark brown; coxa, petiolar node and gaster brown to light brown; appendages proximally whitish-yellow (depigmented yellow) and apically light brown to yellow.

**Major worker.** With characteristics of minor worker except the following divergent features: posterior margin of head straight; lateral margin straight posteriorly and convex from anterior level of eyes to base of mandible; mandible robust with strong concavity near base of lateroventral portion and armed with eight teeth; level of posterior ocular margin at posterior fourth portion of head (PoOc/CL: 0.24–0.26); antennal scape reaching posterior cephalic border (SL/CS: 0.67–0.71); anterior portion of head with scattered shallow punctures. Pair of erect hairs arranged as: one or two on pronotum and propodeum, one on mesonotum.

##### Distribution and biology.

*Camponotus androy* is restricted to the spiny bush and thicket of Marovato region and the Réserve Spéciale Sainte Marie in the extreme south portion of Madagascar (Fig. 35). Across these areas, between 20 m and 200 m of altitude, colony nests have been generally established in dead twigs above the ground and rarely in rotten logs or dead tree stumps.

##### Discussion.

Workers of *Camponotus androy* might be confused with those of *Camponotus bevohitra* in that they have a more or less straight posterior margin of the petiolar node, a very short anterior margin of pronotum and slightly carinate posterolateral margin of the propodeum. However, *Camponotus androy* is characterized by a narrower mesopleuron, which is about as large as the width of the lateral portion of the propodeum; at least one pair of erect hairs is present on the propodeal dorsum. In *Camponotus bevohitra*, the mesopleural width, taken at the level of the metanotal spiracle, is much larger than the width of the lateral portion of propodeum; the propodeal dorsum lacks erect hairs.

The taxonomic decision for *Camponotus androy* based on qualitative morphology-based study is corroborated by multivariate morphometric analysis. This species is classified correctly by confirmatory LDA at 100% success.

#### Camponotus bevohitra

Taxon classificationAnimaliaHymenopteraFormicidae

Rakotonirina, Csősz & Fisher sp. n.

http://zoobank.org/6F93F122-9030-4D7F-B7A0-71ECA6CC88FF

**Figure 21.**
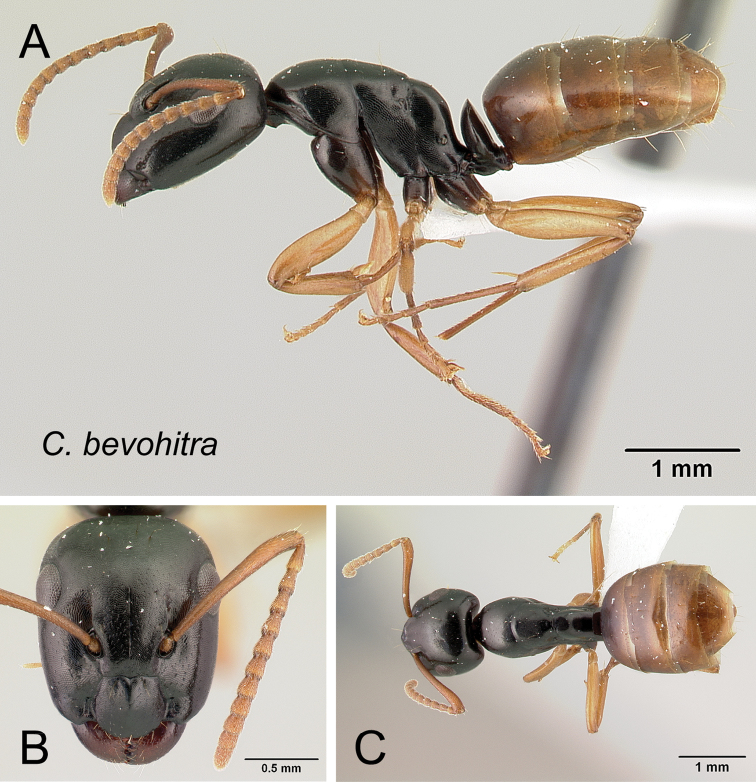
*Camponotus bevohitra* major worker CASENT0437238. **A** lateral view; B head in full-face view **C** dorsal view.

Figures 9B , 21 , 36

##### Holotype worker.

Madagascar, Province Antananarivo, Réserve Spéciale d’Ambohitantely, Forêt d Ambohitantely, 20.9 km 72° NE d Ankazobe, –18.22528, 47.28683, 1410 m, montane rainforest, ex dead twig above ground, 17–22 Apr 2001 (Fisher, Griswold et al.) collection code: BLF03727 specimen code: CASENT0437247 (CASC).

**Paratype.** 10 workers with same data as holotype but with the following specimen codes: CASENT0437237, CASENT0437238, CASENT0437239, CASENT0437240, CASENT0437241, CASENT0437243, CASENT0437244, CASENT0437245, CASENT0437246, CASENT0437248, (BMNH, MHNG, MNHN, MSNG, CASC).

##### Additional material examined.

**MADAGASCAR**: Province **Antananarivo**: Forêt de galerie, Andranorovitra, 24.0 km NNE Ankazobe, –18.11243, 47.19757, 1491 m, disturbed gallery montane forest, (B.L. Fisher et al.) (CASC); Réserve Naturelle Sohisika, Sohisika 24.6 km NNE Ankazobe, –18.10322, 47.18692, 1464 m, gallery montane forest, (B.L. Fisher et al.) (CASC); Réserve Spéciale Ambohitantely, Forêt d Ambohitantely, 20.9 km 72° NE d Ankazobe, –18.22528, 47.28683, 1410 m, montane rainforest, (Fisher, Griswold et al.) (CASC); Province **Fianarantsoa**: Forêt d’Atsirakambiaty, 7.6 km 285° WNW Itremo, –20.59333, 46.56333, 1550 m, montane rainforest (Fisher, Griswold et al.) (CASC).

##### Diagnosis.

Anterior margin of petiolar node convex and posterior margin more or less straight; propodeal spiracle placed anterior to posterolateral margin of propodeum; head and mesosoma black to dark brown, gaster and appendages dark brown to yellow or depigmented yellow; cervical shield joining pronotal dorsum directly; junction of dorsal face to lateral face of propodeum without sharp carina, posterolateral margin rounded; in profile mesopleuron much wider than lateral portion of propodeum; erect hairs lacking on propodeal dorsum.

##### Description.

**Minor worker** (Fig. 9B). In full-face view head weakly longer than broad (CWb/CL: 0.78–0.85), posterior margin broadly convex; sides almost straight, their junction to posterior border concealed by eyes. Eyes large (EL/CS: 0.27–0.32) and strongly protruding, occupying roughly one third the side of head; level of posterior ocular margin at posterior fifth portion of head (PoOc/CL: 0.18–0.21). Anteromedian margin of clypeus straight; posteromedian margin slightly notched. Mandible subtriangular, armed with six teeth. Antennal scape relatively long (SL/CS: 0.905–1.109), more than apical third portion extending beyond posterior cephalic border. Promesonotal dorsum flattened; anterodorsal angle of pronotum and dorsolateral portion of mesosoma bluntly marginate; posterolateral margin of propodeum rounding to declivitous surface. In dorsal view, mesonotum as long as broad, but sides converging posteriorly; mesonotum and propodeum laterally compressed at their junction; metanotal groove vestigial, represented by a transverse line. In lateral view, width of mesopleuron at the level of spiracle much wider than lateral portion of propodeum. Propodeal dorsum inclined posteriorly and rounding to declivitous margin; propodeal spiracle located anterior to posterolateral border of propodeum. Maximum width of coxa of foreleg as broad as meso-metapleuron width. In lateral view, petiolar node scale-like; anterior margin slightly convex and posterior margin more or less straight; dorsal margin straight or medially angulate. Junction between abdominal segments III and IV without constriction.

Dorsum of head, mesosoma shining with imbricate sculpture; gaster finely imbricate. Mandible coriarious-puncticulate. Few erect hairs on clypeus and gastral tergites; one pair each near margin of frontal carina, on posterior cephalic portion, and on mesonotum. Erect hairs lacking from pronotum, propodeum, and petiolar node. Pubescence very scarce. Head, mesosoma, and petiole black to dark brown; gaster, mandible, antenna, coxa, and tarsus dark brown to brown; remainder of legs yellow.

**Major worker** (Fig. 21). With characteristics of minor worker with the exception of the following features: posterior margin of head straight and rounding to lateral margins; apical sixth portion of antennal scape surpassing posterior cephalic border (SL/CS: 0.753–0.852); metanotum visible between mesonotum and propodeum; sparse shallow punctures present laterally on head from level of anterior ocular margins and clypeus to base of mandible.

##### Distribution and biology.

The distribution of *Camponotus bevohitra* is limited to montane rainforest habitats in the central high plateau of Madagascar (Fig. 36). Specimens have been collected foraging on lower vegetation. The species nests in dead twigs or branches above the ground.

##### Discussion.

See discussion of species differentiation under *Camponotus androy*. In the present study, the definition of *Camponotus bevohitra* based on both qualitative morphological analysis and morphometrics is congruent. The grouping shown by the morphometric dendrogram and confirmed by cumulative LDA at 100% success supports the existence of the species.

#### Camponotus echinoploides

Taxon classificationAnimaliaHymenopteraFormicidae

Forel

**Figure 22.**
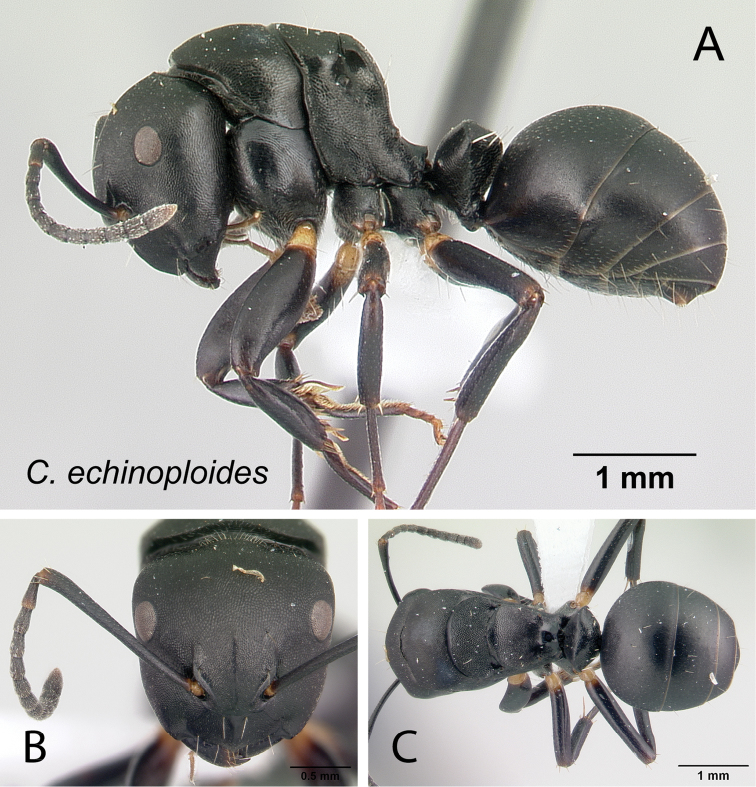
*Camponotus echinoploides* minor worker CASENT0409171. **A** lateral view **B** head in full-face view **C** dorsal view.

Figures 10A , 11A , 22 , 37

Camponotus echinoploides Forel, 1891: 51. Holotype minor worker, Madagascar, 30 miles northwest Toamasina (=Tamatave) (O’swald), AntWeb CASENT0101379 (MHNG) [examined]. [Combination in Camponotus (Myrmobrachys): Forel 1914: 270; in Camponotus (Orthonotomyrmex): Emery 1920: 258; in Camponotus (Myrmepinotus): Santschi 1921: 312; Wheeler 1922: 1053; Emery 1925: 126; Bolton 1995: 97, 131].

##### Additional material examined.

**MADAGASCAR**: Province **Antananarivo**: 3 km 41° NE Andranomay, 11.5 km 147° SSE Anjozorobe, –18.47333, 47.96, 1300 m, montane rainforest (Fisher, Griswold et al.) (CASC); Province **Antsiranana**: Réserve Spéciale Manongarivo, 14.5 km 220° SW Antanambao, –13.99833, 48.42833, 1175 m, montane rainforest, (B.L. Fisher) (CASC); Réserve Spéciale Manongarivo, 10.8 km 229° SW Antanambao, –13.96167, 48.43333, 400 m, rainforest (B.L. Fisher) (CASC); Parc National de Marojejy, Manantenina River, 27.6 km 35° NE Andapa, 9.6 km 327° NNW Manantenina, –14.435, 49.76, 775 m, rainforest (B.L. Fisher) (CASC); Rés. Analamerana, 16.7 km 123° Anivorano-Nord, –12.80467, 49.37383, 225 m, tropical dry forest (B.L.Fisher) (CASC); Betaolana Forest, along Bekona River, –14.52996, 49.44039, 880 m, rainforest, (B.L. Fisher et al.) (CASC); Province **Fianarantsoa**: Parc National Befotaka-Midongy, Papango 27.7km S Midongy-Sud, Mount Papango, –23.83517, 46.96367, 940 m , rainforest (B.L. Fisher et al.) (CASC); radio tower, Ranomafana National Park, –21.25083, 47.40717, 1130 m, forest edge, mixed tropical forest, open area (M. Irwin, R. Harin’Hala) (CASC); JIRAMA water works near river, Ranomafana National Park, –21.2485, 47.45217, 690 m, open area near stream, (R. Harin’Hala) (CASC); Province **Mahajanga**: Réserve Spéciale Marotandrano, Marotandrano 48.3 km S Mandritsara, –16.28322, 48.81443, 865 m, transition humid forest, (B.L. Fisher et al.) (CASC); Province **Toamasina**: Montagne d’Anjanaharibe, 18.0 km 21° NNE Ambinanitelo, –15.18833, 49.615, 470 m, rainforest, (Fisher, Griswold et al.) (CASC); Montagne d’Akirindro 7.6 km 341° NNW Ambinanitelo, –15.28833, 49.54833, 600 m, rainforest (Fisher, Griswold et al.) (CASC); Parc National Mananara-Nord, 7.1 km 261° Antanambe, –16.455, 49.7875, 225 m, rainforest, (B.L. Fisher et al.) (CASC); Corridor Forestier Analamay-Mantadia, Ambatoharanana, –18.80388, 48.40506, 1013 m, rainforest, (B.L. Fisher et al.) (CASC); Province **Toliara**: Forêt Classée Analavelona, 29.2 km 343° NNW Mahaboboka, –22.675, 44.19, 1100 m, montane rainforest (Fisher, Griswold et al.) (CASC).

##### Diagnosis.

In profile, anterior and posterior margins of petiolar node convex; in profile, propodeum strongly compressed anteroposteriorly, without clear distinction between dorsal margin and declivity; in dorsal view, mesonotum three times as broad as long; posterodorsal corner of mesonotum raised into a bluntly rounded shield.

##### Description.

**Minor worker** (Figs 10A, 11A, 22). In full-face view head as long as broad (CWb/CL: 0.94–1), broader posteriorly; posterior margin broadly convex, lateral margins roughly straight. Eyes larger relative to head size (EL/CS: 0.19–0.24), anterior level located at about posterior third of head (PrOc/CL: 0.52–0.6). Clypeus with broadly convex anterior margin and medially notched posterior margin. Mandible triangular, masticatory margin armed with six sharp teeth. Antennal scape slightly long (SL/CS: 0.9–1.05), distal half almost surpassing posterior cephalic margin. Pronotal dorsum flat, anteriorly projecting into narrow ridge; dorsum and lateral face separated by longitudinal margination. In dorsal view, mesonotum three times as broad as long, posterodorsal corner raised into bluntly rounded ridge. Propodeum strongly compressed anteroposteriorly and lacking a clear separation of the dorsal margin and declivity. Propodeal spiracle located on posterior face of propodeum. Procoxa voluminous, maximum width as large as the combined width of meso-metapleuron and propodeal surface; femur of foreleg enlarged, twice as large as those of mid-leg and hind leg. Anterior and posterior margins of petiolar node convex. No constriction between abdominal segments III and IV.

Dorsum of head and mesosoma finely and densely reticulate punctate. Mandible finely and densely reticulate superimposed with scattered large punctures. Gastral segments covered with finely and densely reticulate punctate sculpture. Whitish erect hairs present as a pair on mesonotum and as two pairs near lateral margins of propodeum. Hairs lacking on pronotum. Whitish erect hairs present near lateral margins of posterior face of petiolar node. Gastral segments with sparse and much shorter erect hairs and pubescence. Body color shining black; appendages black to dark brown basally, flagellum brown, trochanter and metatarsus light brown to yellow.

**Major worker.** Characteristics of minor worker, except: head in full-face view as long as broad (CWb/CL: 1.01–1.02), lateral margins almost parallel, but strongly converging near the base of mandibles. Eyes smaller relative to head size (EL/CS: 0.17), anterior level located roughly at mid-length of head (PrOc/CL: 0.51–0.51). Anterior clypeal margin truncate and posterior margin medially notched. Apical portion of antennal scape barely reaching posterior cephalic margin (SL/CS: 0.6–0.62). In dorsal view, mesonotum roughly twice as broad as long. Metanotum visible between metanotal groove and propodeum.

##### Distribution and biology.

Endemic to Madagascar, *Camponotus echinoploides* occupies the eastern rainforest (Fig. 37), areas with transitional northern rainforest, relict montane rainforest in the central plateau, and the southwest of the island. The fact that most of its members have been found foraging on low vegetation and nesting in dead branches above the ground suggests that *Camponotus echinoploides* is arboreal.

##### Discussion.

*Camponotus echinoploides* is mostly similar to *Camponotus galoko*, but the latter has no extended shield rising from the posterodorsal corner of the mesonotum. The conventional taxonomic delimitation of *Camponotus echinoploides* is confirmed by the NC-clustering method. In addition, the recognition of the species is corroborated by confirmatory LDA at 100% classification success.

#### Camponotus edmondi

Taxon classificationAnimaliaHymenopteraFormicidae

André

**Figure 23.**
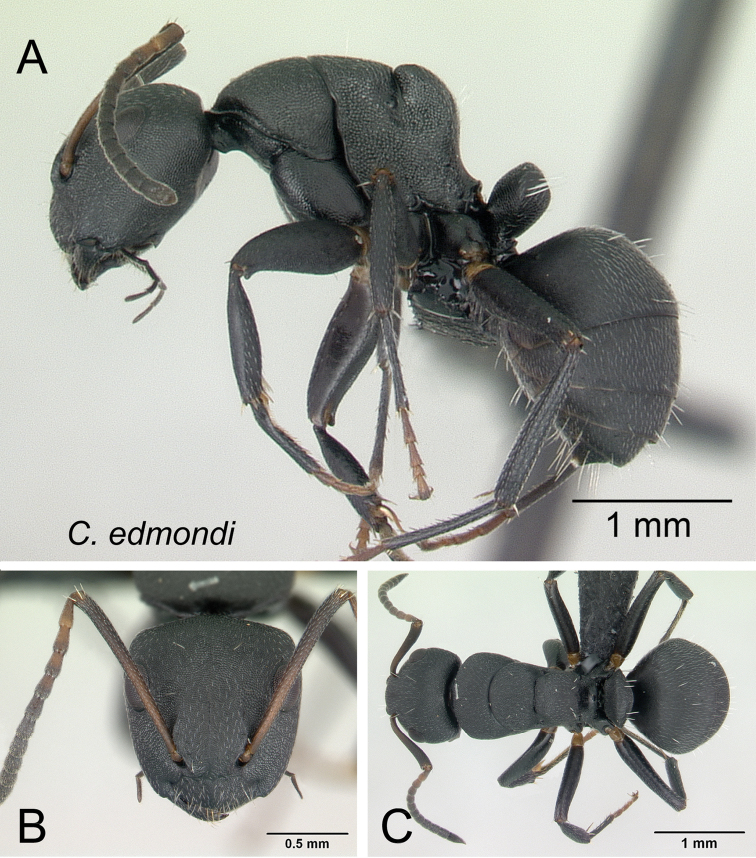
*Camponotus edmondi* minor worker CASENT0136511. **A** lateral view **B** head in full-face view **C** dorsal view.

Figures 14B , 15B , 16A , 23 , 38

Camponotus edmondi André, 1887: 281. Lectotype minor worker, **present designation**, Toamasina (=Tamatave), Madagascar (E. André), AntWeb CASENT0101384 (MHNG) [examined]. [Combination in Camponotus (Myrmobrachys): Forel 1914: 270; in Camponotus (Orthonotomyrmex): Emery 1920: 258; Wheeler 1922: 1049; in Camponotus (Myrmisolepis): Santschi 1921: 310. in Camponotus (Myrmepinotus): Emery 1925: 127; Bolton 1995: 97, 131].
Camponotus edmondi var. ernesti Forel, 1891: 50. Syntype major worker, Madagascar, Toamasina Province, 30 miles northwest of Toamasina (=Tamatave) (O’Swald) [not examined]. [Combination in Camponotus (Orthonotomyrmex): Wheeler 1922: 1049; in Camponotus (Myrmepinotus): Emery 1925: 127; Bolton 1995: 97]. **Syn. n.**

##### Additional material examined.

**MADAGASCAR**: Province **Antsiranana**: Forêt Ambanitaza, 26.1 km 347° Antalaha, –14.67933, 50.18367, 240 m, rainforest, (B.L. Fisher) (CASC); Forêt Ambanitaza, 26.1 km 347° Antalaha, –14.67933, 50.18367, 240 m, rainforest, (B.L. Fisher) (CASC); Vohemar, –13.37723, 50.0205, 25 m, cultivated land, (B.L. Fisher et al.) (CASC); Province **Fianarantsoa**: Manakara, –22.14817, 48.02267, 10 m, urban gardens, coastal *Casuarina equisetifolia*, (B.L. Fisher et al.) (CASC); Province **Toamasina**: Antongil Bay (Mocquerys) (MSNG); Brickaville, –18.82183, 49.07017, 24 m, urban/garden, (B.L. Fisher et al.) (CASC); Sainte Marie (MNHN); Ile Sainte Marie, Forêt Ambohidena, 22.8 km 44° Ambodifotatra, –16.82433, 49.96417, 20 m, littoral rainforest, (B.L. Fisher et al.) (CASC); Parcelle E3 Tampolo, –17.28104, 49.43012, 10 m, littoral forest, (Malagasy ant team) (CASC); Station Forestière Tampolo, 10 km NNE Fenoarivo Atsinanana, –17.2825, 49.43, 10 m, littoral rainforest, (B.L. Fisher) (CASC); Reserve Betampona, Camp Vohitsivalana, 37.1 km 338° Toamasina, –17.88667, 49.2025, 520 m, rainforest, (B.L. Fisher et al.) (CASC); 11 km SE Ampasimanolotra, Brickaville, –18.9, 49.13333, 5 m, littoral rainforest, (P.S. Ward) (PSWC); Nosy Mangabe, –15.5, 49.76667, 5m, littoral vegetation, (P.S. Ward) (PSWC); Province **Toliara**: 2.7 km WNW 302° Ste. Luce, –24.77167, 47.17167, 20 m, littoral rainforest, (B.L. Fisher) (CASC); Libanona beach, Tolagnaro, –25.03883, 46.996, 20 m, coastal scrub, (B.L. Fisher et al.) (CASC). **COMOROS: Anjouan**: Mount Ntringui, –12.19865, 44.41866, 740 m, montane forest, (B.L. Fisher et al.) (CASC); –12.18771, 44.35929, 65 m, coastal roadside, (B.L. Fisher et al.) (CASC); –12.18771, 44.35929, 65 m, coastal roadside, (B.L. Fisher et al.) (CASC); –12.30537, 44.45031, 500 m, along roadside, mango, banana, (B.L. Fisher et al.) (CASC); **Grande Comore**: Mouadja, –11.47435, 43.3004, 350 m, coastal scrub, (B.L. Fisher et al.) (CASC); Itoundzou, –11.63136, 43.30434, 635 m, secondary rainforest along roadside, (B.L. Fisher et al.) (CASC); Pidjani, –11.75447, 43.45148, 35 m, coastal scrub, (B.L. Fisher et al.) (CASC); Mouadja, –11.47435, 43.3004, 350 m, coastal scrub, (B.L. Fisher et al.) (CASC); **MAYOTTE**: Dapani, –12.96279, 45.15037, 135 m, rainforest, (B.L. Fisher et al.) (CASC); Reserve forestière Sohoa, –12.81237, 45.10476, 10 m, coastal dry forest, (B.L. Fisher et al.) (CASC); Mont Combani, –12.80632, 45.15314, 370 m, rainforest, (B.L. Fisher et al.) (CASC); Mont Benara, –12.87585, 45.15672, 425 m, rainforest, (B.L. Fisher et al.) (CASC); Baie de Tsingoni, –12.7926, 45.10764, 5 m, mangrove, coastal scrub, (B.L. Fisher et al.) (CASC); Mont Chongui, –12.95776, 45.13403, 470 m, rainforest, (B.L. Fisher et al.) (CASC); Mont Chongui, –12.95903, 45.13411, 380 m, rainforest, (B.L. Fisher et al.) (CASC); Mont Chongui summit, –12.99567, 45.13428, 550 m, rainforest, (B.L. Fisher et al.) (CASC); Coconi, DAF Campus, –12.83333, 45.13333, (R. Jocqué) (CASC); Dziani Karihani, –12.78333, 45.11667, forest (R.Jocque & G.DeSmet) (CASC); Mont Combani, –12.80632, 45.15314, 370 m, rainforest, (B.L. Fisher et al.) (CASC).

##### Diagnosis.

In profile, anterior and posterior margins of petiolar node convex; in profile, propodeal dorsum and declivitous surface separated by blunt angle; in dorsal view, mesonotum less than twice as broad as long; mesopleuron with propodeal surface distinctly wider together than lateral portion of pronotum; in profile, propodeal dorsum roughly as long as declivitous margin; dorsum of head and mesosoma densely and finely reticulate-punctate; erect hairs lacking on dorsum of pronotum; distance between meso-metapleural suture and dorsolateral margin of propodeum largest near the junction of dorsolateral carina to declivitous surface; in dorsal view, lateral margins of mesonotum roughly straight and gradually converging posteriorly; width of propodeum at the metanotal groove less than half the maximum width of mesonotum; in full-face view, anteromedian margin of clypeus truncate.

##### Description.

**Minor worker** (Figs 14B, 15B, 16A, 23). Head elongate in full-face view (CWb/CL: 0.87–0.93), slightly diverging posteriorly; posterior margin convex medially and more or less straight near posterolateral corners; lateral margins slightly convex. Eyes larger relative to head size (EL/CS: 0.24–0.28), their anterior level located at about mid-length of head (PrOc/CL: 0.52–0.58). Anterior clypeal margin truncate; posterior margin weakly notched medially. Mandible triangular, apical margin armed with six sharp teeth which reduce in size towards basal angle of the mandible. Antennal scape short (SL/CS: 0.89–1.06), apical third of its length surpassing posterior cephalic margin. Pronotum dorsally flat, anterodorsal angle marginate. In dorsal view, mesonotum less than twice as broad as long, posterodorsal corner rounded. In lateral view, propodeum not strongly compressed anteroposteriorly, dorsum strongly inclined posteriorly and separated with declivitous surface by blunt angle; mesopleuron with propodeal surface together distinctly wider than lateral portion of pronotum; propodeal dorsum and lateral surface separated by blunt margination; propodeal spiracle on declivitous surface. Coxa of foreleg larger than width of meso-metapleuron. In profile, anterior margin of petiolar node convex, posterior margin inclined posteriorly and then approximately vertically straight to posteroventral angle. Constriction between abdominal segments III and IV lacking.

Dorsum of head and mesosoma finely and densely reticulate punctate; lateral surfaces of head finely and densely reticulate punctate with much smaller punctures. Imbricate sculpture on gastral tergites. Mandible smooth and shiny with sparse piligerous punctures. Whitish hairs lacking on pronotum; several pairs on head dorsum from clypeus, frontal lobe to posterior portion of head; one pair on mesonotum; few pairs arranged transversely at mid-height of posterior face of propodeum; hairs arranged near lateral and dorsal borders of posterior face of petiolar node; scattered and much shorter erect hairs arranged near anterior and posterior margins of each gastral tergite; pubescence not abundant. Color of body, femur, and tibia black; trochanter and tarsi brown to light brown; antenna brown basally and dark brown apically.

**Major worker.** With characteristics of minor worker, except: head in full-face view feebly longer than broad (CWb/CL: 0.94–1) and slightly decreasing in width towards the base of mandibles; posterior margin slightly convex; sides broadly convex. Eyes smaller relative to head size (EL/CS: 0.22–0.24), their posterior level located roughly at posterior fourth of head (PoOc/CL: 0.27–0.29). Anterior margin of clypeus truncate and slightly concave. Antennal scape slightly extending beyond posterior cephalic margin. In dorsal view, metanotum visible between metanotal groove and propodeum. In profile, petiolar node much more flattened anteroposteriorly. Head with scattered piligerous punctures laterally near base of mandibles. Dorsum of pronotum with few pairs of whitish erect hairs.

##### Distribution and biology.

*Camponotus edmondi* is known from Madagascar, Comore, and Mayotte Islands (Fig. 38). In Madagascar, it is generally distributed along the eastern littoral forests and in human-modified habitats. In neighboring islands, the species occurs also in coastal forests and disturbed forest habitats. Rarely is it found in rainforest between 130 m and 650 m of altitude. Foraging is done arboreally and nests sites are in dead twigs and branches above the ground.

##### Discussion.

*Camponotus edmondi* looks similar to *Camponotus orombe* and *Camponotus tafo*, but for *Camponotus orombe* there is no distinct angle separating the propodeal dorsum from the declivitous margin in profile, and the distance between the meso-metapleural suture and the dorsolateral margin of the propodeum remains the same along the dorsolateral carina of the propodeum. As in *Camponotus tafo*, the lateral margins of mesonotum are convex and converge strongly posteriorly while the width of the propodeum at the metanotal groove is more than half the maximum width of the mesonotum. *Camponotus mifaka* has numerous hairs on the dorsum of the mesosoma.

*Camponotus edmondi ernesti* was created by Forel (1891) because of its smaller body size, finer sculpture, and the shape of its propodeum. We were not able to examine the type specimen, but based on the observation of the specimens belonging to this subspecies, collected by Mocquerys in the Antongil Bay, and located at MSNG (Italy), there is no strong distinctive morphological traits between the members of the subspecies and those of *Camponotus edmondi*. Therefore, *Camponotus edmondi ernesti* is synonymized under *Camponotus edmondi*. As *Camponotus edmondi* is more or less widespread in the littoral forests of the Malagasy region, morphological variation within this species can be expected.

The identity of *Camponotus edmondi* based on the traditional qualitative taxonomy has been detected by the multivariate morphometrics. The grouping of the samples of *Camponotus edmondi* generated by NC-clustering method is corroborated by confirmatory LDA with a classification success of 100%.

#### Camponotus ethicus

Taxon classificationAnimaliaHymenopteraFormicidae

Forel

**Figure 24.**
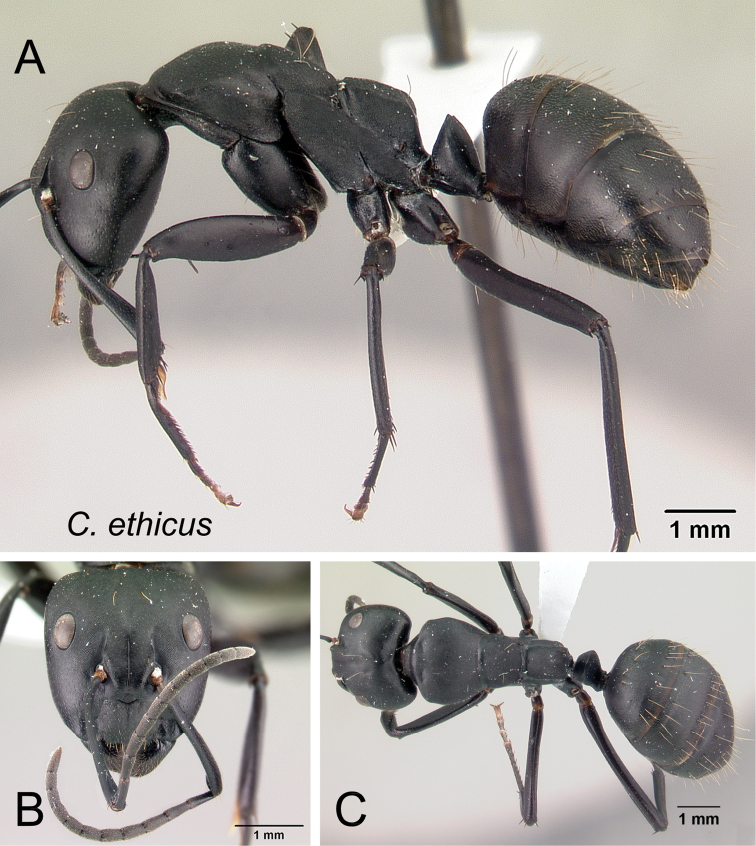
*Camponotus ethicus* minor worker CASENT0409948. **A** lateral view **B** head in full-face view **C** dorsal view.

Figures 6A , 7A , 24 , 39

Camponotus ethicus Forel, 1897: 200. Lectotype minor worker, **present designation**, Madagascar, Antsiranana Province, Sakatia bay, Nosy Be (Voeltzkow), AntWeb CASENT0101389 (MHNG) [examined]. Paralectotypes: 2 workers and 2 males, of same data as lectotype, but worker and male respectively specimen coded as: CASENT0101388 and CASENT0101387 (MHNG); CASENT0101176 and CASENT0101177 (NHMB) [examined]. [Combination in Camponotus (Myrmentoma): Forel 1912: 92; in Camponotus (Orthonotomyrmex): Forel 1914: 273; Emery 1920: 258; Wheeler 1922: 1049; in Camponotus (Myrmisolepis): Santschi 1921: 310; in Camponotus (Myrmepinotus): Emery 1925: 127].

##### Additional material examined.

**MADAGASCAR**: Province **Antsiranana**: Galoko chain, Mont Kalabenono, –13.64609, 48.67732, 937 m; –13.64179, 48.67282, 643 m; –13.63999, 48.67374, 498 m, rainforest, (B.L. Fisher et al.) (CASC); Réserve Spéciale d’Ambre, 3.5 km 235° SW Sakaramy, –12.46889, 49.24217, 325 m, tropical dry forest, (Fisher, Griswold et al.) (CASC); Province **Mahajanga**: Forêt d’Anabohazo, 21.6 km 247° WSW Maromandia, –14.30889, 47.91433, 120 m, tropical dry forest, (Fisher, Griswold et al.) (CASC).

##### Diagnosis.

Larger species (CS: 1.92–2.58; ML: 3.49–4.18) with uniformly black to dark brown body color; in profile anterior margin of petiolar node convex and posterior margin straight; level of the propodeal dorsum abruptly lower than level of the promesonotal dorsum; pronotal dorsum without numerous erect hairs; humeral angle extended anteriorly into a narrow ridge.

##### Description.

**Minor worker** (Figs 6A, 7A, 24). In full-face view head subquadrate (CWb/CL: 0.87–0.89), slightly diverging posteriorly; posterior margin more or less straight. Eyes not breaking lateral outline of head, their posterior level located at posterior fifth portion of head (PoOc/CL: 0.21–0.25). Anterior clypeal margin straight; posterior margin weakly notched medially. Mandible triangular, apical margin armed with six sharp teeth. Antennal scape long, apical half surpassing posterior cephalic margin. Pronotal dorsum flat, anterodorsal corner projecting anteriorly into narrow ridge; anterior margination present; pronotal dorsum and lateral portion anteriorly separated by sharp margination. In dorsal view, mesonotum as long as broad; in profile, mesonotal dorsum inclined posteriorly and propodeal dorsum nearly horizontal and distinctly situated at lower level than promesonotum; mesopleuron and propodeal surface together distinctly longer than lateral portion of pronotum; propodeal dorsum almost horizontal and declivitous surface nearly vertical; propodeal spiracle located anterior to posterolateral margin of propodeum. Width of procoxa larger than width of mesopleuron. In profile anterior margin of petiolar node convex and posterior margin more or less straight. Constriction between abdominal segments III and IV lacking.

Dorsum of head and mesosoma with fine and dense imbrication. Imbricate sculpture much finer and denser on gastral tergites. Mandible imbricate and superimposed with sparse large punctures. Erect hairs lacking on pronotum; one pair present on mesonotum, propodeum near junction of dorsum and declivity, and upper level of lateral margin of petiole. Few pairs of erect hairs on head dorsum from clypeus and edge of frontal lobe to posterior portion of head; several scattered pairs organized transversely on anterior and posterior portions of each gastral tergite; pubescence short and reduced in number. Body coloration black; appendages dark reddish black.

**Major worker.** With characteristics of minor worker, except: head much more square (CL/CWb: 1.008–1.08); lateral margins slightly convex. Eyes located more anteriorly, their posterior level on posterior fourth of head (PoOc/CL: 0.227–0.252). One third of apical portion of scape extending beyond posterior cephalic margin. Scattered piligerous punctures present laterally on head from the level of anterior margin of eyes to near base of mandible.

##### Distribution and biology.

Known from the dry forests of the Parc National Sahamalaza and the Reserve Spéciale Ambre, and the transitional rainforest of the Galoko Chain in the northwestern part of Madagascar (Fig. 39), *Camponotus ethicus* is arboreal, forages most often on lower vegetation, and nests in dead branches above the ground.

##### Discussion.

The lower level of the propodeal dorsum relative to the promesonotum and the larger body size combined with the dark color of the legs make *Camponotus ethicus* easy to separate from *Camponotus robustus* and the rest of the species in the *edmondi* group. The delimitation of *Camponotus ethicus* based on qualitative morphology-based taxonomy is congruent with the classification hypothesis provided by the NC-clustering algorithm, strengthening its status as a species.

#### Camponotus galoko

Taxon classificationAnimaliaHymenopteraFormicidae

Rakotonirina, Csősz & Fisher sp. n.

http://zoobank.org/0DE61664-AFA1-4B06-A0BF-542C74E29F20

**Figure 25.**
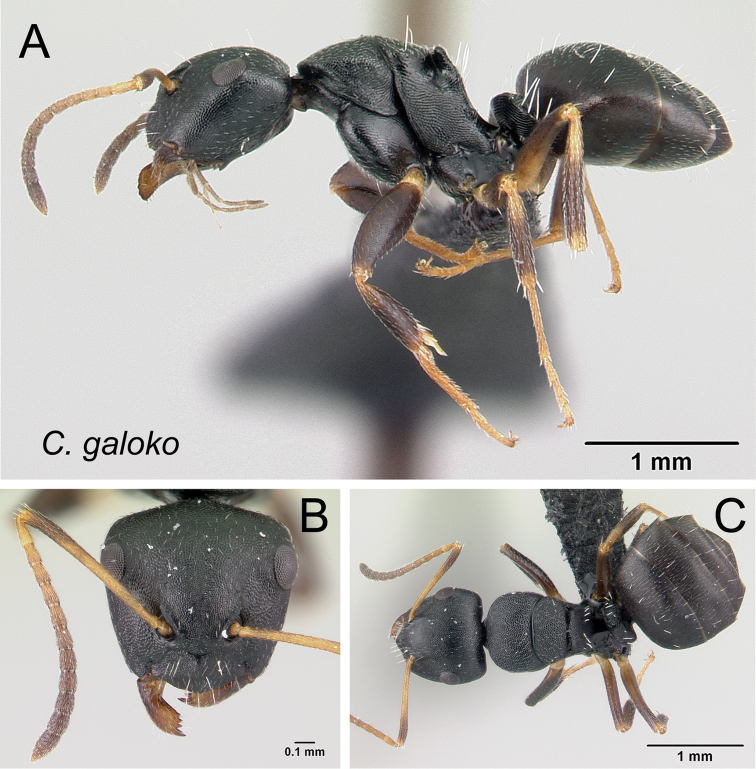
*Camponotus galoko* minor worker CASENT0178918. **A** lateral view **B** head in full-face view **C** dorsal view.

Figures 10B , 11B , 25 , 40

##### Holotype worker.

Madagascar, Province Antsiranana, Forêt de Binara, 9.1 km 233° SW Daraina, –13.26333, 49.60333, 650–800 m, rainforest, ex rotten log, 5 Dec 2003 (B.L. Fisher et al.) collection code BLF09814, specimen code CASENT0178918 (CASC).

##### Paratypes.

8 workers same data as holotype but with the following specimen codes: CASENT0076246, CASENT0076247, CASENT0076248, CASENT0746972, CASENT0746973, CASENT0746974, CASENT0746975, CASENT0746976 (BMNH, MHNG, MSNG, CASC).

##### Additional material examined.

**MADAGASCAR**: Province **Antsiranana**: Forêt de Binara, 9.1 km 233° SW Daraina, –13.26333, 49.60333, 650–800 m, rainforest, (B.L. Fisher) (CASC), Galoko chain, Mont Galoko, –13.5888, 48.72864, 980 m, montane forest, (B.L. Fisher et al.) (CASC); Galoko chain, Mont Galoko, –13.59358, 48.73157, 1100 m, montane forest, (B.L. Fisher et al.) (CASC); Galoko chain, Mont Kalabenono, –13.64609, 48.67732, 937 m, rainforest, (B.L. Fisher et al.) (CASC).

##### Diagnosis.

In profile, anterior and posterior margins of petiolar node convex; in profile, propodeum strongly compressed anteroposteriorly, without clear distinction between dorsal margin and declivity; in dorsal view, mesonotum twice as broad as long; posterodorsal corner of mesonotum without extended shield.

##### Description.

**Minor worker** (Figs 10B, 11B, 25). In full-face view head slightly longer than broad (CWb/CL: 0.94–0.99), slightly diverging posteriorly; posterior margin broadly convex, lateral margins roughly straight. Eyes larger relative to size of head (EL/CS: 0.23–0.25), their posterior level located at about posterior fourth of head (PoOc/CL: 0.2–0.25). Anterior clypeal margin transverse; posterior margin medially notched. Mandible triangular, apical margin armed with six sharp teeth, which reduce in size towards basal angle of the mandible. Antennal scape short (SL/CS: 0.81–0.98), one fourth of the length surpassing posterior cephalic margin. Pronotum flat dorsally, anteriorly projecting into narrow ridge; dorsolateral portion longitudinally marginate. In dorsal view, mesonotum twice as broad as long, posterodorsal corner rounded, without extended lobe; lateral margin convex and strongly convergent posteriorly. Propodeum strongly compressed anteroposterioly, dorsal margin and declivity not distinctly separated; posterolateral portion extending laterally into sharp ridge. Propodeal spiracle on lower third of posterior face of propodeum. Maximum width of procoxa as large as the width of meso-metapleuron and propodeal surface together; femur of foreleg enlarged, twice as large as those of mid-leg and hind leg. Anterior and posterior margins of petiolar node convex. No constriction between abdominal segments III and IV.

Dorsum of head and mesosoma finely and densely reticulate punctate. Mandible finely and densely reticulate superimposed with scattered large punctures. Finer and denser reticulate punctures present on gastral tergites. Pronotum with a few pairs and mesonotum with one pair of whitish erect hairs; whitish hairs gathered at mid-height of posterior face of propodeum; whitish erect hairs present at mid-height of near lateral and on dorsal margins of posterior face of petiolar node; gastral segments with scattered and much shorter erect hairs; pubescence more abundant on gastral tergite than mesosomal dorsum. Integument shining black, antenna brown basally and darker apically; basal portion of mandible and leg dark brown, apical portion and trochanter light brown to yellowish-orange.

**Major worker.** Characteristics of minor worker, except: head in full-face view roughly as long as broad (CWb/CL: 0.96–1.03), lateral margins slightly convex and slightly converging near base of mandibles. Eyes smaller relative to head size (EL/CS: 0.19–0.22), their posterior level located roughly at posterior fourth of head (PoOc/CL: 0.27–0.31). Anterior margin of clypeus truncate and posterior. Antennal scape not extending beyond posterior cephalic margin. In dorsal view, metanotum visible between metanotal groove and propodeum. In lateral view, petiolar node more compressed anteroposteriorly. Lateral portion of head near base of mandible with sparse, large, piligerous punctures.

##### Distribution and biology.

This species is known only from the transitional humid forests of the Daraina and Galoko chain in the north of Madagascar (Fig. 40). The data indicate that individual workers forage on lower vegetation, while nests are mostly found in dead twigs above the ground and rarely in rotten logs.

##### Discussion.

*Camponotus galoko* is mostly similar to *Camponotus echinoploides*, but the posterodorsal corner of the mesonotum in the latter raises into a bluntly rounded shield. *Camponotus galoko* has a strongly anteroposteriorly flattened propodeum without a clear distinction between the propodeal dorsum and the declivity while the other species in the *edmondi* group have a propodeal dorsum and a declivitous surface separated by a blunt angle.

The taxonomic argument for *Camponotus galoko* is strengthened by the congruence between the results of traditional qualitative morphology and the NC-clustering technique. However, the classification success is only 90.91%, because its one minor worker is misclassified as *Camponotus varatra* by the confirmatory LDA with a low posterior probability of 0.76. This suggests that the entire range of minor worker forms of these species might not have been measured, and both species are closely related and have similar quantitative and qualitative morphology. Yet the two are distinguished by a morphological trait not easily incorporated into the morphometric approach. The dorsum of the head and mesosoma of *Camponotus galoko* are densely and finely reticulate whereas those of *Camponotus varatra* and *Camponotus zavo* are smooth, shining, and superimposed by imbrication.

#### Camponotus matsilo

Taxon classificationAnimaliaHymenopteraFormicidae

Rakotonirina, Csősz & Fisher sp. n.

http://zoobank.org/0364FA72-21E3-4C38-9944-AAA36894D9AA

**Figure 26.**
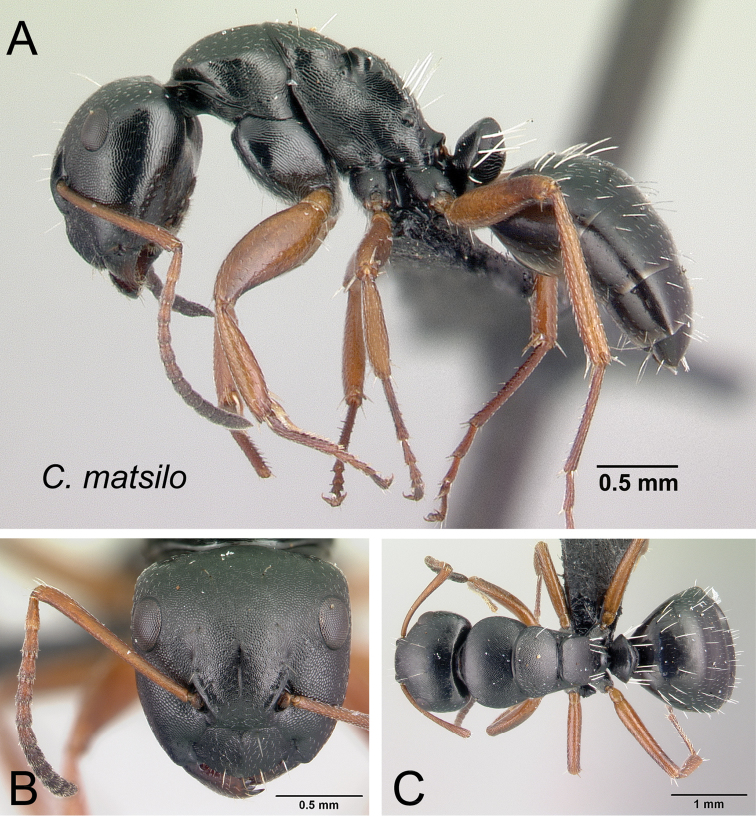
*Camponotus matsilo* minor worker CASENT0121843. **A** lateral view **B** head in full-face view **C** dorsal view.

Figures 12A , 26 , 41

##### Holotype worker.

Madagascar, Province Toliara, Forêt Vohidava 88.9 km N Amboasary, –24.24067, 46.28783, 500 m, spiny forest/dry forest transition, ex dead twig above ground, 7 Dec 2006 (B.L. Fisher et al.) collection code BLF15725, specimen code CASENT0121843 (CASC).

##### Paratypes.

1 worker with same data as holotype but specimen coded as CASENT0178919 (CASC).

##### Additional material examined.

**MADAGASCAR**: Province **Toliara**: Forêt Vohidava 88.9 km N Amboasary, –24.24067, 46.28783, 500 m, spiny forest/dry forest transition, (B.L. Fisher et al.) (CASC); Parc National d’Andohahela, Forêt d’Ambohibory, 1.7 km 61° ENE Tsimelahy, 36.1 km 308° NW Tolagnaro, –24.93 46.6455, 300 m, tropical dry forest, (Fisher-Griswold Arthropod Team) (CASC); Parc National d’Andohahela, Forêt de Manatalinjo, 33.6 km 63° ENE Amboasary, 7.6 km 99° E Hazofotsy, –24.81694, 46.61, 150 m, spiny forest/thicket, (Fisher-Griswold Arthropod Team) (CASC); Parc National de Zombitse, 19.8 km 84° E Sakaraha, –22.84333, 44.71, 770 m, tropical dry forest, (Fisher, Griswold et al.) (CASC); Réserve Spéciale de Cap Sainte Marie, 14.9 km 261° W Marovato, –25.59444, 45.14683, 160 m, spiny forest/thicket, (Fisher-Griswold Arthropod Team) (CASC).

##### Diagnosis.

In profile, anterior and posterior margins of petiolar node convex; in profile, propodeal dorsum and declivitous surface separated by blunt angle; in dorsal view, mesonotum less than twice as broad as long; mesopleuron with propodeal surface together distinctly wider than lateral portion of pronotum; in profile, dorsolateral carina of propodeum much longer than its posterolateral margin.

##### Description.

**Minor worker** (Figs 12A, 26). In full-face view head slightly longer than broad (CWb/CL: 0.88–0.94), lateral margins weakly convex and converging anteriorly; posterior margin feebly convex. Eyes located more on posterior portion of head (PoOc/CL: 0.18–0.23), posterior level of eyes at posterior fifth of head. Anteromedian margin of clypeus triangular; posterior margin weakly notched medially. Mandible triangular, masticatory margin armed with six teeth. Antennal scape short (SL/CS: 0.87–0.94), apical third portion roughly surpassing posterior margin of head. Pronotum flat dorsally, anterodorsal margin projecting anteriorly into narrow ridge; dorsum and sides of promesonotum separated by margination. In dorsal view, mesonotum narrow, less than twice as broad as long. In profile, mesopleuron and lateral propodeal face together distinctly longer than lateral portion of pronotum; propodeal dorsum and declivitous surface separated by blunt angle. In profile, dorsolateral carina of propodeum much longer than declivity. Maximum width of procoxa larger than width of meso-metapleuron. In profile, anterior margin of petiolar node convex and posterior margin either convex or roughly triangular; propodeal spiracle located on declivitous surface or at posterolateral margin of the propodeum.

Dorsum of head and mesosoma finely and densely reticulate punctate. Gastral segments covered with fine and dense reticulation. Mandible finely and densely reticulate superimposed with scattered large punctures. Whitish erect hairs: several pairs of on dorsum of head; absent on pronotum, one pair on mesonotum, few pairs near dorsolateral margin of propodeum and at junction of dorsum and declivity, arranged on posterior face of petiolar node near lateral margins; organized transversely on anterior and posterior portions of each gastral tergite. Pubescence not abundant. Head, mesosoma, and petiole black; gaster dark brown; basal portion of antenna light brown to yellow and apical portion dark brown; trochanter to tip of tarsi light brown to yellow.

**Major worker.** With characteristics of minor worker, except: head much more subquadrate (CWb/CL: 0.96); eyes located roughly on posterior third of head capsule (PoOc/CL: 0.28–0.29); antennal scape not extending beyond posterior cephalic margin; inclination of propodeal dorsum much more vertical in profile. Head capsule microreticulate superimposed with scattered punctures in the anterior portion from the anterior level of eyes to base of mandibles. Mandibles smooth and shiny between sparse punctures. Two pairs of whitish erect hairs aligned at about the same level on posterior portion of head behind posterior level of eyes; three to four pairs on pronotum and five pairs on mesonotum. Mandible color much darker than other appendages.

##### Distribution and biology.

Occurring in the south of Madagascar, the distribution of *Camponotus matsilo* is ranging from the dry forest habitats of the PN Zombitse and the PN Andohahela through the transitional spiny forest of Vohidava to the spiny bush and thicket of Cap Sainte Marie in the extreme south (Fig. 41). In these habitats, the species mostly forages on low vegetation and its colonies are found frequently in dead twigs above ground.

##### Discussion.

*Camponotus matsilo* can be easily separated from other species by the fact that its propodeal dorsum is distinctly longer than its propodeal declivity in lateral view.

The qualitative morphology-based study of this species agrees with the multivariate morphometric analysis to support the taxonomic determination for *Camponotus matsilo*.

#### Camponotus mifaka

Taxon classificationAnimaliaHymenopteraFormicidae

Rakotonirina, Csősz & Fisher sp. n.

http://zoobank.org/2A5DC5AD-5C2A-4BBE-B427-5ABD020752C7

**Figure 27.**
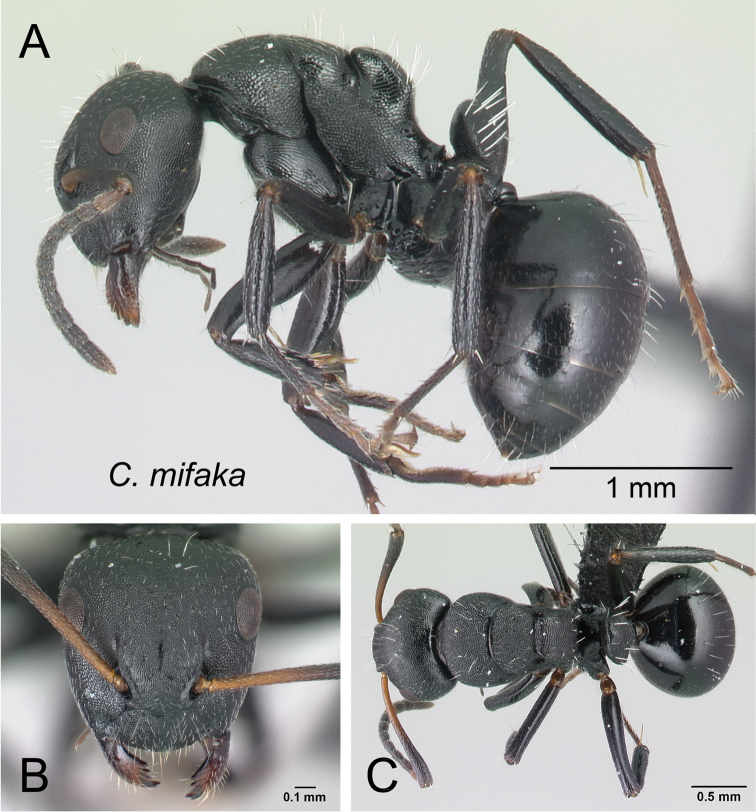
*Camponotus mifaka* minor worker CASENT0217301. **A** lateral view **B** head in full-face view **C** dorsal view.

Figures 14A , 27 , 42

##### Holotype worker.

Madagascar, Antsiranana, Parc National de Marojejy, 25.4 km 30° NNE Andapa, 10.9 km 311° NW Manantenina, –14.445, 49.735, 2000 m, montane shrubland, ex root mat, ground layer, 24 Nov 2003 (B.L. Fisher et al.) collection code BLF09351, specimen code CASENT0217301 (CASC).

##### Paratypes.

8 workers same data as holotype but with the following specimen codes: CASENT0486999, CASENT0487000, CASENT0487001, CASENT0746965, CASENT0746966, CASENT0746967, CASENT0746970, CASENT0746971 (BMNH, MHNG, MSNG, CASC).

##### Additional material examined.

**MADAGASCAR:** Province **Antsiranana**: Parc National Marojejy, 25.4 km 30° NNE Andapa, 10.9 km 311° NW Manantenina, –14.445, 49.735, 2000 m, montane shrubland, (B.L. Fisher) (CASC).

##### Diagnosis.

In profile, anterior and posterior margins of petiolar node convex; in profile, propodeal dorsum and declivitous surface separated by blunt angle; in dorsal view, mesonotum less than twice as broad as long; mesopleuron with propodeal surface together distinctly wider than lateral portion of pronotum; in profile, propodeal dorsum roughly as long as declivitous margin; dorsum of head and mesosoma densely and finely reticulate punctate; dorsum of mesosoma covered with numerous erect hairs and pubescence.

##### Description.

**Minor worker** (Figs 14A, 27). In full-face view, head elongate (CWb/CL: 0.9–0.95), diverging posteriorly; posterior margin slightly convex. Level of posterior ocular margins located generally on posterior fifth portion of head (PoOc/CL: 0.21–0.25). Anterior margin of clypeus straight; posterior margin medially notched. Mandible triangular, apical margin armed with six sharp teeth. Antennal scape relatively long (SL/CS: 0.92–1.02), more than one third of apical portion of antennal scape extending beyond posterior cephalic margin. Promesonotum slightly, broadly convex, dorsum and sides separated by margination; anterodorsal angle of pronotum projecting anteriorly into a ridge. In dorsal view, mesonotum less than twice as broad as long; posterodorsal angle without extended lobe. In lateral view, propodeum not strongly compressed anteroposteriorly; dorsal portion of propodeum raised and abruptly strongly sloping posteriorly; junction to declivity marked by blunt angle; dorsolateral portion of propodeum marginate, distance between meso-metapleural suture and dorsolateral margin of propodeum remaining the same along dorsolateral margin of propodeum; propodeal spiracle located on declivitous surface. Width of meso-metapleuron and propodeal lateral portion together noticeably greater than width of lateral portion of pronotum. In side view, maximum width of procoxa larger than width of meso-metapleuron. In profile, anterior and posterior margins of petiolar node convex. Junction of abdominal segments III and IV without visible constriction.

Dorsum of head anteriorly finely and densely reticulate punctate, the punctures deepening posteriorly. Mandible smooth and shining between scattered punctures. Mesosoma dorsum finely and densely reticulate punctate. Gastral tergites smooth and shining apart from shallow punctures from which erect hairs or pubescence arise. Pairs of whitish erect hairs numerous on dorsum of head and mesosoma; hairs randomly scattered on gastral tergites. Erect hair present on declivitous surface above propodeal spiracle. Near posterolateral margins to posterodorsal corner of petiolar node with a row of whitish erect hairs. Much shorter and sparse erect hairs organized transversely on anterior and posterior portions of each gastral tergite; pubescence reduced. Integument generally black; basal portion of legs dark brown and becoming lighter towards metatarsi; antennal scape basally brown and apically black to dark brown.

**Major worker.** With characteristics of minor worker, except: head larger relative to whole body size (ML/CS: 1.29–1.35); shape relatively subquadrate (CWb/CL: 0.93–0.95) with lateral margins slightly converging to base of mandibles; posterior margin more or less straight. Eyes positioned more on frontal portion of head, level of posterior ocular margins located approximately on posterior fourth portion of head (PoOc/CL: 0.23–0.25); mandible strong; one sixth of apical portion of antennal scape surpassing posterior cephalic margin (SL/CS: 0.92–0.96). Scattered punctures on dorsolateral portion of head near base of mandible.

##### Distribution and biology.

Known only from the montane shrubland of the Parc National Marojejy (Fig. 42), *Camponotus mifaka* forages in leaf mold and rotten wood and nests under root mats in the ground.

##### Discussion.

*Camponotus mifaka* might be confused with *Camponotus edmondi*, *Camponotus orombe*, and *Camponotus tafo* because of the dense and fine reticulate-punctate sculpture on the dorsum of the head and mesosoma; however, the latter three species have a reduced number of erect hairs on the dorsum of the mesosoma, particularly on the promesonotal dorsum.

Based on the information provided by the NC-clustering method, the cluster of *Camponotus mifaka* contains one sample of *Camponotus varatra*, but is classified successfully at 100% by LDA. The integration of this successful classification with the results from qualitative morphological study and biological evidence underscores the robustness of the taxonomic determination for this species. *Camponotus varatra* differs morphologically from *Camponotus mifaka* by its imbricate sculpture and biologically by its nesting sites in dead twigs or branches above the ground.

#### Camponotus orombe

Taxon classificationAnimaliaHymenopteraFormicidae

Rakotonirina, Csősz & Fisher sp. n.

http://zoobank.org/C02F05C1-3464-402D-BCA9-914AA06EADBD

**Figure 28.**
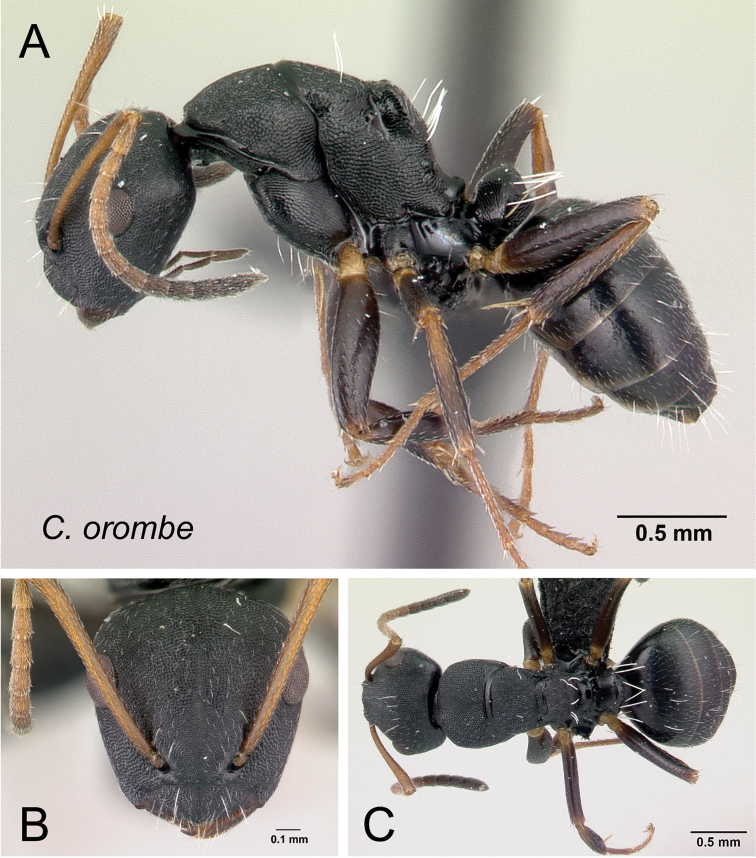
*Camponotus orombe* minor worker CASENT0178923. **A** lateral view **B** head in full-face view **C** dorsal view.

Figures 15A , 28 , 43

##### Holotype worker.

Madagascar, Province Toliara, Forêt Ivohibe 55.0 km N Tolagnaro, –24.569, 47.204, 200 m, rainforest, ex dead twig above ground, 12 Mar 2006 (B.L. Fisher et al.) collection code: BLF15534, specimen code: CASENT0178923 (CASC).

##### Paratypes.

3 workers, 1 worker same data as holotype but with specimen code CASENT0122867; 2 workers with the following data: Forêt Ivohibe 55.6 km N Tolagnaro, –24.56167, 47.20017, 650 m, rainforest, beating low vegetation, 12 Apr 2006, BLF15587 and CASENT0122787, BLF15628 and CASENT0121500 (BMNH, CASC).

##### Additional material examined.

**MADAGASCAR**: Province **Toliara**: Forêt Ivohibe 55.0 km N Tolagnaro, –24.569, 47.204, 200 m, rainforest, (B.L. Fisher et al.) (CASC); Forêt Ivohibe 55.6 km N Tolagnaro, –24.56167, 47.20017, 650 m, rainforest, (B.L. Fisher et al.) (CASC).

##### Diagnosis.

In profile, anterior and posterior margins of petiolar node convex; in profile, propodeal dorsum and declivitous surface separated by blunt angle; in dorsal view, mesonotum less than twice as broad as long; mesopleuron with propodeal surface together distinctly wider than lateral portion of pronotum; in profile, propodeal dorsum roughly as long as declivitous margin; dorsum of head and mesosoma densely and finely reticulate punctate; erect hairs lacking on dorsum of pronotum; distance between meso-metapleural suture and dorsolateral margin of propodeum not changing along the dorsolateral carina of propodeum.

##### Description.

**Minor worker** (Figs 15A, 28). In full-face view head slightly longer than broad (CWb/CL: 0.88); lateral margins weakly convex and converging slightly towards base of mandibles; posterior border broadly convex. Level of posterior ocular margins at about posterior fourth portion of head (PoOc/CL: 0.23–0.24). Anteromedian margin of clypeus with slightly blunt angle; posterior margin weakly notched. Mandible triangular, masticatory margin with six teeth. Antennal scape relatively long, distal portion almost extending beyond posterior border of head. In lateral view, pronotum dorsally flat, anterior margin projecting into narrow ridge; dorsolateral portion of promesonotum longitudinally marginate. In dorsal view, mesonotum less than twice as broad as long, posterodorsal corner without visible posterior lobe; lateral margin convex and gradually converging to metanotal groove. In lateral view, propodeum strongly compressed anteroposteriorly, junction between dorsal margin and declivity not distinctly visible; ridge on posterolateral portion distinct; distance between meso-metapleural suture and posterolateral ridge of propodeum remaining the same along dorsolateral carina of propodeum. Propodeal spiracle on lower third of posterior face of propodeum. Maximum width of procoxa as large as width of meso-metapleuron and propodeal surface together. In lateral view, anterior margin of petiolar node convex; posterior margin sloping posteriorly to about mid-height and descending almost vertically to posteroventral angle. No constriction between abdominal segments III and IV.

Dorsum of head, mesosoma, and petiolar node with fine and dense reticulate punctures. Mandible with sparse piligerous punctures between smooth surfaces. Numerous pairs of whitish erect hairs on dorsum of head; one pair on mesonotum; numerous erect hairs arranged along junction of propodeal dorsum and declivity. No erect hair on declivitous surface above propodeal spiracle level. Posterior face of petiole with a row of four erect hairs near lateral margin and posterodorsal angle. Shorter and slender erect hairs organized transversely on anterior and posterior portions of each gastral tergite. Pubescence present on dorsum of head, mesosoma, petiole and gaster. Head, mesosoma, and petiole black in color; antennal scape and first five flagellar segments, mandible, trochanter, and tarsus yellow to light brown; gaster, apical portion of antennal segments, femur, and tibia dark brown.

**Major worker.** Similar to minor worker, but with the following divergent characters: in full-face view, head subquadrate (CWb/CL: 0.98), lateral borders almost parallel and converging strongly near base of mandibles; level of posterior margins of eyes located at about posterior third portion of head (PoOc/CL: 0.3). Anterior margin of clypeus medially excised; mandible robustly built; antennal scape barely surpassing posterior cephalic border. Anterior portion of pronotum not strongly marginate; in dorsal view, metanotum visible between metanotal groove and propodeum. Junction of dorsum and sides of propodeum more or less rounded; petiolar node more flattened anteroposteriorly.

##### Distribution and biology.

*Camponotus orombe* is known only from a few individual workers collected from Ivohibe Forest between 200 m and 650 m of altitude in the southeast of Madagascar (Fig. 43). They were found foraging on low vegetation and nesting in dead twigs above the ground.

##### Discussion.

*Camponotus orombe* can be confused to *Camponotus mifaka*, but the latter has numerous erect hairs on the dorsum of its mesosoma. *Camponotus orombe* can be differentiated from *Camponotus tafo* and *Camponotus edmondi* by the fact that the propodeum of these two latter species is not strongly compressed anteroposteriorly and the distance between the meso-metapleural suture and the dorsolateral margin of the propodeum is largest near the junction of the dorsolateral carina and the declivitous surface.

In the morphometric dendrogram, *Camponotus orombe* is represented by a successfully classified small cluster of three individual specimens that falls close to the cluster of *Camponotus varatra*, *Camponotus tafo*, *Camponotus mifaka*, *Camponotus zavo*, and *Camponotus tratra*. Based on qualitative morphology, *Camponotus varatra*, *Camponotus zavo*, and *Camponotus tratra* differ from *Camponotus orombe* by the sculpture on the dorsum of their head and mesosoma, which is imbricate or smooth and shiny with sparse piligerous punctures. This information supports the separation of *Camponotus orombe* from the other three species.

#### Camponotus robustus

Taxon classificationAnimaliaHymenopteraFormicidae

Roger

**Figure 29.**
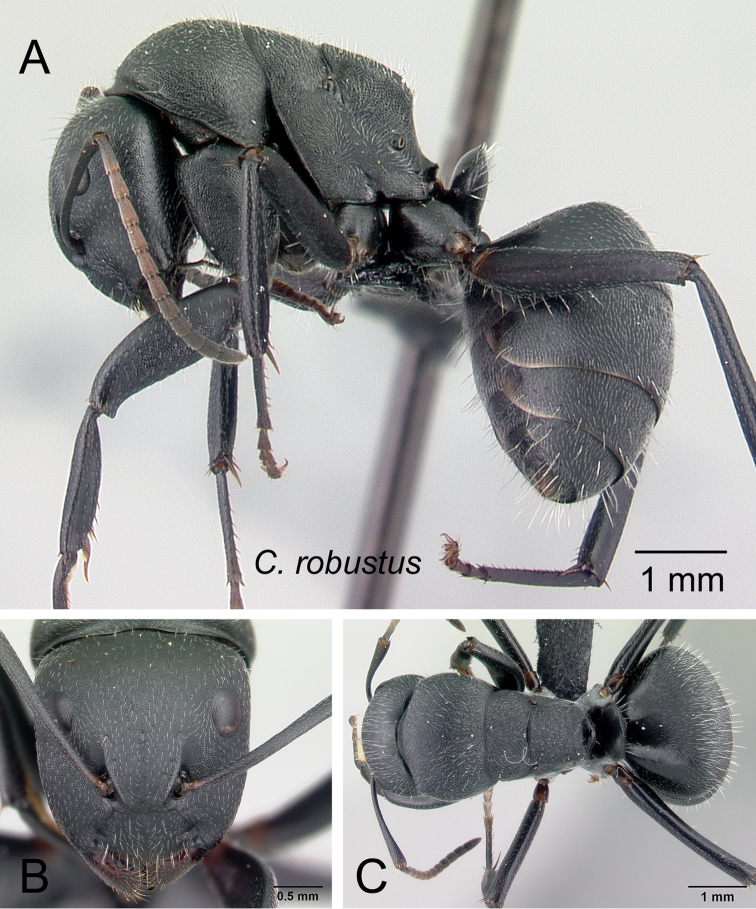
*Camponotus robustus* minor worker CASENT0066723. **A** lateral view **B** head in full-face view **C** dorsal view.

Figures 7B , 29 , 44

Camponotus robustus Roger, 1863: 135. Lectotype minor worker, **present designation**, Madagascar (Humblot), AntWeb CASENT0101390 (MHNG) [examined]. Paralectotypes of 5 workers: 2 in the same pin as lectotype; 2 workers with the same data but specimen coded as CASENT0104621 and CASENT0104622 (ZMHB) [examined]. Combination in Camponotus (Myrmentoma): Forel 1912: 92; in Camponotus (Orthonotomyrmex): Forel 1914: 273; Emery 1920: 258; Wheeler 1922: 1049; in Camponotus (Myrmisolepis): Santschi 1921: 310; in Camponotus (Myrmepinotus): Emery 1925: 127; Bolton 1995: 120, 131].

##### Additional material examined.

**MADAGASCAR**: Province **Antsiranana**: Forêt Ambanitaza, 26.1 km 347° Antalaha, –14.67933, 50.18367, 240 m, rainforest, (B.L. Fisher) (CASC); Forêt de Binara, 9.1 km 233° SW Daraina,–13.26333, 49.60333, 800 m, rainforest (B.L. Fisher et al.) (CASC); Makirovana forest, –14.10295, 50.01984, 90 m, rainforest (B.L. Fisher et al.) (CASC); Province **Fianarantsoa**: Réserve Forestière d’Agnalazaha, Mahabo, 42.9 km 215° Farafangana, –23.19383, 47.723, 20 m, littoral rainforest, (B.L. Fisher et al.) (CASC); Réserve Spéciale Manombo 24.5 km 228° Farafangana, –23.01583, 47.719, 30 m, rainforest, (B.L. Fisher et al.) (CASC); Province **Toamasina**: Ile Sainte Marie, Forêt Ambohidena, 22.8 km 44° Ambodifotatra, –16.82433, 49.96417, 20 m, littoral rainforest, (B.L. Fisher et al.) (CASC); Parc National Mananara-Nord, 7.1 km 261° Antanambe, –16.455, 49.7875, 225 m, rainforest, (B.L. Fisher et al.) (CASC); Station Forestière Tampolo, 10 km NNE Fenoarivo Atsinanana, –17.2825, 49.43, 10 m, littoral rainforest, (B.L. Fisher) (CASC); Sahafina Forest 11.4 km W Brickaville, –18.81445, 48.96205, 140 m, rainforest, (B.L. Fisher et al.) (CASC); Mahavelona (Foulpointe), –17.66667, 49.5, in sandy forest (A. Pauly) (CASC); Forêt d’Analava Mandrisy, 5.9 km 195º Antanambe, –16.48567, 49.847, 10 m, littoral rainforest, (B.L. Fisher et al.) (CASC); Réserve Ambodiriana, 4.8 km 306° Manompana, along Manompana River, –16.67233, 49.70117, 125 m, rainforest, (B.L. Fisher et al.) (CASC); Parc National Masoala, 39.7 km 151° SSE Maroantsetra, –15.71333, 49.97167, 150 m, rainforest, (B.L. Fisher, H.J. Ratsirarson) (CASC); 11km SE Ampasimanolotra (= Brickaville), –18.9, 49.13333, 5 m, littoral rainforest (P.S. Ward) (PSWC).

##### Diagnosis.

Larger species (CS: 1.882–3.725; ML: 3.098–4.666) with uniformly black to dark brown body color; in profile anterior margin of petiolar node convex and posterior margin straight; level of propodeal dorsum not abruptly lower than level of promesonotal dorsum; pronotum covered with numerous erect hairs and pubescence.

##### Description.

**Minor worker** (Figs 7B, 29). In full-face view head rectangular and longer than broad (CWb/CL: 0.91–0.98); lateral margins nearly straight and slightly diverging posteriorly; posterior margin broadly convex. Eyes not breaking lateral outline of head, posterior level located at posterior fourth portion of head (PoOc/CL: 0.21–0.27). Anterior clypeal margin broadly triangular. Mandible triangular, apical margin armed with six teeth. More than apical third portion of antennal scape surpassing posterior cephalic margin. Anterodorsal corner of pronotum projecting anteriorly into narrow ridge; anterior margination strong near corner and weak towards the center; pronotal dorsum rounding to lateral portion. In dorsal view, mesonotum broader than long. In lateral view, mesonotal dorsum slightly inclined posteriorly as is the propodeal dorsum, which joins the declivity at a blunt angle; mesopleuron and propodeal surface together distinctly longer than lateral portion of pronotum; propodeal spiracle located on lateral portion of propodeum anterior to posterolateral margin. Maximum width of procoxa larger than width of meso-metapleuron. In profile anterior margin of petiolar node convex and posterior margin more or less straight. Constriction between abdominal segments III and IV absent.

Dorsum of head, mesosoma, and gaster finely and densely reticulate rugose. Lateral portion of head finely and densely reticulate punctate. Mandible imbricate superimposed with punctures. Whitish yellow erect hairs numerous on head, mesosoma, and gaster. Petiole with erect hairs from lateral margins to posterodorsal angle. Pubescence abundant. Head, mesosoma, antennal scape, and distal portion of flagellum, femur, tibia, and basitarsus black; trochanter and metatarsi as well as basal portion of flagellum brown.

**Major worker.** With characteristics of minor worker, except: head broader than long (CWb/CL: 0.98–1.02); sides slightly convex and strongly converging towards base of mandibles. Eyes located more anteriorly, their posterior level on posterior third of head (PoOc/CL: 0.26–0.3). Antennal scape barely extending beyond posterior cephalic margin. Anteromedian portion of pronotum without margination. Lateral portion of head smooth and shining with scattered small punctures from which short hairs arise. Mandible with longitudinal striation near apical margin apart from fine imbrication and piligerous punctures.

##### Distribution and biology.

*Camponotus robustus* occurs in the rainforests of eastern Madagascar, from Binara in the north to Ivohibe Forest in the south (Fig. 44). The species is both terrestrial and arboreal. Its workers forage individually on the forest floor, on lower vegetation, and in the canopy and nest in rotten logs, rotting tree stumps, or in dead branches above the ground.

##### Discussion.

*Camponotus robustus* is similar to *Camponotus ethicus*, but the latter has no erect hairs on the dorsum of the mesosoma and has a propodeal dorsum lower than the promesonotum. The remainder of the *edmondi* group can be distinguished from this species by their smaller size and yellow to brown legs.

Species delimitation of *Camponotus robustus* based on traditional qualitative taxonomy is congruent with the grouping generated by the morphometric dendrogram and the species was classified correctly at 100% by the confirmatory LDA.

#### Camponotus tafo

Taxon classificationAnimaliaHymenopteraFormicidae

Rakotonirina, Csősz & Fisher sp. n.

http://zoobank.org/9236C3BC-645D-44C1-BD20-5A416996BD84

**Figure 30.**
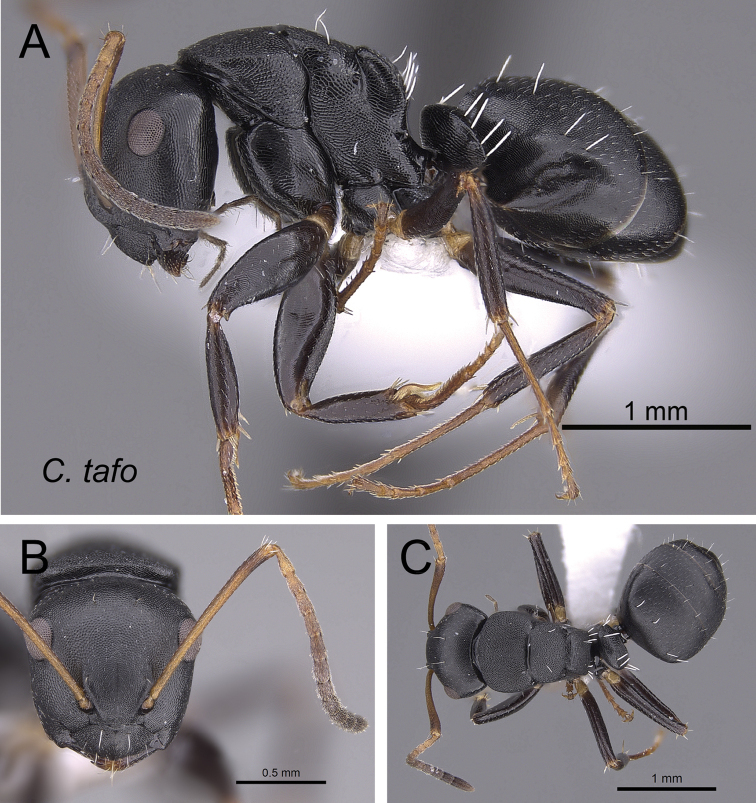
*Camponotus tafo* minor worker CASENT0763608. **A** lateral view **B** head in full-face view **C** dorsal view.

Figures 10D , 13A , 16B , 30 , 45

##### Holotype worker.

Madagascar, Toamasina Parc National de Masoala, 39.4 km 150° SSE Maroantsetra, –15.71, 49.97, 200 m, rainforest, canopy moss and leaf litter, 28 Nov-3 Dec 2001 (B.L. Fisher, H.J. Ratsirarson) collection code BLF04700 specimen code CASENT0763608 (CASC).

##### Paratypes.

4 workers same data as holotype but with specimen codes: CASENT0418183, CASENT0418184, CASENT0746968, CASENT0746969 (BMNH, MHNG, CASC).

##### Additional material examined.

**MADAGASCAR**: Province **Toamasina**: Ankerana Forest, –18.40829, 48.82107, 750 m, rainforest, (B.L. Fisher et al.) (CASC); Parc National Masoala, 39.4 km 150° SSE Maroantsetra, –15.71, 49.97, 200 m, rainforest, (B.L. Fisher, H.J. Ratsirarson) (CASC).

##### Diagnosis.

In profile, anterior and posterior margins of petiolar node convex; in profile, propodeal dorsum and declivitous surface separated by blunt angle; in dorsal view, mesonotum less than twice as broad as long; mesopleuron with propodeal surface together distinctly wider than lateral portion of pronotum; in profile, propodeal dorsum roughly as long as declivitous margin; dorsum of head and mesosoma densely and finely reticulate punctate; erect hairs lacking on dorsum of pronotum; distance between meso-metapleural suture and dorsolateral margin of propodeum largest near the junction of dorsolateral carina to declivitous surface; in dorsal view, lateral margins of mesonotum convex and strongly converging posteriorly; width of propodeum at metanotal groove greater than half the maximum width of mesonotum; in full-face view, anteromedian margin of clypeus triangular.

##### Description.

**Minor worker** (Figs 10D, 13A, 16B, 30). In full-face view head about as long as wide (CWb/CL: 0.91–0.97), lateral margins roughly straight and slightly converging anteriorly; posterior margin broadly convex. Eyes located on posterior fifth portion of head (PoOc/CL: 0.19–0.22). Anteromedian margin of clypeus triangular; posterior margin weakly notched medially. Mandible triangular, apical margin armed with six teeth reducing in size towards basal angle of mandible. Antennal scape long, roughly the apical half of its length surpassing posterior cephalic margin. Pronotum flat dorsally, anterodorsal margin projecting anteriorly into narrow ridge; dorsum and sides of promesonotum separated by margination. In dorsal view, mesonotum less than twice as broad as long, posterodorsal corner rounded. In lateral view, propodeum not strongly compressed anteroposteriorly; propodeal dorsum strongly sloping posteriorly; junction to declivity marked by blunt angle; in dorsal view, mesonotum longitudinally narrow, less than twice as broad as long; width of meso-metapleuron and side of propodeum together distinctly much greater than width of side of pronotum; dorsolateral portion of propodeum bluntly marginate; propodeal spiracle located on declivitous surface. Maximum width of coxa of foreleg larger than width of meso-metapleuron. In profile, anterior face of petiolar node convex, posterior face sloping posteriorly and then descending vertically to posteroventral angle. Constriction between abdominal segments III and IV absent.

Dorsum of head and mesosoma finely and densely reticulate punctate. Imbricate sculpture on gastral tergites. Mandible finely and densely reticulate superimposed with scattered large punctures. Pronotum without whitish hairs; few pairs present on head dorsum from clypeus, and edge of frontal lobe to posterior portion of head; one pair on mesonotum; several pairs scattered on propodeal dorsum; petiolar node with whitish hairs arranged near lateral and dorsal borders of posterior face; scattered and much shorter erect hairs organized transversely on anterior and posterior portions of each gastral tergite; pubescence reduced. Body color black; antenna brown basally and dark brown apically; femur and tibia dark brown, trochanter and tarsi light brown.

**Major worker.** Unknown.

##### Distribution and biology.

This species is known from the rainforest of Ankerana and the PN Masoala (Fig. 45). In Masoala, individual workers have been collected only from the moss and leaf litter of the canopy while at Ankerana one worker was collected from a Malaise trap, suggesting a preference for canopy microhabitat.

##### Discussion.

*Camponotus tafo* is very similar to *Camponotus edmondi*, but the latter is characterized by a mesonotum with lateral margins that are roughly straight and gradually converge posteriorly in dorsal view. In *Camponotus edmondi*, the width of the propodeum at the metanotal groove is less than half the maximum width of the mesonotum; with head in full-face view, anteromedian margin of clypeus is truncate.

From the NC-clustering dendrogram, *Camponotus tafo* includes one sample of *Camponotus varatra* and *Camponotus zavo*, indicating that they are morphologically similar species. However, the confirmatory LDA successfully classified *Camponotus tafo* at 100%, with no additional samples from other species included. According to the qualitative morphology method, *Camponotus varatra* and *Camponotus zavo* can be separated from *Camponotus tafo* by their sculpture and nesting sites. In the two former species, the dorsum of the head and mesosoma is smooth and shining or imbricate. Their colony nests are built in dead twigs or branches slightly above the forest floor but never in the canopy. Thus, the separation of *Camponotus tafo* from both species is sustained.

#### Camponotus tratra

Taxon classificationAnimaliaHymenopteraFormicidae

Rakotonirina, Csősz & Fisher sp. n.

http://zoobank.org/DC902FCA-266A-42E6-9B57-DED5155930D8

**Figure 31.**
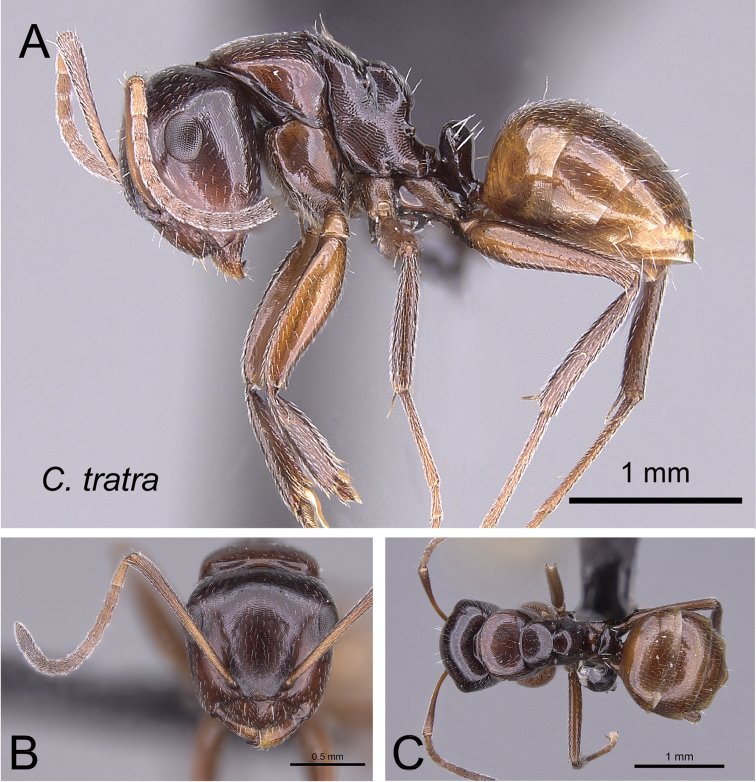
*Camponotus tratra* minor worker CASENT0153055. **A** lateral view **B** head in full-face view **C** dorsal view.

Figures 17A , 17B , 31 , 46

##### Holotype worker.

Madagascar, Province Toamasina, Parc National de Zahamena, Sahavorondrano River, –17.75257, 48.85725, 765 m, rainforest, beating low vegetation, 23 Feb 2009 (B.L. Fisher et al.) collection code: BLF22401, specimen code: CASENT0153055 (CASC).

##### Additional material examined.

**MADAGASCAR**: Province **Antsiranana**, Makirovana forest, –14.17066, 49.95409, 225 m, rainforest, (B.L. Fisher et al.) (CASC); Parc National Montagne d’Ambre [1st campsite], –12.51444, 49.18139, 960 m, rainforest, (R. Harin’Hala) (CASC); Réserve Spéciale Manongarivo, 10.8 km 229° SW Antanambao, –13.96167, 48.43333, 400 m, rainforest, (B.L. Fisher) (CASC); Province **Fianarantsoa**: 1 km E of Isalo National Park Interpretive Center, –22.62667, 45.35817, 885 m, dry wash (R. Harin’Hala) (CASC); stream area, 900 m E of Isalo National Park Interpretive Center, –22.62667, 45.35817, 750 m, open area near stream, (R. Harin’Hala) (CASC); Province **Toamasina**: Parc National Zahamena, Sahavorondrano River, –17.75257, 48.85725, 765 m, rainforest, (B.L. Fisher et al.) (CASC); Province **Toliara**: Parc National Andohahela, Col de Tanatana, 33.3 km NW Tolagnaro, –24.7585, 46.85367, 275 m, rainforest, (B.L. Fisher et al.) (CASC).

##### Diagnosis.

In profile, anterior and posterior margins of petiolar node convex; in profile, propodeal dorsum and declivitous surface separated by blunt angle; in dorsal view, mesonotum less than twice as broad as long; mesopleuron with propodeal surface together distinctly wider than lateral portion of pronotum; in profile, propodeal dorsum roughly as long as declivitous margin; dorsum of head and mesosoma smooth and shiny or imbricate; in profile, mesonotal dorsum strongly sloping down to the level of propodeum, maximum length of mesonotum about as long as distance between metanotal groove and propodeal spiracle; in dorsal view, lateral margin of mesonotum not well defined and converging gradually towards metanotal groove.

##### Description.

**Minor worker** (Figs 17A, 17B, 31). In full-face view head roughly as long as broad (CWb/CL: 0.91–0.97), lateral borders more or less straight and weakly diverging posteriorly; posterior cephalic margin broadly convex. Level of posterior ocular margins located at less than posterior fifth portion of head (PoOc/CL: 0.19–0.22). Anterior clypeal margin broadly convex. Mandible triangular, armed with six teeth. Antennal scape relatively long (SL/CS: 0.96–1.13), apical half almost surpassing posterior cephalic border. Pronotal dorsum flattened, anterodorsal angle projecting anteriorly narrow edge; dorsolateral portion without margination. In dorsal view, mesonotum less than twice as broad as long; lateral margin of mesonotum not well defined and converging gradually towards metanotal groove; in lateral view, mesonotal dorsum inclined posteriorly and lowering level of propodeum; length of mesonotum about as long as distance between metanotal groove and propodeal spiracle. In lateral view, dorsum of propodeum raised into a very short edge and then suddenly inclined posteriorly to join the declivitous surface. In lateral view, dorsolateral carina of propodeum weakly visible and roughly as long as declivitous margin; meso-metapleuron and lateral propodeal surface together distinctly broader than lateral portion of pronotum. Coxa of foreleg broad, maximum width greater than width of meso-metapleuron. In profile, anterior and posterior margins of petiolar node convex and rounding dorsal margin. Constriction between abdominal segments III and IV lacking.

Dorsum of head, mesosoma, petiolar node, and gastral tergite smooth and shining, superimposed with fine and dense imbrication and small punctures from which erect hairs and pubescence arise. Mandible sparsely punctulate. Pronotal dorsum without erect hairs; mesonotum with one pair, and propodeum with two or more pairs; erect hair lacking just above propodeal spiracle. Posterior face of petiolar node near lateral margin and posterodorsal angle with a row of four erect hairs; slender erect hairs arranged transversely on dorsum of petiolar node, on anterior and posterior portions of each gastral tergite. Body color generally brown, with much darker head, mesonotum, propodeum, and petiolar node.

**Major worker.** With characteristics of minor worker except for the following features: larger head (CS: 1.58) with straight rear margin; level of posterior margin of eyes located at about posterior third of head (PoOc/CL: 0.3); anterior clypeal margin transverse; antennal scape barely extending beyond posterior cephalic margin (SL/CS: 0.75); more robust mandible; two pairs or more of whitish erect hairs on dorsum of pronotum, mesonotum, and propodeum.

##### Distribution and biology.

Known only from Madagascar, *Camponotus tratra* has a sparse but wide distribution from Parc National Montgne d’Ambre in the north through Makirovana Forest in the northeast and Réserve Spéciale Manongarivo in the northwest, to Parc National Zahamena in the central east and Parc National Andohahela in the southeast (Fig. 46). Workers of this species have been sampled most often from low vegetation and rarely from leaf litter and one nest was found in dead branches above the ground.

##### Discussion.

*Camponotus tratra* is very similar to *Camponotus zavo* and *Camponotus varatra*, but the latter two species have a mesonotal dorsum slightly inclined posteriorly whose length is distinctly shorter than the distance between the metanotal groove and the propodeal spiracle in profile. Also, in the two latter species the lateral margins of the mesonotum are well defined and convex in dorsal view, converging strongly towards the metanotal groove.

The NC-clustering method groups all samples of *Camponotus tratra* together in the dendrogram with a classification success of 100%. However, one specimen of *Camponotus varatra* was placed in the *Camponotus tratra* cluster and was misclassified by confirmatory LDA with a posterior probability of 0.74. This may be due to the fact that both species are very closely related and have some overlap in their morphometric and qualitative descriptions. However, as discussed above, these species can be distinguished based on few qualitative morphological traits, one of which was not captured by the multivariate morphometric analysis. In addition, biological data for *Camponotus tratra* suggest that its nest sites are arboreal and could be located higher in the vegetation strata. By contrast, *Camponotus varatra* prefers nesting in dead branches above the ground or in lower vegetation.

#### Camponotus varatra

Taxon classificationAnimaliaHymenopteraFormicidae

Rakotonirina, Csősz & Fisher sp. n.

http://zoobank.org/5A74DE4D-5942-4047-A47A-E1547BEC2424

**Figure 32.**
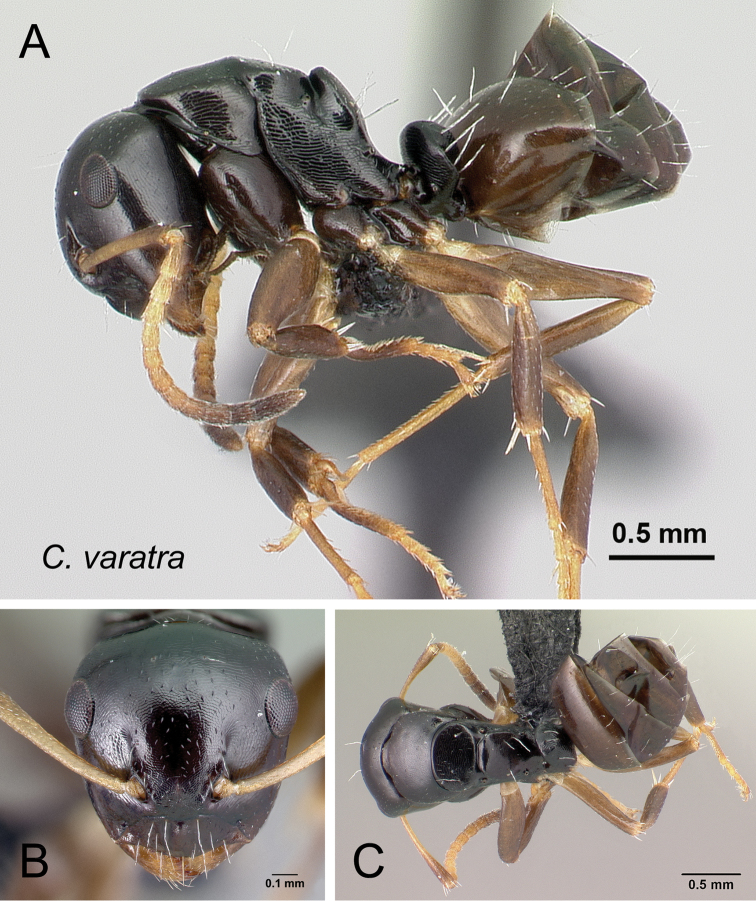
*Camponotus varatra* minor worker CASENT0492888. **A** lateral view **B** head in full-face view **C** dorsal view.

Figures 18C , 18D , 32 , 47

##### Holotype worker.

Madagascar, Province Fianarantsoa, Parc National de Ranomafana, Sahamalaotra River, 6.6 km 310° NW Ranomafana, –21.23667, 47.39667, 1150 m, montane rainforest, ex dead twig above ground, 31 Mar 2003 (Fisher, Griswold et al.) collection code: BLF08630, specimen code: CASENT0492888 (CASC).

##### Paratype.

1 dealate queen and 8 workers with same data as holotype but with the following specimen codes: CASENT0492886 (queen), CASENT0492887, CASENT0492889, CASENT0217289, CASENT0746977, CASENT0746978, CASENT0746979, CASENT0746980, CASENT0763748 (BMNH, MHNG, MSNG, CASC).

##### Additional material examined.

**MADAGASCAR**: Province **Antananarivo**: Mandraka Park, –18.9019, 47.90786, 1360 m, montane shrubland, (B.L. Fisher et al.) (CASC); Province **Antsiranana**: 6.9 km NE Ambanizana, Ambohitsitondroina, –13.56667, 50, 1080 m, montane rainforest, (B.L. Fisher) (CASC); Ampasindava, Forêt d’Ambilanivy, 3.9 km 181° S Ambaliha, –13.79861, 48.16167, 600 m, rainforest, (Fisher, Griswold et al.), (CASC); Forêt de Binara, 9.1 km 233° SW Daraina, –13.26333, 49.60333, 650–800 m, rainforest, (B.L. Fisher) (CASC); Parc National Montagne d’Ambre [1st campsite], –12.51444, 49.18139, 960 m, rainforest, (R. Harin’Hala) (CASC); Sakaramy, 07 Km N of Joffre Ville, –12.33333, 49.25, 360 m, low rainforest in open area, Campsite 2 of Fisher, (R. Harin’Hala) (CASC); Province **Fianarantsoa**: Belle Vue trail, Ranomafana National Park, –21.2665, 47.42017, 1020 m, mixed tropical forest, (R. Harin’Hala) (CASC); Vatovavy Fitovinany Region,District of Ifanadiana, 12 km W of Ranomafana, –21.25083, 47.40717, 1127 m, forest edge, open area, (Rin’Ha, Mike) (CASC); Parc National de Ranomafana, Sahamalaotra River, 6.6 km 310° NW Ranomafana, –21.23667, 47.39667, 1150 m, montane rainforest, (Fisher, Griswold et al.) (CASC); radio tower, Ranomafana National Park, –21.25083, 47.40717, 1130 m, forest edge, mixed tropical forest, open area, (M.E. Irwin, R. Harin’Hala) (CASC); Ranomafana National Park, Talatakely area, 0.4 km WSW of Park Entrance –21.41667, 47.68333, 900 m, mixed tropical forest, (D.H. Kavanaugh) (CASC); 9 km ESE Ranomafana, nr. Ifanadiana, –21.28333, 47.53333, 600 m, (P.S. Ward) (PSWC); Province **Toamasina**: Andasibe National Park, botanic garden near entrance, West of ANGAP office, –18.92639, 48.40783, 1025 m, tropical forest, (M.E. Irwin, R. Harin’Hala) (CASC); 1 km SSW Andasibe (=Perinet), –18.93333, 48.41667, 920 m, rainforest edge, (P.S. Ward) (PSWC); Ankerana, –18.40636, 48.80254, 1108 m, montane forest, (B.L. Fisher et al.) (CASC); Ankerana, –18.40829, 48.82107, 750 m, rainforest, (B.L. Fisher et al.) (CASC); Ankerana, –18.4017, 48.80605, 1035 m, montane forest, (B.L. Fisher et al.) (CASC); Betaolana Forest, along Bekona River, –14.52996, 49.44039, 880 m, rainforest, (B.L. Fisher et al.) (CASC); Corridor Forestier Analamay-Mantadia, Ambatoharanana, –18.80424, 48.40081, 968 m, rainforest, (B.L. Fisher et al.) (CASC); Corridor Forestier Analamay-Mantadia, Ambatoharanana, –18.79944, 48.40375, 1016 m, rainforest, (B.L. Fisher et al.) (CASC); Corridor Forestier Analamay-Mantadia, Ambatoharanana, –18.80438, 48.40735, 960 m, rainforest, (B.L. Fisher et al.) (CASC); Corridor Forestier Analamay-Mantadia, Ambohibolakely, –18.76131, 48.36437, 983 m, rainforest, (B.L. Fisher et al.) (CASC); Corridor Forestier Analamay-Mantadia, Tsaravoniana, –18.76465, 48.41938, 1039 m, rainforest, (B.L. Fisher et al.) (CASC); 16 km S Moramanga, –19.8333, 48.23333, 950 m, roadside, (P.S. Ward) (PSWC); Didy, [–18.19833, 48.57833] forêt (A. Pauly) (CASC); Station Forestiere, Tampolo, 10 km NNE Fenoarivo Atsinanana, –17.2825, 49.43, 10 m, littoral rainforest, (B.L. Fisher) (CASC); Province **Toliara**: Parc National Andohahela, Col de Tanatana, 33.3 km NW Tolagnaro, –24.7585, 46.85367, 275 m, rainforest, (B.L. Fisher et al.) (CASC); Parc National Andohahela, 6 km SSW Eminiminy,–24.73333, 46.8, 330 m, rainforest, (P.S. Ward) (PSWC).

##### Diagnosis.

In profile, anterior and posterior margins of petiolar node convex; in profile, propodeal dorsum and declivitous surface separated by blunt angle; in dorsal view, mesonotum less than twice as broad as long; mesopleuron with propodeal surface together distinctly wider than lateral portion of pronotum; in profile, propodeal dorsum roughly as long as declivitous margin; dorsum of head and mesosoma smooth and shiny or imbricate; in profile, mesonotum slightly sloping down to the level of propodeum, maximum length distinctly shorter than distance between metanotal groove and propodeal spiracle; in dorsal view, lateral margin of mesonotum well defined and evenly convex, converging abruptly towards metanotal groove; junction between dorsum and lateral surface of pronotum with sharp margination; no distinct angle between dorsal margin of propodeum and declivity; antennal scape and gastral segment with scattered appressed pubescence.

##### Description.

**Minor worker** (Figs 18C, 18D, 32). In full-face view head slightly longer than broad (CWb/CL: 0.83–0.96), sides slightly convex and noticeably diverging posteriorly; posterior cephalic margin generally convex. Level of posterior ocular margins located around or less than posterior fourth portion of head (PoOc/CL: 0.2–0.24). Anterior clypeal margin broadly convex. Mandible triangular, armed with six teeth. Antennal scape relatively short (SL/CS: 0.93–1.07), apical third extending beyond posterior cephalic border. Promesonotal dorsum flattened, dorsal face joining lateral portion with margination; anterodorsal angle of pronotum extending anteriorly into narrow edge. In dorsal view, mesonotum less than twice as broad as long; lateral margin evenly convex and abruptly converging posteriorly. In lateral view, propodeal dorsum extending into very short edge and then sloping strongly posteriorly to join the declivitous surface without an angle. In lateral view, dorsolateral carina of propodeum roughly as long as declivitous margin; meso-metapleuron and lateral propodeal surface together distinctly broader than side of pronotum. In side view, mesonotal length shorter than distance between metanotal groove and propodeal spiracle. Width of procoxa greater than width of meso-metapleuron combined. In profile, anterior petiolar margin convex, posterior margin sloping posteriorly to mid-height and descending almost vertically to posteroventral angle. Junction between abdominal segments III and IV without constriction.

Dorsum of head, mesosoma, petiole, and gastral tergites smooth and shiny or with imbricating sculpture. Sparse shallow punctures present on lateral portion of head near base of mandible. Mandible with sparse piligerous punctures. Numerous pairs of whitish erect hairs on dorsum of head arranged near lateral margin of clypeus and frontal carina to posteromedian portion of head. One pair of hairs on mesonotum and one to two pairs on propodeum at junction of its dorsum and declivity; pronotum and declivitous surface just above propodeal spiracle without erect hair. A row of four whitish hairs erected along near lateral margin and posterodorsal angle of posterior face of petiolar node. Anterior and posterior portions of each gastral tergite with transversely arranged, slender, erect hairs. Body with appressed, filiform, short pubescence; antennal scape covered with subdecumbent spatulate hairs. Body color generally black; gaster and appendages dark brown; basal portion of antenna, trochanter, and tarsus sometimes much lighter than remaining parts of appendages.

**Major worker.** Similar to minor worker, but differing in the following characters: head larger (CS: 1.35–1.62) and as long as broad (CWb/CL: 0.95–1), sides parallel to each other and suddenly converging to base of mandibles; posterior margin straight; small apical portion of antennal scape surpassing posterior margin of head (SL/CS: 0.66–0.78); level of posterior ocular margins at about posterior third of head (PoOc/CL: 0.24–0.29); mandible more robust; metanotum present between metanotal groove and propodeum; propodeal dorsum rounding to declivitous surface; one to two pairs of whitish erect hairs on pronotum, mesonotum, and propodeum.

##### Distribution and biology.

The species is endemic to Madagascar, where it is mostly found in montane forest habitats and rarely in lowland rainforests and littoral forest areas (Fig. 47). Its colonies frequently have been collected from dead twigs above ground and occasionally from rotten logs and dead tree stumps.

##### Discussion.

*Camponotus varatra* is separable from the similar species like *Camponotus zavo* because the latter species has the following combination of characters: the junction of the dorsum to the lateral portion of the pronotum is rounded, the junction between the dorsal margin of the propodeum and the declivity is either rounded or with a blunt angle, and the antennal scape and gastral segment are covered with abundant appressed pubescence.

Based on morphometric analysis, members of *Camponotus varatra* have been detected in the clusters of *Camponotus mifaka*, *Camponotus tafo*, and *Camponotus tratra* in the dendrogram. The cause of this phenomenon is unclear, but one possibility is that these species are very similar in morphology and several quantitative traits can overlap. Also, a few differentiating morphological characters, such as sculpture and pilosity, cannot be integrated into a quantitative morphometric study. For *Camponotus mifaka* and *Camponotus tafo*, the sculpture on the dorsum of their head and mesosoma has dense and fine reticulate punctures. Biologically, these species inhabit root mats in the ground and moss with leaf litter in the canopy, respectively.

#### Camponotus zavo

Taxon classificationAnimaliaHymenopteraFormicidae

Rakotonirina, Csősz & Fisher sp. n.

http://zoobank.org/9E5D4A34-7C31-427B-AE90-C5A19092CAB0

**Figure 33.**
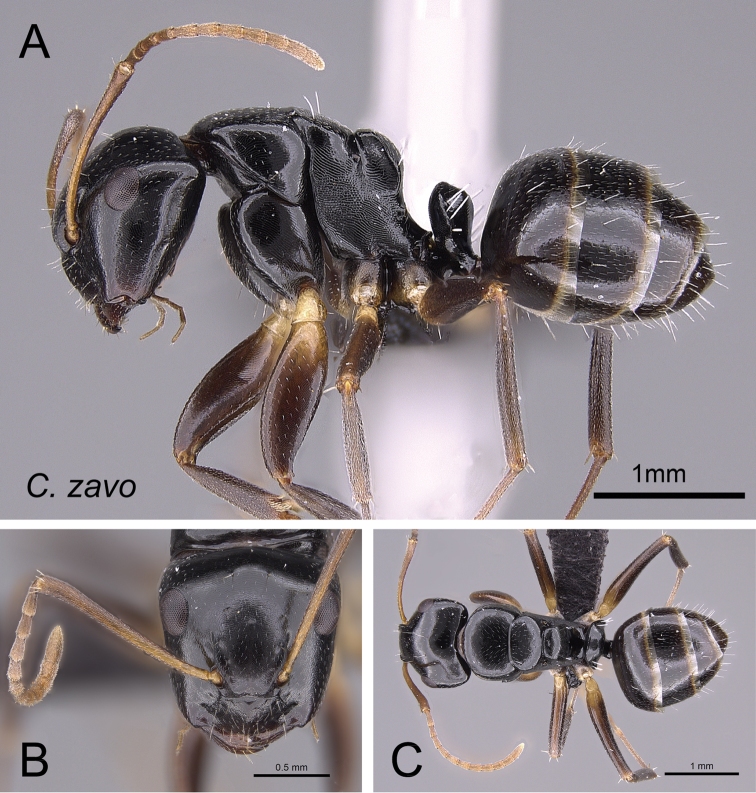
*Camponotus zavo* minor worker CASENT0060041. **A** lateral view **B** head in full-face view **C** dorsal view.

Figures 5B , 10C , 17C , 17D , 18A , 18B , 33 , 48

##### Holotype worker.

Madagascar, Province Fianarantsoa, Forêt Classée Vatovavy, 7.6 km 122º Kianjavato, –21.4, 47.94, 175 m, rainforest, on low vegetation, 6–8 Jun 2005 (B.L. Fisher et al.) collection code: BLF12401, specimen code: CASENT0060041 (CASC).

##### Paratype.

1 worker same data as holotype but with specimen code CASENT0060040 (CASC).

##### Additional material examined.

**MADAGASCAR**: Province **Antananarivo**: Forêt de galerie, Telomirahavavy, 23.4 km NNE Ankazobe, –18.12167, 47.20627, 1520 m, disturbed gallery montane forest, (B.L. Fisher et al.) (CASC); Province **Antsiranana**: SAVA Region, District of Sambava, Marojejy National Park, 5 km W of Manantenina village, 1st Campsite (*Mantella*), –14.43817, 49.774, 487 m, low altitude rainforest, (Rin’Ha, Mike) (CASC); Province **Fianarantsoa**: 7.6 km 122º Kianjavato, Forêt Classée Vatovavy, –21.4, 47.94, 175 m, rainforest (B.L. Fisher et al.) (CASC); Vatovavy Fitovinany Region, District of Ifanadiana, 12 km W of Ranomafana, –21.25083, 47.40717, 1127 m, forest edge, open area, (Rin’Ha, Mike) (CASC); Forêt de Vevembe, 66.6 km 293° Farafangana, –22.791, 47.18183, 600 m, rainforest, transition to montane forest, (B.L. Fisher et al.) (CASC); radio tower, Ranomafana National Park, –21.25083, 47.40717, 1130 m, forest edge, mixed tropical forest, open area, (M.E. Irwin, R. Harin’Hala) (CASC); Vohiparara broken bridge, –21.22617, 47.36983, 1110 m, high altitude rainforest, (R. Harin’Hala) (CASC); Province **Toamasina**: 5.3 km SSE Ambanizana, Andranobe, –15.66667, 49.96667, 600 m, rainforest, (B.L. Fisher) (CASC); 7 km SE Andasibe National Park Headquarters, –18.96278, 48.45267, 1050 m, tropical forest, (M.E. Irwin, R. Harin’Hala) (CASC); Province **Toliara**: 13 km NW Enakara, Parc National Andohahela, –24.55, 46.8, 1150 m, montane rainforest, (B.L. Fisher) (CASC).

##### Diagnosis.

In profile, anterior and posterior margins of petiolar node convex; in profile, propodeal dorsum and declivitous surface separated by blunt angle; in dorsal view, mesonotum less than twice as broad as long; mesopleuron with propodeal surface together distinctly wider than lateral portion of pronotum; in profile, propodeal dorsum roughly as long as declivitous margin; dorsum of head and mesosoma smooth, shiny, and superimposed by imbrication; in profile, mesonotum slightly sloping down to the level of propodeum, maximum length distinctly shorter than distance between metanotal groove and propodeal spiracle; in dorsal view, lateral margin of mesonotum well defined and evenly convex, converging abruptly towards metanotal groove; junction of pronotal dorsum to lateral surface always rounded; blunt angle between dorsal margin of propodeum and declivity distinct; antennal scape and gastral segment covered with abundant appressed pubescence.

##### Description.

**Minor worker** (Figs 5B, 10C, 17C, 17D, 18A, 18B, 33). In full-face view head subquadrate (CWb/CL: 0.93–1), sides approximately straight and slightly diverging posteriorly; posterior border medially convex and nearly straight towards corners. Level of posterior ocular margins located around or lower than posterior fifth portion of head (PoOc/CL: 0.18–0.23). Anterior clypeal margin straight. Mandible triangular, armed with six teeth. Antennal scape relatively long (SL/CS: 1–1.22), distal portion extending beyond posterior cephalic border. Anterodorsal angle of pronotum extending anteriorly into narrow edge, but dorsolateral portion without longitudinal margination, junction of dorsum to lateral surface rounded. In dorsal view, mesonotum narrow, less than twice as broad as long; lateral margin well defined and evenly convex, converging abruptly toward metanotal groove. In profile, propodeum not strongly compressed anteroposteriorly, propodeal dorsum with a short, more or less horizontal edge and suddenly sloping posteriorly to join the declivitous surface at a blunt angle. In profile, dorsolateral carina of propodeum as long as its posterolateral margin; width of mesopleuron and propodeal surface combined distinctly greater than width of lateral portion of pronotum. In profile, maximum length of mesonotum distinctly shorter than distance between metanotal groove and propodeal spiracle, which is located on declivitous surface. Maximum width of coxa of foreleg greater than width of meso-metapleuron combined. In profile, anterior margin of petiolar node convex and posterior margin inclined posteriorly until mid-height and descending almost vertically to posteroventral angle. Constriction between abdominal segments III and IV lacking.

Dorsum of head, mesosoma, petiole, and gastral tergites smooth and shiny with superimposed imbricating sculpture. Lateral portion of head near base of mandible with scattered shallow punctures. Mandible smooth and shining between sparse punctures. Whitish erect hairs arranged as follows: several pairs on clypeus, three pairs on frontal carina, one pair at level of eyes and one pair on posterior portion of head dorsum; lacking on pronotum; one to two pairs on mesonotum; numerous hairs on propodeum arranged in a row along junction of propodeal dorsum and declivity; lacking on declivitous face just above spiracle; a few pairs arranged in a row near lateral margin and posterodorsal angle of petiolar posterior face; sparse and slender erect hairs arranged transversely on anterior and posterior portions of each gastral tergite. Appressed pubescence present, abundant on antennal scape. Head, mesosoma, and petiole black; gaster dark brown; appendages brown to light brown.

**Major worker.** Similar to minor worker, but differing in the following characters: head slightly wider than long (CWb/CL: 1.01–1.04), posterior margin almost straight; antennal scape hardly surpassing posterior cephalic border (SL/CS: 0.76–0.82); level of posterior ocular margins at about posterior third portion of head (PoOc/CL: 0.24–0.27); mandible more robust with accentuated microreticulation basally and microreticulate punctate near apical margin; visible metanotum between metanotal groove and propodeum; one to two pairs of whitish erect hairs on pronotum and more on mesonotum and propodeum.

##### Distribution and biology.

This species is widely distributed in eastern Madagascar and occupies a wide array of habitats ranging from lowland rainforest at 175 m of elevation to montane forest habitats up to 1520 m in elevation (Fig. 48). Foraging is carried out on lower part of vegetation and nests are built in dead twigs above the ground.

##### Discussion.

*Camponotus zavo* is very similar to the sympatric species *Camponotus varatra* in that in both, the integument is smooth and shining or imbricate and the lateral margins of the mesonotum are well-defined and evenly convex. However, *Camponotus varatra* can be distinguished by the fact that it has no distinct angle separating the propodeal dorsum and the declivitous margin in lateral view and the dorsum and the lateral surface of the pronotum are separated by a sharp angle.

**Figures 34–39.**
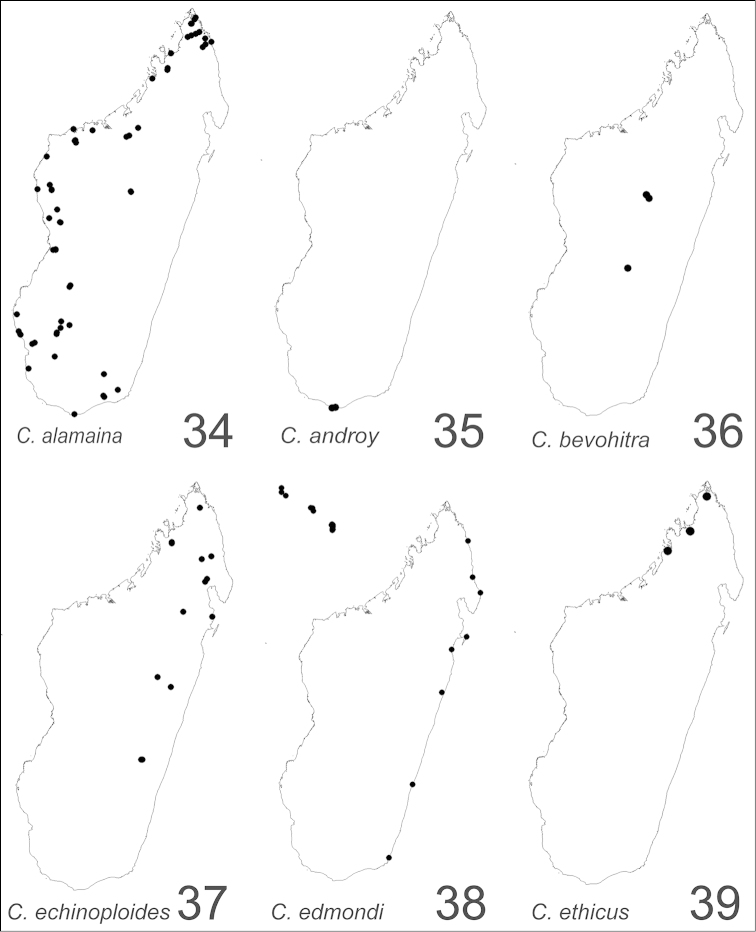
Distribution maps of the *Camponotus edmondi* species group in the Malagasy region.

**Figures 40–45.**
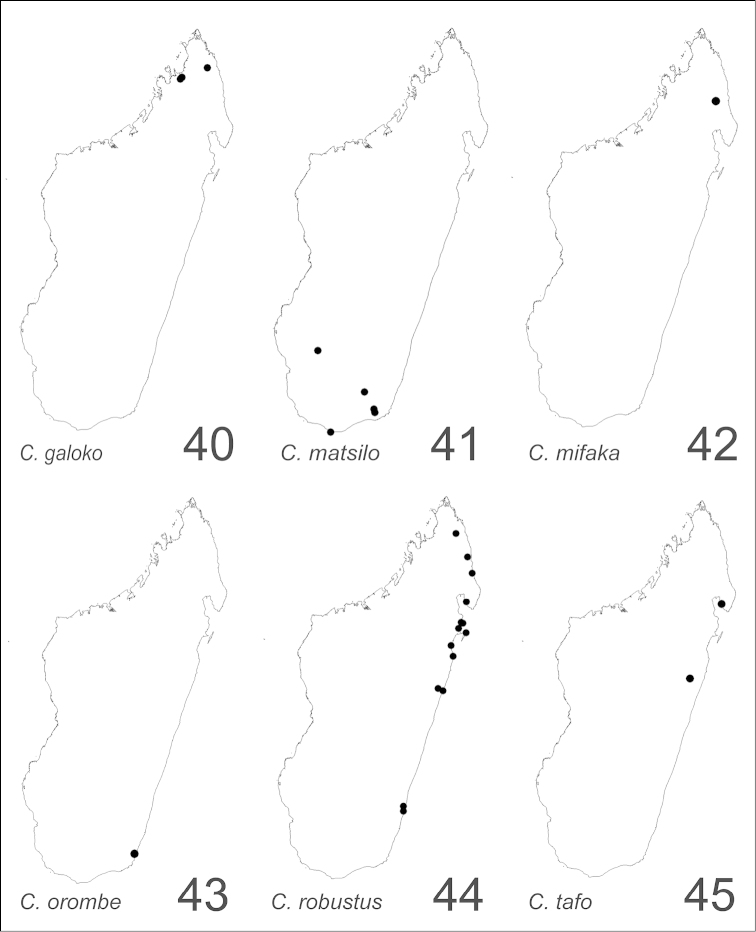
Distribution maps of the *Camponotus edmondi* species group in the Malagasy region.

**Figures 46–48.**
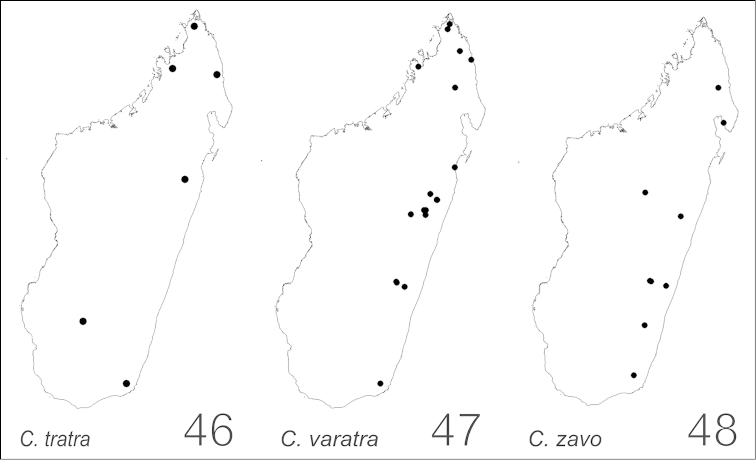
Distribution maps of the *Camponotus edmondi* species group in the Malagasy region.

## Supplementary Material

XML Treatment for Camponotus alamaina

XML Treatment for Camponotus androy

XML Treatment for Camponotus bevohitra

XML Treatment for Camponotus echinoploides

XML Treatment for Camponotus edmondi

XML Treatment for Camponotus ethicus

XML Treatment for Camponotus galoko

XML Treatment for Camponotus matsilo

XML Treatment for Camponotus mifaka

XML Treatment for Camponotus orombe

XML Treatment for Camponotus robustus

XML Treatment for Camponotus tafo

XML Treatment for Camponotus tratra

XML Treatment for Camponotus varatra

XML Treatment for Camponotus zavo

## Supplementary materials

Supplementary material 1 Measurements of individual specimens Data type: specimens data Explanation note: Basic measurements of individual specimens arranged by species code, collection code, and specimen code (unique identification number). See text for abbreviations. This dataset is made available under the Open Database License (http://opendatacommons.org/licenses/odbl/1.0/). The Open Database License (ODbL) is a license agreement intended to allow users to freely share, modify, and use this Dataset while maintaining this same freedom for others, provided that the original source and author(s) are credited.Jean Claude Rakotonirina, Sándor Csősz, Brian L. Fisher
